# Two Routes to Land: Genomic Underpinnings of Parallel Aerial Egg Deposition in Aquatic Old‐World *Pila* and New‐World *Pomacea* (Ampullariidae)

**DOI:** 10.1002/advs.202522371

**Published:** 2026-06-02

**Authors:** Yufei Zhou, Huawei Mu, Xueying Nie, Yue Gao, Hui Wang, Ling Fang, Tiangang Luan, Monthon Ganmanee, Jian‐Wen Qiu, Jin Sun, Jack Chi‐Ho Ip

**Affiliations:** ^1^ Key Laboratory of Evolution & Marine Biodiversity (Ministry of Education) and Institute of Evolution & Marine Biodiversity Ocean University of China Qingdao China; ^2^ Laboratory For Marine Biology and Biotechnology Laoshan Laboratory Qingdao Marine Science and Technology Center Qingdao China; ^3^ Division of Science Lingnan University Hong Kong SAR China; ^4^ Hefei National Research Center for Physical Sciences at the Microscale Division of Life Sciences and Medicine University of Science and Technology of China Hefei China; ^5^ Anhui Province Key Laboratory of Biomedical Imaging and Intelligent Processing Institute of Artificial Intelligence Hefei Comprehensive National Science Center Hefei China; ^6^ Instrumental Analysis & Research Center Sun Yat‐Sen University Guangzhou China; ^7^ State Key Laboratory of Biocontrol School of Life Sciences Sun Yat‐Sen University Guangzhou China; ^8^ School of Environmental and Chemical Engineering Wuyi University Jiangmen China; ^9^ Department of Animal Production Technology and Fisheries Faculty of Agricultural Technology King Mongkut's Institute of Technology Ladkrabang Bangkok Thailand; ^10^ Department of Biology Hong Kong Baptist University Hong Kong SAR China

**Keywords:** aerial oviposition, ampullariidae, horizontal gene transfer, parallel evolution, terrestrialization

## Abstract

The evolution of aerial oviposition in Old‐World *Pila* and New‐World *Pomacea* apple snails—diverged since the Gondwanan breakup—offers a powerful model for probing genomic adaptations underpinning key evolutionary innovations. We generate a chromosomal‐level genome for *Pila celebensis* and a scaffold‐level genome for *Pila pesmei*, revealing a genus‐specific doubling in genome size driven by transposable element expansions. Analyses of macrosynteny and topologically associating domains (TAD) identified lineage‐specific chromosomal rearrangements associated with positive selection in gene blocks enriched for environmental sensing, metabolism, and stress response. Breakpoints in aerial egg layers preferentially are localized within TADs, suggesting convergent rewiring of gene regulation. Gene family evolution revealed parallel expansions in cellulases, β‐D‐xylosidases, and immune genes, alongside convergent positive selection in aquaporins critical for aerial osmoregulation. Perivitelline fluid (PVF) proteomics uncovered the central role of PVF1, likely acquired via ancient horizontal gene transfer (HGT) from viruses in the Ampullariidae ancestor in the Jurassic. Subsequent duplications enabled lineage‐specific adaptation; PVF1 in aerial eggs shows parallel increases in hydrophobicity and aromatic residues (notably phenylalanine), enhancing desiccation resistance. Collectively, these convergent genomic mechanisms—structural rearrangement, gene family dynamics, and HGT‐driven innovation—underpin the independent evolution of aerial oviposition in *Pila* and *Pomacea*, providing a multi‐layered blueprint for understanding key ecological transitions.

## Graphical abstract

Comparative genomics of Gondwana‐diverged *Pila* and *Pomacea* reveals parallel evolution of aerial oviposition. Convergent chromosomal rearrangements reshape regulatory landscapes within topologically associating domains. Lineage‐specific gene family expansions and viral‐derived perivitelline proteins (PV1) underpin desiccation resistance. Multi‐layered genomic adaptations—structural, regulatory, and functional—illuminate mechanisms driving ecological transitions.

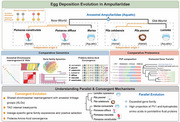

## 1 Introduction

Evolutionary transition from water to land, termed terrestrialization, represents one of the most remarkable events that have profoundly shaped Earth's biodiversity [1]. In animals, this shift occurred independently across at least nine phyla (chordates, nematodes, tardigrades, arthropods, onychophorans, platyhelminthes, molluscs, annelids, and nemerteans) over the past 420 million years [2]. A core biological question is how genomes adapted to the distinct physical and ecological challenges during the transition to land. In vertebrates, several mechanisms have been proposed: transposable elements (TEs) proliferation, which rewired genome structure and regulation, enabling innovations such as limb development [3]; gene family dynamics and co‐option, which generated novel genetic material for traits such as aerial respiration and desiccation tolerance [4]; and the evolution of novel regulatory networks, such as new Hox cluster linkages involved in the fin‐to‐limb transition, as observed in lungfish [5]. Similarly, in invertebrates, genomic studies have provided insights into terrestrialization. For example, Vargas‐Chávez et al. analyzed chromatin structures in marine, freshwater, and terrestrial annelids, while Aristide et al. investigated ∼100 mollusc genomes to explore independent transitions from marine to terrestrial environments [6, 7]. Together, these studies lay the foundation for understanding the genomic basis of terrestrial adaptations across diverse animal groups.

Gastropods (Mollusca) are exceptional models for dissecting invertebrate terrestrialization genomics, having independently colonized land at least eight times, often via freshwater or brackish routes [8, 9]. Many terrestrial snails have evolved behavioral and metabolic adaptations to maintain thermoregulation and water balance, such as seeking shaded habitats, burrowing into soil, and modulating shell size and pigmentation [10, 11], whereas some species also have specialized mantle opening for aerial respiration [8, 12]. At the cellular level, terrestrial snails exhibit an enrichment in chaperones and antioxidant enzymes, alongside responses in mucocyte and digestive gland cells, facilitating their transition to land [13]. Within gastropods, apple snails (Ampullariidae) serve as ideal models for understanding the transition from water to land [14, 15, 16], much like the role of lungfish in tetrapod evolution. This family is phylogenetically significant, being basal to caenogastropods and a sister clade to land snails (Cyclophoroidea) and freshwater snails (Viviparidae) within Architaenioglossa [17], with a crucial trait for the terrestrialization—their ability to retain an aquatic adult life while evolving aerial oviposition [14, 15]. Like the evolutionary transition of insects from aquatic to terrestrial environments, changes in egg structure represent a key adaptation [18].

**FIGURE 1:**
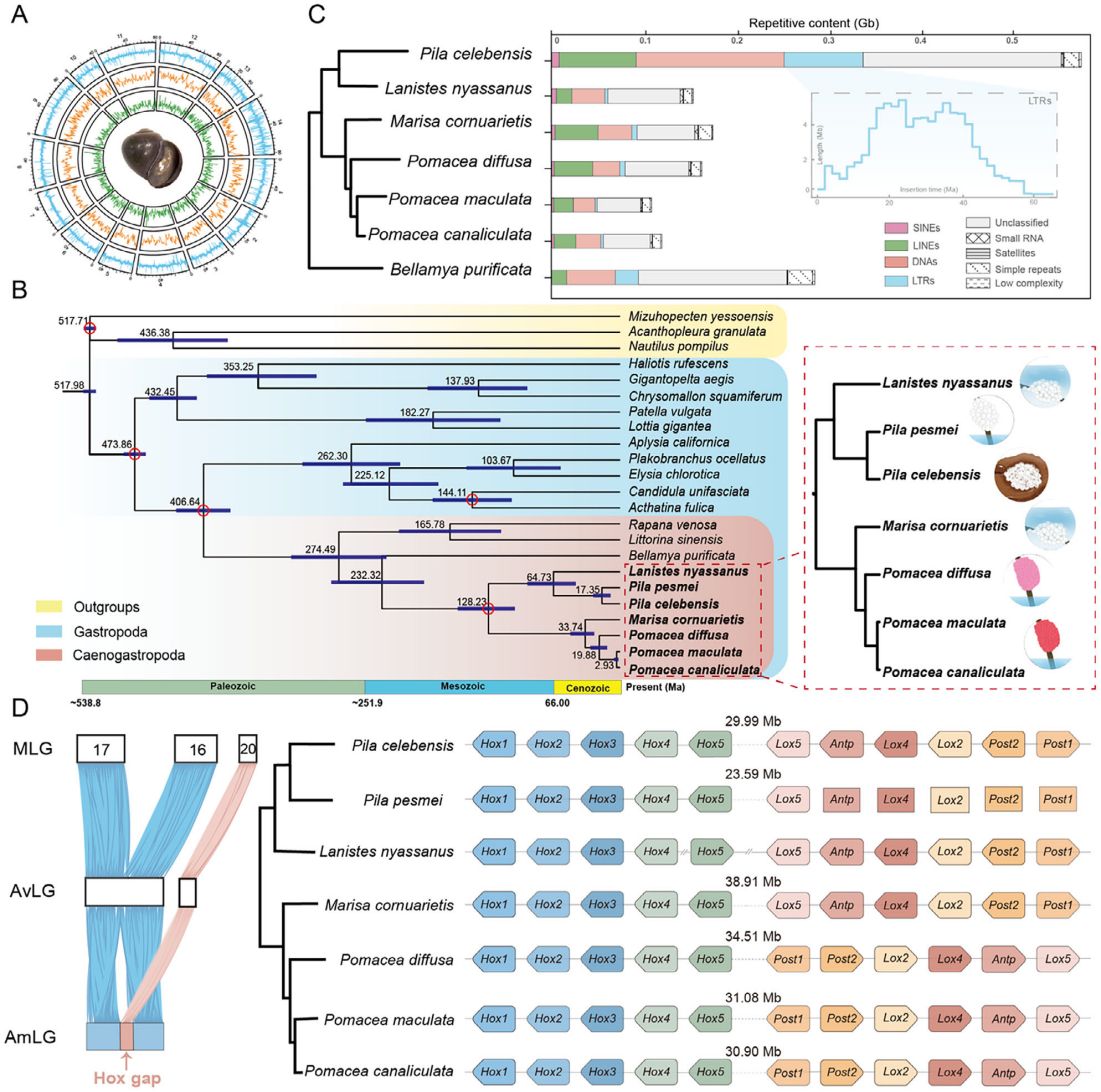
Genome landscape and evolutionary dynamics in *Pila*. (A) Circos plot of 14 linkage groups (corresponding to “chromosome”) showing marker distributions in 1 Mb sliding windows from outer to inner circle: Illumina sequencing depth, GC content and gene density, with the shell shape of *Pila celebensis* at the center. (B) Maximum‐likelihood phylogenetic tree of 23 molluscs, including cephalopoda, Bivalvia, and polyplacophora as outgroups (left). The tree was calibrated at five nodes (indicated by red dots) using fossils and geological events, revealing divergence times with 100% bootstrap support; dark‐purple lines indicate 95% confidence intervals. The circular diagram in the Ampullariidae tree (right) illustrate aerial and aquatic egg laying in Ampullariidae family. (C) Comparison of repetitive elements showing expansion of four transposable element classes (i.e., SINEs, LINEs, LTRs, and DNAs) in the Ampullariidae and *Bellamya purificata* genomes. Estimated LTR insertion times for *Pila celebensis* are indicated within a gray dotted border. (D) Schematic of *Hox* gene clusters in seven Ampullariid species, illustrating *Ho*x‐linked chromosomes evolution. Gene names are labeled centrally, with connections indicating scaffolds; line lengths do not represent sequence lengths. The symbol “//” indicates a break between different scaffolds, and transcription direction is indicated by a bullet head. Ancestral linkage groups are denoted as MLG (Mollusca), AvLG (Ampullariidae and Viviparidae), and AmLG (Ampullariidae).

Currently, the family Ampullariidae comprises nine genera with approximately 120 valid species distributed throughout freshwater ecosystems in tropical and subtropical regions of Africa, the Americas, and Asia [19, 20]. Within this family, the most diverse Old‐World genus *Pila* and New‐World genus *Pomacea* stand out for their remarkable parallel evolutionary histories—both have independently transitioned from underwater to aerial egg‐laying. In Ampullariidae, aerial oviposition involves depositing eggs above the water line, such as on emergent vegetation, hard substrates, or damp soil (Figure 1B) [20, 21, 22]. Despite this similarity, the specific methods of aerial egg‐laying remain diverse between and within *Pila* and *Pomacea*. For example, *Pila celebensis* lays eggs in moist soil, *Pila pesmei* prefers hard vegetative branches, while *Pomacea* species lay eggs on hard substrates above the water (Figure 1B). Adapting to aerial oviposition necessitates overcoming key terrestrial challenges, including desiccation, osmoregulation, UV exposure, and novel predation/pathogen pressures [23]. These are reflected in traits like calcareous eggshells and specialized perivitelline fluid proteins (PVFs) [15, 24]. Thus, *Pila* and *Pomacea* offer a unique comparative framework: two phylogenetically distinct lineages separated since the breakup of Gondwana, which have independently evolved a key terrestrialization trait (aerial reproduction) while otherwise remaining aquatic. Here, we leverage comparative genomics of these genera to elucidate the genomic architecture underpinnings of their parallel evolution toward terrestrial reproduction.

Our previous genomic studies have generated valuable resources for studying Ampullariid evolution, including high‐quality genomes for the genera *Lanistes*, *Pomacea*, and *Marisa* [14, 25, 26]. While those studies shed light on the role of egg neurotoxins and calcareous‐related proteins in the New‐World Canaliculata clade (*Pomacea canaliculata* and *P. maculata*), the genetic basis of aerial reproduction remains largely unexplored in the Old‐World genus *Pila* and, more broadly, in the context of parallel evolution between Old and New‐World Ampullariidae species. To fill this knowledge gap, we generated a chromosomal‐level genome assembly for *Pila celebensis* and a scaffold‐level assembly for *Pila pesmei*, alongside a characterization of the egg perivitelline fluid (PVF) proteome in *Pila*. Our comparative analyses of these parallel‐evolving aerial reproductive genera reveal dynamic patterns of gene evolution across the genome, providing foundational insights into the molecular mechanisms that have enabled the water‐to‐land transition in molluscs.

## 2 Results and Discussion

### 2.1 Assembly and Analysis of Two *Pila* Genomes Reveal Repetitive Sequence Expansion Driving Genome Doubling

K‐mer analysis of Illumina reads (Table S1) estimated a genome size of 921.5 Mb for *Pila celebensis* and 925.8 Mb for *Pila pesmei*, approximately twice the size of Old‐World *Lanistes* and New‐World *Marisa* and *Pomacea* spp. [14, 25]. Assembly of PacBio HiFi reads resulted in 406 scaffolds for *Pila celebensis*, with an N50 of 9,853 Kb and a mean length of 2,578 Kb. Incorporating Hi‐C data, 96.60% of the total genome assembly was anchored to 14 chromosomes, with the final genome size of 1,048 Mb (Figure 1A; Figure S1; Table S2). BUSCO analysis against metazoan_db10 BUSCOs showed 95.80% complete of *Pila celebensis*, which is comparable with newly published Ampullariidae and other molluscan genomes (Table S2) [25]. Due to the limited samples, a hybrid assembly of Illumina reads and ONT long reads produced a draft *Pila pesmei* genome with a total length of 898 Mb (Table S2). Although the *Pila pesmei* genome has a relatively low assembly score of 87.70% metazoan BUSCOs, the high mapping rate of >98% for Illumina short reads and >90% ONT reads indicate the integrity of the assembly (Table S1). In addition, a total of 24,954 and 29,777 protein‐coding genes (PCGs) were predicted in the genomes of *Pila celebensis* and *Pila pesmei* with BUSCO scores of 97.20% and 86.70%, respectively (Table S2). The variation in predicted gene numbers (24,954 in *Pila celebensis* vs. 29,777 in *Pila pesmei*) likely reflects the difference in assembly contiguity, where the scaffold‐level assembly of *Pila pesmei* may lead to fragmented gene models, compared to the high‐quality chromosome‐level assembly of *Pila celebensis*.

Synonymous substitution rate (Ks) analysis revealed no whole‐genome duplication in the genus *Pila* (Figure S2) [27]. To explain the doubled genome size of *Pila*, we compared repeat content across Ampullariidae and Viviparidae (Figure 1C; Table S3). We found that both *Pila celebensis* and *Pila pesmei* exhibit substantially higher repetitive content (577.3 Mb, 55.08% of a 1.05 Gb genome and 466.6 Mb, 51.98% of an 898 Mb genome, respectively) compared to *Lanistes* (153.7 Mb, 30.14%), *Marisa* (174.0 Mb, 32.24%), and *Pomacea* species (∼148.0 Mb, ∼27.06%; Table S3). Notably, the non‐repetitive DNA is conserved across taxa, confirming that the genome size increase in the *Pila* lineage is primarily driven by the proliferation of repetitive elements. Analysis of transposable elements (TEs) revealed consistent expansion dynamics across the *Pila* genus (Figure 1C; Figure S3; Table S3). Specifically, Long Terminal Repeats (LTRs) and Long Interspersed Nuclear Elements (LINEs) underwent exceptional expansion in both *Pila* species compared to the averages observed in other Ampullariidae species (LTRs: 69.7–85.0 Mb in *Pila* vs. ∼6.8 Mb average; LINEs: 42.0–73.2 Mb in *Pila* vs. ∼24.2 Mb average). Similar expansions have been documented to have functional significance in other mollusks and plants. For instance, in the limestone‐dwelling land snail *Oreohelix idahoensis*, LTRs contribute to genomic features and ectopic recombination, while in the Spanish slug *Arion vulgaris*, LINEs are linked to invasive plasticity and stress resistance [28, 29]. Similarly, LTRs bursts in cotton *Gossypium rotundifolium* not only increase genome size and intergenic spacing but also affects higher‐order chromosomal structures [30]. While the expansion of LINEs and LTRs in *Pila* coincides with its ecological diversification, whether these TEs contribute to environmental adaptability, potentially through gene structural remodeling or the generation of novel alleles, remains a hypothesis that requires further functional validation.

### 2.2 Gondwanan Breakup–Driven Global Divergence and Adaptive Evolution of Ampullariidae Revealed by Macrosynteny Analysis

We constructed a maximum‐likelihood phylogenetic tree of seven Ampullariids and 16 other molluscs using 7,716 orthologous genes (Figure 1B; Table S2). The topology, with full bootstrap support (100) for all nodes, recovered major Ampullariidae lineages— Caenogastropoda contains Ampullariidae and as sister group to Heterobranchia— consistent with our prior phylogenetic hypotheses [14]. Fossil‐calibrated divergence estimates the Old‐World Ampullariid lineage (*Lanistes* and *Pila*) and New‐World Ampullariid lineage (*Marisa* and *Pomacea*) split at 128.2 Ma (95% confidence interval of 101.5 – 155.5 Ma), aligning with Gondwana breakup at ∼120 Ma [31]. Within the Old‐World lineage, *Lanistes* and *Pila* diverged ∼64.7 Ma (45.4 – 91.0 Ma), likely facilitated by land‐bridge dispersals during Indian‐African plate convergence (Late Cretaceous to Palaeocene; 60–70 Ma) [32]. The New‐World lineage split between *Marisa* and *Pomacea* occurred ∼33.7 Ma (24.6 – 46.2 Ma), potentially initiated by Eocene Andean orogeny (40–50 Ma). This timing coincides with Miocene ecological shifts in Amazonia's Lake Pebas, a proposed cradle for freshwater mollusc evolution [33, 34]. In *Pomacea*, the Bridgesii clade (*P. diffusa*) and Canaliculata clade (*P. canaliculata* and *P. maculata*) diverged ∼19.9 Ma (13.5 – 28.2 Ma), while *P. canaliculata* and *P. maculata* split ∼3.0 Ma (1.9 – 4.7 Ma)—consistent with the documented introgressive hybridization between two species [35].

Macrosynteny analysis provides insights into species adaptation [36]. While the genome of *Pila* exhibits a conserved haploid karyotype (n = 14), consistent with other Ampullariidae species, it also reveals five key fusion events derived from 20 ancestral molluscan linkage groups (MLGs) (Figures S4 and S5) [37]. Rearranged genes were identified by comparing the gene order of extant Ampullariidae species against the reconstructed Ancestral Linkage Groups (ALGs). Specifically, we defined rearranged genes as those residing within large‐scale inversion blocks, which are visually characterized by crossing red lines in supplementary m (Figure S6–S9 and Table S4). The genes situated within these rearranged regions are predominantly associated with environmental sensing and adaptation (Table S5), such as regulators of G‐protein signaling, where G‐protein‐coupled receptors (GPCRs) mediate chemical signal perception in aquatic environments, and rearrangements of synaptotagmin genes, known to enhance neural circuit complexity and learning behaviors in *Aplysia* [38]. Additional rearranged genes are associated with signal regulation, energy metabolism, and physiological activities (Table S5), further supporting the hypothesis that chromosomal rearrangements have facilitated the evolutionary adaptation of Ampullariidae to complex habitats.

In addition to environmental adaptation, we identified a fusion event that significantly altered the arrangement of *Hox* genes in Ampullariidae. All seven Ampullariidae genomes retain the ancestral molluscan complement of 11 *Hox* and three *ParaHox* genes, only *Pomacea* lineage shows an inversion from *Lox*5‐*Post*1 to *Post*1‐*Lox*5 (Figure S10). Specifically, a complex fusion involving MLG17, MLG16, and MLG20 (Figure 1D; Table S6) led to a ∼30 Mb inter‐cluster gap between two *Hox* clusters in Ampullariidae. MLG16 and MLG17 underwent a fusion event from MLG to the common ancestor of Ampullariidae and Viviparidae, while MLG20 remained independent. Subsequently, in the ancestor of Ampullariidae, MLG20 inserted into the middle of the fused MLG16/MLG17 region, generating the ∼30 Mb gap between the two *Hox* clusters (Figure 1D). This gap region harbors numerous genes with functional roles in energy metabolism (e.g., glyceraldehyde‐3‐phosphate dehydrogenase and succinate dehydrogenase), gene expression regulation, protein synthesis, and signal transport, many of which show high expression levels (Table S6). In *Corbicula* clams exposed to low salinity stress for 72 h, transcriptome analysis revealed a significant enrichment of significantly down‐regulated genes on the fused chromosome, suggesting possible regulatory shifts associated with fusion [39]. In this study, we identified a chromosomal rearrangement event and observed that genes located within the resulting gap regions exhibited exceptionally high expression levels. Although these findings suggest a potential link between structural variation and gene expression, whether this insertion directly drives shifts in the regulatory landscape or confers a fitness advantage for environmental responsiveness remains to be validated through experiments.

### 2.3 Convergent Chromosomal Rearrangements in *Pila* and *Pomacea*

**FIGURE 2:**
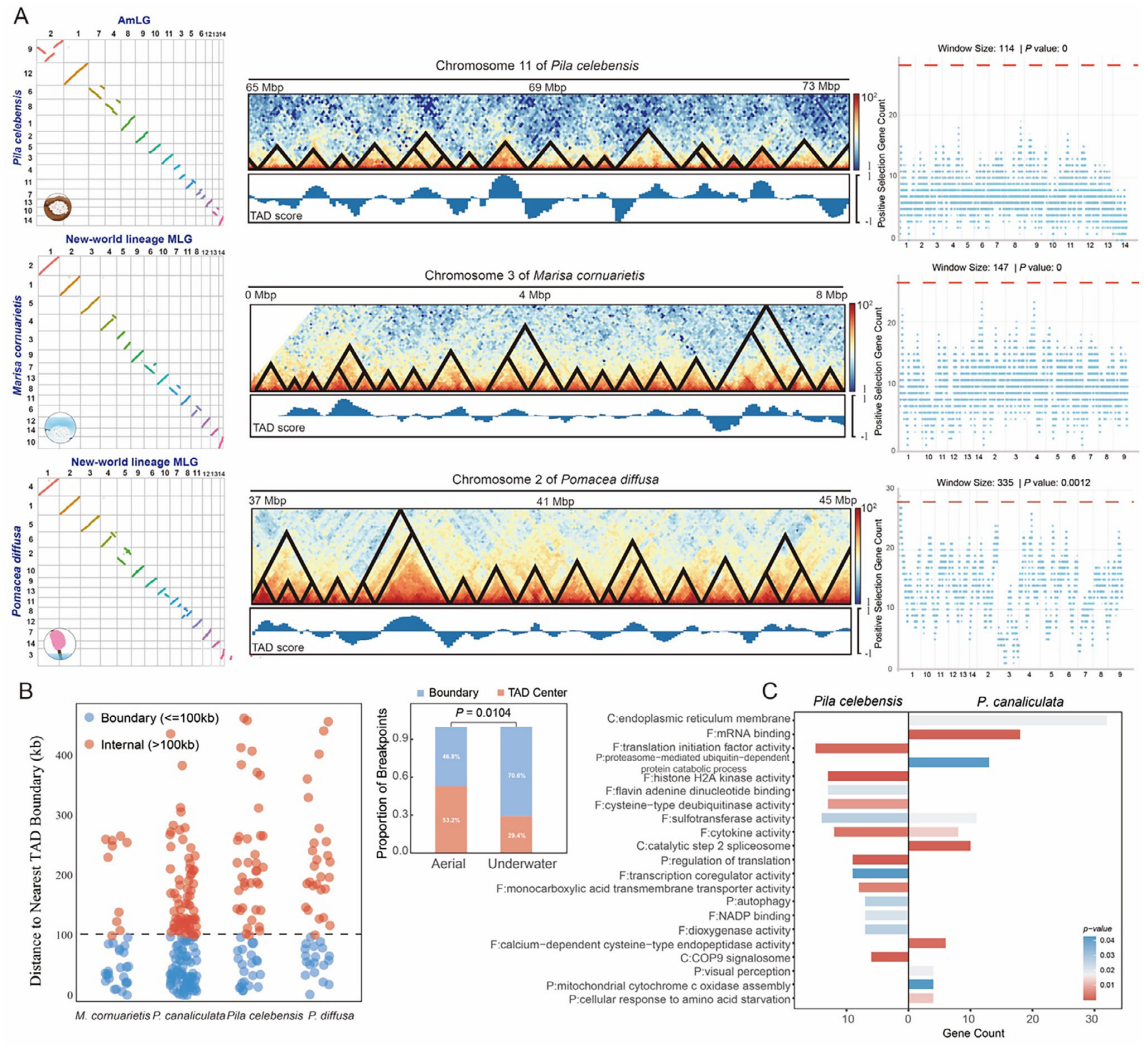
Synteny evolution and genome architecture organization in Ampullariidae. (A) Left, Oxford dotplots of the chromosome‐scale ancient gene linkage in Ampullariidae ancestor (AmLG) and New‐world lineage ancestor (MLG) compared with two aerial egg‐laying species (*Pila celebensis* and *P. diffusa*) and one under water egg‐laying species (*M. cornuarietis*). Middle, chromosome region‐specific 40‐Kb heat maps (8 Mb length), TAD score calculated with FAN‐C v0.9184 (Methods) for the same three species. The green square below the TAD score represents the gene at the corresponding position. Right, Schematic diagram of the Permutation test. The abscissa represents the number of chromosomes. The red dotted line represents the number of positively selected genes actually observed in the rearrangement region. The blue dot plot represents the number of positively selected genes in the sliding window simulation of each chromosome. (B) The distance from the chromosome rearrangement breakpoint region to the nearest TAD boundary in Ampullariidae species. If it is greater than 100 kb, it is considered to be located inside the TAD. The upper right is a statistical test of the distance between the breakpoint area of ​​above‐water and underwater spawning species from the TAD boundary. (C) Gene Ontology distributions of rearranged genes in *Pila celebensis* and *P. canaliculata* compared with AmLG and New‐world lineage ancestor (Chr2, 3, and 13), respectively.

To investigate the impact of chromosomal rearrangements on the behavioral patterns of Ampullariidae species, we reconstructed the ancestral linkage group of the Ampullariidae common ancestor (AmLG) and the last common ancestor of New‐World species and performed large‐scale synteny comparisons between extant species and their reconstructed ancestral linkage groups. Syntenic analyses were conducted between AmLG and *Pila celebensis*, as well as between New‐World lineage MLGs and *P. diffusa* or *M. cornuarietis* showed that chromosome numbers remained conserved across the entire family and its two ancestral nodes, despite the presence of significant inversions (Figure 2A; Figure S4). Specifically, (i) comparing *Pila celebensis* with AmLG revealed significant rearrangements on Chr 4, 6, 7, 8, 9, 10, and 11; (ii) in the New‐World lineages, *M. cornuarietis* exhibited rearrangements on Chr 3, 4, 6, 7 and 8 relative to the New‐World lineage MLGs, (iii) Chr2, 3, 4, 5, 12, 11, 6, 8 and 14 of *P. canaliculata* showed rearrangements compared to New‐World lineage MLG, while (iv) *P. diffusa* showed distinct rearrangements on Chr 2, 6, 8, 11, and 13 compared to New‐World lineage MLGs. To distinguish whether these structural variations are adaptive or merely neutral changes accumulated over time, we analyzed the selection pressure on genes within the rearranged blocks. We found that genes within rearranged regions exhibited significantly higher signals of positive selection compared to those in non‐rearranged regions (Table S7). Furthermore, a sliding‐window permutation test (using the number of rearranged genes as the window size and a one‐gene step) confirmed that the density of positively selected genes in most rearranged regions is far higher than in randomly sampled genomic regions (right in Figure 2A; Table S7; Figure S11). These results strongly support the hypothesis that chromosomal rearrangements in Ampullariids are adaptive drivers rather than neutral changes.

We further integrated Hi‐C data to examine the 3D regulatory landscape using topologically associating domains (TAD) analysis (center panel of Figure 2A). TAD scores were utilized to evaluate boundary confidence, where lower scores indicate more robust TAD boundaries. Our analysis revealed a striking divergence in the spatial distribution of rearrangement breakpoints: in aerial‐ovipositing species, breakpoints are significantly more likely to be sequestered within TAD interiors (defined as a distance >100 kb from the nearest TAD boundary; Figure 2B; Table S7). In contrast, breakpoints in aquatic‐ovipositing species tend to cluster at TAD boundaries. This suggests that while underwater species maintain ancestral regulatory constraints, aerial‐ovipositing lineages may have convergently reshaped their internal TAD architecture to facilitate novel gene regulatory modes essential for terrestrial adaptation. Finally, we compared the functional profiles of rearranged genes between *Pila celebensis* and *P. canaliculata* (with more comprehensive functional annotations than *P. diffusa*). Notably, Chr2, 3, and 13 underwent consistent rearrangements in both the *Pila* versus AmLG and *P. canaliculata* versus New‐World lineage MLGs comparisons, whereas no such rearrangements were observed between the aquatic‐ovipositing *M. cornuarietis* and New‐World lineage MLGs. Functional enrichment analysis (Figure 2C) revealed that genes within the shared rearrangement blocks on Chr 2, 3, and 13 are predominantly involved in metabolism, immune response, visual perception, and stress responses. These functions are strongly tied to the aerial oviposition process, further supporting the hypothesis that chromosomal rearrangements contributed to the adaptation in reproductive strategies between *Pila* and *Pomacea* [26, 40].

Given the availability of RNAseq data from multiple developmental stages and tissues of *P. canaliculata* [14], we further examined how gene expression and associated functions relate to the rearrangements observed in the comparison of *P. canaliculata* and New‐world lineage MLGs (Figure S12). Transcriptomic analysis showed elevated expression levels during testis development and early embryo stage (5–9 days), suggesting that chromosomal rearrangements are closely associated with early embryonic development and adaptations linked to aerial oviposition. Chromosomal rearrangements can mediate environmental adaptation by altering gene function in marine mammals, functional enrichment analysis of inversion regions between two whales contains two dominant functional categories (metabolism and stress response), which are linked to cetacean adaptation, metabolism, and development [41, 42]. In Gadid fishes, large‐scale chromosomal rearrangements underpin speciation and cold environment adaptation [43]. Similarly, genes affected by rearrangements in both *Pila* and *Pomacea*—relative to ancestral states—are consistently associated with aerial oviposition, underscoring a convergent genomic mechanism for this reproductive innovation. Although the genomic evidence (enrichment of rearranged genes and specialized expression) are consistent with an adaptive role for the rearrangements in this study, definitive demonstration will require targeted follow‐up work (for example, ATAC‐seq, ChIP‐seq, gene perturbation or biochemical characterization of the captured gene products). To evaluate the evolutionary specificity of chromosomal rearrangements within Ampullariidae, we compared the MLGs with the chromosome‐level genome of *Bellamya purificata* (Viviparidae). The *B. purificata* genome exhibits a highly condensed karyotype (2n = 16) compared to the conserved 2n = 28 found in most Ampullariids, reflecting extensive and chaotic structural divergence (Figure S13). While the current analysis is focused on the internal diversification of Ampullariidae, future studies incorporating a wider array of gastropod genomes will be essential to fully understand adaptive chromosomal evolution.

### 2.4 Gene Family Expansion and Selection Shaped the Genomic Parallelism in Aerial‐Ovipositing Ampullariidae

**FIGURE 3:**
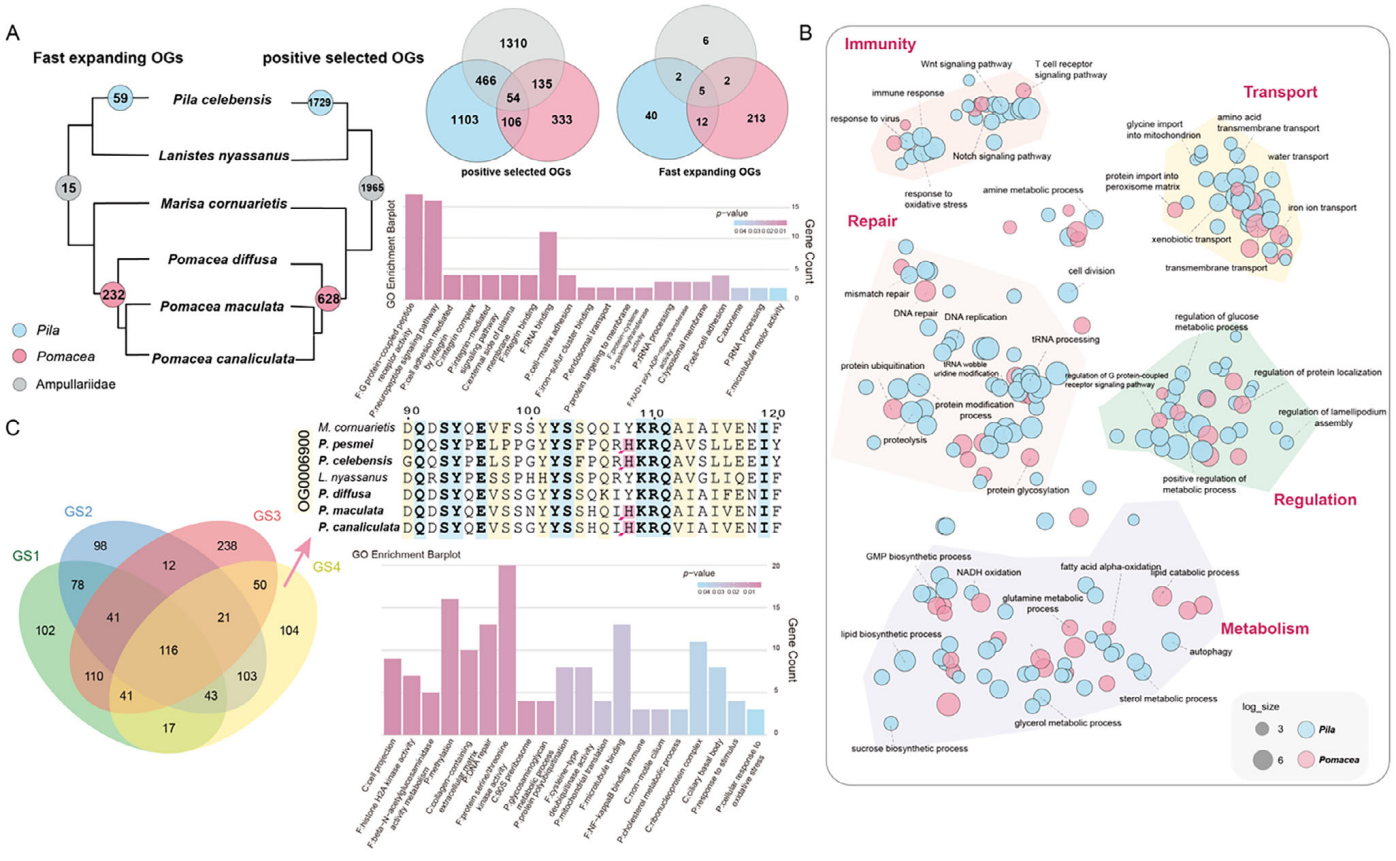
Functional characterization of parallel and exclusive orthogroup changes in *Pila* and *Pomacea* lineages. (A) Numbers in the colored circle indicate the count of fast‐expanding and positive selected orthogroups (OGs) across different lineages. Venn diagram on the right highlights the overlapping of fast‐expanding and positive selected OGs, while the Gene Ontology (GO) distributions illustrate all corresponding overlapped genes in *Pila* and *Pomace*. (B) Clustering based on GO enrichment results for uniquely fast‐expanding and positive selected genes in *Pila* and *Pomacea*. (C) Convergence analysis of the physicochemical properties of amino acids, categorized into four groups. The upper right section presents results for an immune‐related protein in GS4, while the lower right shows GO enrichment results for OGs exhibiting amino acid convergence.

Changes in gene family size serve as a primary driver of evolution [44]. In order to capture the full breadth of genomic changes in Ampullariidae facilitating the aerial oviposition, our analysis focused on the aerial ovipositors *Pila* and *Pomacea*. To pinpoint gene repertoire changes potentially associated with the shift in oviposition mode, we identified gene families that have rapidly expanded using BadiRate, or expanded using CAFÉ methods and positively selected in both lineages [45, 46, 47]. The CAFÉ analysis revealed 197 expanded gene families in Ampullariidae, 83 expanded gene families in *Pila* and 64 expanded gene families in *Pomacea* (Tables S8–S10). The BadiRate analysis detected 15 fast expanded gene families in Ampullariidae, 59 in *Pila* and 232 in the *Pomacea* (Figure 3A; Table S11). The branch‐site and site models detected a total of 1,965 positively selected gene families in Ampullariidae, with 1, 729 in *Pila celebensis* and 628 in *Pomacea* (Figure 3A; Tables S12–S15).

Consistent with previous findings in Sun et al. [14], genomic analyses of Ampullariidae reveal expansions in gene families associated with environmental sensing, metabolic adaptation, and immune regulation. As a discussion of each expanded gene family would be impossible, we here focus on discussing in more details of some representative genes in the above functions. For the metabolic adaptation, cellulase family 10 (GH10) and β‐D‐xylosidase exhibit significant expansion, which are key enzymes involved in the degradation of complex carbohydrates and catalyze the hydrolysis of cellulose (Tables S9 and S10; Figure S14) [48]. These kinds of enzymes could have facilitated the adaptation of environmental shift, which aligns with the omnivorous diet of Ampullariids including algae, plants, detritus, and zooplankton [20]. Aristide et al. also suggested that the expansion of glycosyl hydrolases could have facilitated the dietary shift associated with the habitat transition in mollusca [7]. In addition, immune‐related gene families also exhibit significant expansion, including C1q‐related, kelch‐like, and ficolin‐1‐like proteins. The expansion of the C1q family in Ampullariidae appears to be a complex process. While such expansions in molluscs are often driven by transposon‐mediated tandem duplications (Figure S15) [49], their retention suggest adaptive significance. Notably, the most highly expressed C1q variants in the albumen gland of New‐World species are located on chromosomes separate from the primary tandem clusters (e.g. in *P. canaliculata*, highly expressed C1q gene is located in Chr8 and tandem clusters are located in Chr14; Figure S15). This spatial decoupling suggests that specific copies may have undergone translocation, potentially enhancing resistance to environmental pathogens and supporting immune defense during embryogenesis via PVF evolution [26, 50]. Further research is required to fully elucidate the cis‐regulatory changes associated with these translocated copies.

### 2.5 Convergent Evolution of Gene Functions and Amino Acid Properties in Aerial‐Ovipositing Ampullariidae

Although only one expanded gene family in the CAFÉ results and 17 fast‐expanding families identified by BadiRate are shared between *Pila* and *Pomacea*, most expansions are lineage‐specific and primarily associated with environmental signal perception and regulation, immune defense, and energy metabolism (Figure 3B; Tables S9 and S10). These findings suggest that *Pila* and *Pomacea* have undergone convergent functional adaptations, utilizing similar biological pathways but different gene sets. Here, we focus on representative genes involved in environmental sensing, UV resistance, immunity, and desiccation resistance to explore their convergent adaptation mechanisms. For environmental sensing, the GPCR family, crucial for chemoreception in aquatic snails [14, 51], exhibits notable expansions in *Pila* (154 genes) and *Pomacea* (31 genes), far more than in other Ampullariids (3–5 genes). Phylogenetic analysis reveals *Pila* GPCRs cluster into large clades, while *Pomacea* GPCRs are more dispersed (Figure S16). Many GPCR genes in both genera are tandemly duplicated, with *Pila* showing more extensive tandem duplications (Figure S17).

In UV resistance, lineage‐specific expansions of UV‐related gene families were observed in both genera (Figure S18). *Pila* exhibits significant expansion of keratin genes (14 copies, compared to 1–5 in *Pomacea*), potentially enhancing cuticular UV protection [52]. In contrast, *Pomacea* shows expansion of the neprilysin gene family (9–13 copies, compared to 3 in *Pila*). While the function of neprilysin in molluscs remains unclear, its role in UV‐responsive pathways in mammals indicates a convergent mechanism [53]. Immunity also plays a key role in aerial oviposition. *Pila* shows significant expansions in the interleukin‐1 receptor (IL‐1R) and fucolectin‐3‐like gene families (Table S9). IL‐1R mediates immune and inflammatory responses to infections [54], while fucolectin‐3‐like genes are defensive agents in *Xenopus tropicalis* [55]. Conversely, *Pomacea* exhibits a marked expansion of cytochrome P450 (CYP) genes, essential for xenobiotic detoxification and stress hormone biosynthesis [56], aligning with prior reports linking CYP expansion to stress resistance and terrestrial adaptation in molluscs [7, 57]. Additionally, positively selected genes differ between the genera. Among the gene family under positive selection, we identified aquaporins (AQPs) as exhibiting parallel positive selection between *Pila* and *Pomacea* (Tables S12–S15). AQPs are transmembrane transport proteins that form channels facilitating water and small solute movement across membranes and play critical roles in osmoregulation [58]. Given the stark osmotic differences between aquatic and aerial environments, the adaptive evolution of AQPs likely supports the out‐of‐water oviposition strategy shared by both genera [2, 7]. Similarly, the gene families undergoing parallel positive selection in *Pila* and *Pomacea*, like the expanded gene families, are primarily involved in functions such as signal transduction, metabolism, and immunity. This parallel evolution facilitates their adaptation to the aerial oviposition process. Except the positively selected in AQPs family, genes linked to bioenergetic metabolism (e.g., hexokinase and TCA cycle genes) are also under positive selection in *Pila*, while in *Pomacea*, genes related to oxidative stress resistance, including CYP450 and flavin‐containing monooxygenase (FMO) families, show signs of adaptive evolution (Tables S14 and S15). These adaptations likely enhance resilience to environmental stresses.

To investigate convergent evolution beyond identical amino acid substitutions, we analyzed the Convergent Amino Acid Properties (CAAP) across *Pila* and *Pomacea*. Amino acids were categorized into four complementary schemes (GS1–GS4) to reflect different hierarchical levels of chemical and structural similarity, namely (i) GS1 is based on side chain polarity and charge; (ii) GS2 reflects hydrophobicity and functional similarity; (iii) GS3 is organized by chemical reactivity and structural roles; (iv) GS4 fine‐grained scheme uses individual physicochemical traits (detailed in methods of convergent evolution analysis of amino acid sites and Table S16). This method identifies sites where substitutions, though potentially different in identity, result in convergent physicochemical traits. In total, there are 548 OGs in GS1, 512 OGs in GS2, 629 OGs in GS3 and 495 OGs in GS4 that have AA sites with similar physical and chemical properties, and there are 116 OGs with convergent evolution of amino acid sites in all four classifications (Figure 3C; Table S16). To intuitively illustrate this approach, we provide a representative alignment of a single gene, demonstrating how divergent residues are unified by shared properties under the GS classifications (Figure 3C). Functional enrichment analysis showed that the genes located in these AA sites convergent OGs are primarily involved in metabolism, immune, regulation of organic compounds as well as response to oxidative stress and stimulus. This result further supports the convergent evolution of AA sites in *Pila* and *Pomacea*. The functions of these convergently evolved OGs are also closely related to the aerial‐ovipositing process of *Pila* and *Pomacea*. Overall, *Pila* and *Pomacea* exhibit functional convergence and convergent evolution of amino acid properties in high‐level biological processes associated with the transition to aerial oviposition, though these adaptations were achieved via distinct genomic pathways.

### 2.6 Egg Proteomes Show the Parallel Selection of PV1 Subunits in Aerial and Aquatic Eggs

**FIGURE 4:**
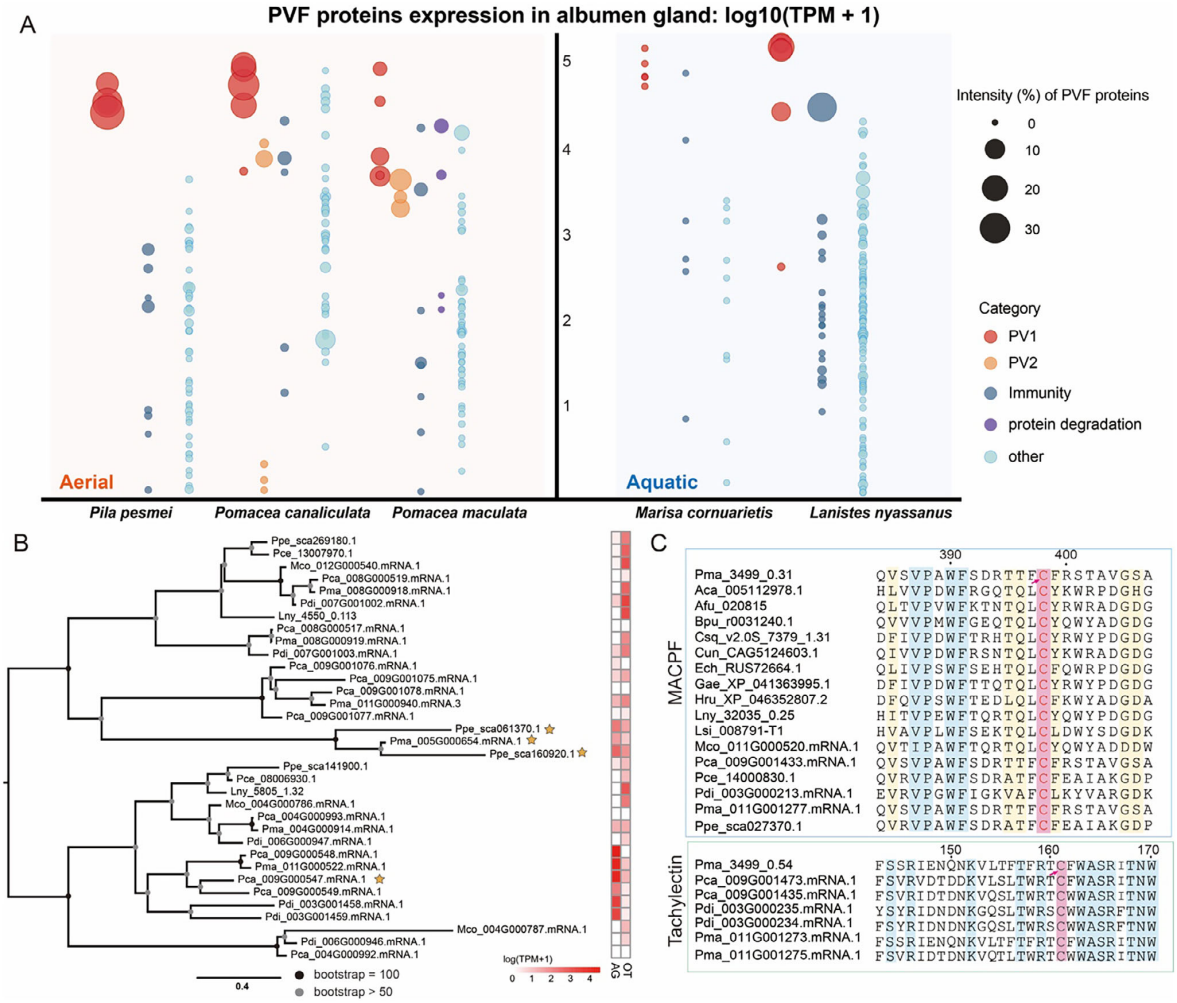
Profile of perivitelline fluid protein (PVF) in Ampullariidae. (A) Bubble plot of total PVF in five Ampullariidae species (*Pila celebensis*, *Pomacea canaliculata*, *Pomacea maculata*, *Lanistes nyassanus* and *Marisa cornuarietis*). The left side of the vertical axis represents three aerial egg laying species and the right side represents two underwater egg laying species. The color of the bubble represents the different categories to which the PVF protein belongs, the size represents the intensity of the protein in the egg mass, and the ordinate represents the TPM value of the transcriptome expression of the PVF protein. (B) Maximum‐likelihood phylogenetic tree of calcium binding proteins (CaBP) in Ampullariidae, with circles indicating bootstrap values. Sequences marked with yellow stars were detected in the protein MS data. Gene expression levels are presented on a logarithmic scale. AG: albumen gland; OT: other tissues. (C) Alignment results of MACPF‐like genes across all species used in Figure 1C and tachylectin‐like genes in Pomacea species. The pink background highlights the disulfide bond sites facilitating the binding and toxicity of MACPF and tachylectin.

Egg PVF proteins have been the primary focus in the water‐to‐land transition of Ampullariids over the last three decades [26, 40, 59, 60, 61], but there is a lack of PVF data in Old‐World species. To better understand the function and evolutionary history of PVF proteins, we generated Old‐World *Pila* and *Lanistes* PVF proteomes. LC‐MS/MS analysis identified 57 and 133 putative proteins in *Pila pesmei* and *Lanistes nyassanus* respectively, with 47 (82.5%) and 116 (87.2%) successfully annotated against the NCBI non‐redundant database (Table S17). Most of the PVF proteins are highly expressed in albumen gland while weakly expressed in the other tissues (Table S17). Based on the annotation of PVF proteins, PVF proteins of each species are mainly divided into several functional categories such as PV1, PV2, immunity, protein degradation, and antioxidant stress (Figure 4A). Strikingly, homologs of *Pomacea* PV1 constituted the predominant components of PVF proteomes across Ampullariids with higher relative abundance in aerial eggs (*Pila pesmei*: 90.79%; *P. canaliculata*: 84.72%; *P. maculata*: 87.0%) than these in aquatic eggs (*L. nyassanus*: 53.81%; *M. cornuarietis*: 73.2%). Transcriptomic profiling further corroborated these findings: PV1‐like genes showed AG‐specific overexpression (TPM > 500), while undetectable/weakly expressed in other tissues (Table S17). The tissue‐restricted expression pattern and PVF dominance of PV1‐like proteins strongly support their essential role in embryonic survival. Conversely, aquatic ovipositors exhibited enhanced immunological investment, with immune‐related proteins representing 29.76% (*L. nyassanus*) and 16.8% (*M. cornuarietis*) of PVF proteomes, versus only 2.12%–4.31% in aerial egg depositors (Table S17). The high abundance of immune proteins in aquatic snail eggs might reflect higher investment in immune related proteins for defense against pathogens. In addition, we observed the presence of calcium‐binding proteins in aerial eggs of *Pila* and *Pomacea*. Calcium binding protein (CaBP) was detected in the PVF protein MS of *Pila* and *Pomacea*, but there were differences in their expression levels (Figure 4B; Table S17). Although the TPM of CaBP in *Pila* is >380, in *Pomacea*, the TPM of CaBP is >10 000. Although this result may indicate parallel evolution of CaBP in aerial ovipositors and consistent with the hypothesis that aquatic eggs need more protection for their embryos under the threat of microbial infection [26, 59, 62], there are still certain differences in their evolution and expression patterns.

PV2 are novel neurotoxic PVF proteins exclusively identified in the Canaliculata clade (*P. canaliculata* and *P. maculata* [14, 24, 63]. This heterodimeric toxin consists of a MACPF subunit (PV2‐67, 67 kDa) and a tachylectin‐like subunit (PV2‐32, 31 kDa) [40]. Previous studies have detected a cysteine ​​site in MACPF (Cys398) and tachylectin (Cys161), which can interact to form a disulfide bond connecting the subunits to exert toxic functions. It is believed that members of the Canaliculata lineage produced a new toxic complex based on the function of the pre‐existing proteins for defense against predators [63, 64]. In this study, we reanalyzed the composition of PV2 and found that, while MACPF is conserved across most species in the species tree in Figure 1, the essential Cys binding site in tachylectin uniquely appears in the Canaliculata lineage and *P. diffusa* (Figure 4C). Notably, although *P. diffusa* possesses these Cys binding sites, its subunits exhibit minimal expression in the albumen gland (TPM < 1) and remain undetected in the PVF proteome (Table S17; Table S19). The absence of functional PV2 complexes in *P. diffusa* appears to be driven by a combination of regulatory decay and relaxed selective pressure. Specifically, in *P. diffusa*, the tachylectin gene is physically separated from its MACPF partner by multiple intervening duplicates, a rearrangement that contrasts with the proximal arrangement observed in toxic congeners such as *P. canaliculata* (Figure S22). Moreover, selective pressure analysis reveals a notable lack of positive selection (dN/dS) at the PV2 locus in *P. diffusa*, suggesting that the evolutionary maintenance of this toxin complex has been lost in this lineage (Figure S21). While these bioinformatic and omics‐level correlations strongly suggest regulatory failure, further experimental validation will be necessary to definitively characterize the specific mechanisms of PV2 loss in *P. diffusa*, as this protein may also have contributed to the high invasiveness of some members of the Canaliculata clade [65].

**FIGURE 5:**
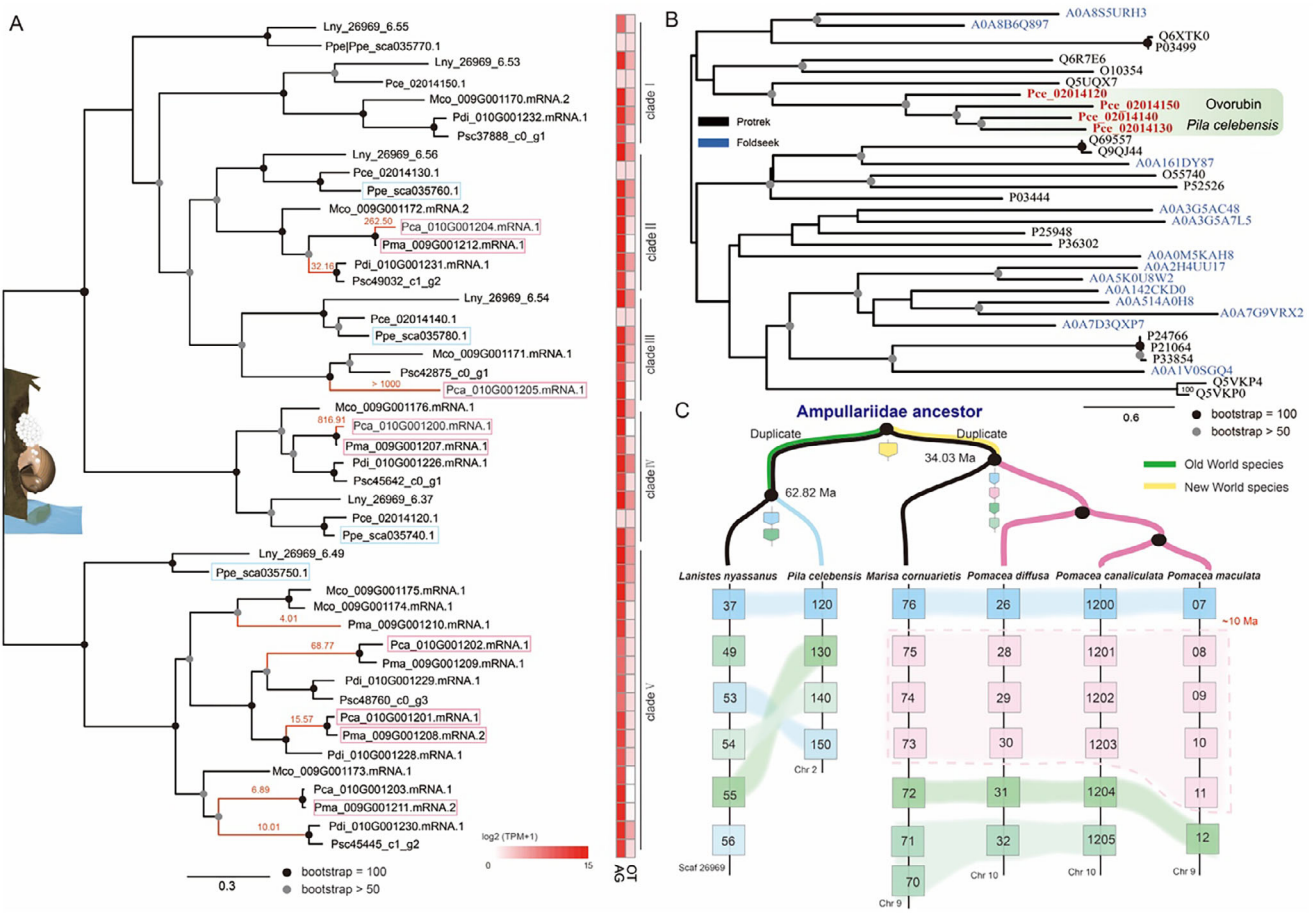
Phylogeny and evolution prediction of PV1 in Ampullariidae. (A) Phylogenetic tree and expression of PV1 homologues in Ampullariidae. Blue and pink frames indicate detection of proteins in the mass spectrometry (MS) dataset. Red branches signify positive selection, with dN/dS values shown in red. Gene expression levels are on a logarithmic scale. AG: albumen gland; OT: other tissues. (B) Phylogenetic analysis supporting the horizontal acquisition of PV1 from viruses. Sequences in red represent four PV1 sequences from *Pila celebensis*, blue sequences are the viral sequences obtained via Foldseek through 3D structure alignment, and the others are predicted using Protrek. (C) Schematic representation of PV1 evolution, with the yellow section indicating the common ancestor of PV1. Each node section indicates the ancestral number of PV1, and the pink section indicates a recent copy event occurring ∼10 million years ago (Ma).

PV1 is a major perivitellin that forms glyco‐lipoprotein complexes in PVF, and is uniquely present in the Ampullariidae family, with no presence in the other two architaenioglossan families, Cyclophoridae and Viviparidae. BLASTp analysis identified 46 PV1‐like genes across eight Ampullariidae species: six genes in four *Pomacea* species, seven in *Marisa cornuarietis*, six in *Lanistes nyassanus*, five in *Pila pesmei*, and four in *Pila celebensis*. Phylogenetic analysis of the PV1 genes revealed five major clades (Figure 5A). Most clades retained the phylogenetic relationship of the species tree, except for clade V, which exhibited New‐World specific gene duplications in *Pomacea* and *Marisa*. Sequence alignment and substitution analyses suggest that clades I–IV originated from ancestral duplications in Ampullariids, with divergence times aligning with species divergence (Figures S19 and S20). Clade V in New‐World lineages (*Marisa* and *Pomacea*) diverged around 10 million years ago, coinciding with the Andean uplift [33]. This temporal correlation suggests that the environmental shifts during this period provided the selective pressure necessary to favor the retention and adaptive radiation of gene duplicates, potentially facilitating rapid adaptation to new ecological niches.

Gene duplication provides new genetic material for selection [66], with clade V of *Pomacea* showing evidence of positive selection (Figure S20). Notably, positively selected sites revealed substitutions to aromatic residues, particularly phenylalanine (Phe). In aerial egg‐laying species, the Phe content in clade V of PV1 is approximately double that of aquatic egg‐laying species (Table S18), along with an increased hydrophobic‐to‐aromatic amino acid ratio. The approximately twofold increase in Phe residues in aerial‐laying species may promote stronger interactions with carotenoids, enhancing their sequestration and stabilization within the complex [67]. Such carotenoid–protein interactions could both reinforce complex stability and provide increased UV screening and antioxidant protection, traits that would be advantageous for embryos exposed to direct sunlight [24, 68]. Furthermore, aerial ovipositors in *Pomacea* (clade V) and *Pila* (clade V) show significant enrichment in hydrophobic residues. The substantial increase in hydrophobicity is predicted to stabilize the core of the PV1 lipoprotein complex, potentially producing a more cohesive, desiccation‐resistant molecular barrier that reduces water loss in arid conditions—an effect analogous to documented roles of hydrophobic protein domains in conferring desiccation resistance in insect eggs [18]. The parallel evolution of hydrophobic residues in PV1 suggests a shared adaptive mechanism for protecting aerially deposited eggs from desiccation in *Pomacea* and *Pila*.

To explore the origin of PV1, we conducted a multi‐faceted analysis integrating compositional, structural, and phylogenetic evidence. Compositional analysis revealed that both the total GC content and the GC content at the third codon position (GC3) of PV1 across Ampullariidae are significantly higher than the genomic average (Table S20). This distinct nucleotide bias serves as a classic signature of exogenous gene integration. Furthermore, we expanded our structural homology search using Protrek and FoldSeek. Analysis of PV1 sequences from both *P. canaliculata* and *Pila celebensis* identified high similarity scores (20–35) and viral‐like protein folds in the SwissProt and UniRef50 databases, providing robust evidence for a viral origin. To verify this, we reconstructed a comprehensive phylogeny including PV1 and its top viral homologs. The resulting topology shows that the Ampullariidae PV1 clade is deeply nested within a cluster of viral sequences (Figure 5B; Figures S23–S25; Table S21). Importantly, our search across diverse molluscan lineages confirmed that PV1 is strictly confined to Ampullariidae and absent in the sister family Viviparidae and other gastropods. This supports the hypothesis that PV1 was acquired via ancient horizontal gene transfer (HGT) from viruses in the Ampullariidae ancestor, likely during the Jurassic period (Figure 1B). Advances in genomics have increasingly uncovered HGT in molluscs, with previous reports documenting viral‐derived HGT in the sea slug *Elysia crispata* and scallop *Chlamys farreri* [69, 70]. Our study provides a notable example of viral HGT in molluscan lineages that has functional consequences.

Genomic analysis revealed that PV1 genes are organized in tandem arrays on the same chromosome in six Ampullariidae species (Figure 5C). Based on divergence time estimates (Figures S19 and S20) and HGT evidence, we propose an evolutionary model for PV1 in Ampullariidae. The ancestral PV1, acquired via viral HGT (yellow rectangle in Figure 5C), underwent successive duplication and divergence. In Old‐World lineages, duplication occurred prior to the *Pila*‐*Lanistes* split, resulting in six copies in *L. nyassanus* and four in *Pila celebensis*. In New‐World lineages, an ancestral tandem array of four copies was established; subsequently, approximately 10 million years ago, a further duplication event in the Canaliculata clade led to seven copies in *M. cornuarietis* and six in *Pomacea* species. In summary, the viral acquisition and subsequent duplication of PV1 facilitated the functional diversification of egg‐laying strategies within Ampullariidae. In summary, the acquisition of PV1 via viral HGT in the ancestor of Ampullariidae established a unique genetic foundation for the family. Subsequent lineage‐specific gene duplications led to an expanded PV1 repertoire; notably, the low sequence similarity (∼30%; Table S22) among these duplicated subunits maybe provided the genetic flexibility necessary for members of Ampullariidae to explore and refine diverse reproductive strategies.

## 3 Conclusion

This study provides the first two assembled genomes for Old‐Wolrd *Pila*, offering a pivotal resource for dissecting the genomic basis of convergent evolution. We demonstrate that the independent origins of aerial oviposition in *Pila* and *Pomacea* were driven by multi‐layered genomic parallelism and convergence. Parallel evolutionary mechanisms are evident in both functional dimensions, we identified overlapping expansions in gene families such as cellulases, beta‐D‐xylosidases, and C1q domains, which likely coordinate to support the physiological demands of terrestrial reproduction. Proteomic profiling of the PVF demonstrates a parallel increase in the proportion of the PV1 protein in aerial eggs compared to aquatic ones, underscoring its pivotal role in the transition to land. Although PV1 originated from an ancient viral horizontal gene transfer, its subsequent evolution in aerial lineages exhibit parallel increases in hydrophobicity and a doubling of aromatic Phe residues. Simultaneously, we observe striking adaptative convergence where different genomic routes lead to similar adaptive outcomes. While both lineages underwent chromosomal rearrangements, the breakpoints in aerial‐laying species show a convergent tendency to be located within TADs, potentially rewiring regulatory landscapes for desiccation tolerance. Gene family analyses reveal convergent positive selection in AQPs for osmoregulation and distinct pathways converging on UV radiation resistance. This convergence extends to the molecular level, characterized by convergent shifts in amino acid physicochemical properties across 116 orthogroups. Collectively, our findings demonstrate that major innovations emerge through a “Two Routes” narrative: shared gene repertoire and molecular feature of PVF, coupled with convergent refinements in regulatory architecture, function similar and protein composition. By integrating these diverse genomic and proteomic layers, this study provides a comprehensive methodological framework for investigating the mechanisms underpinning major ecological transitions. Our approach serves as a template for future research on other complex evolutionary shifts—such as freshwater colonization, dietary transitions, or land invasions—across diverse taxa.

## 4 Methods

### 4.1 Sample Collection

We collected *Pila celebensis* specimens from Khanun District, Patthalung Province, Thailand (7°44′6″N, 100°0′36″E), in 2019. We dissected one female *Pila celebensis* to obtain foot tissue for genome sequencing and dissected three individual females to various tissues (albumen gland, foot, mantle, and digestive gland) for transcriptome sequencing as three individual biological replicates. We collected *Pila pesmei* individuals from Nong Phok District, Roi‐et Province, Thailand (16°18′35″N, 104°12′11″E), in 2012. From one female *Pila pesmei*, we collected foot tissue for genome sequencing and two egg masses for proteomic analysis. We also sampled two mass eggs from *Lanistes nyassanus* (snail source same as in Sun et al., [14] as representatives of the Old‐World underwater lineage for comparison with the *Pila* PVF. All samples were stored at −80°C before downstream analysis. We confirmed the identity of both *Pila* species by DNA barcoding of the cytochrome *c* oxidase subunit I (COI) gene. The methods of genome sequencing, genome annotation and phylogeny are in the supplementary information.

### 4.2 Synteny Analysis

Synteny and MLG fusion analyses were limited to the chromosome‐level *Pila celebensis* assembly, as the scaffold‐level *Pila pesmei* assembly lacks Hi‐C anchoring required for accurate detection of large‐scale chromosomal rearrangements. Protein sequences of *Pila celebensis* and four New‐World Ampullariids with the gene coordinate files (.*bed* format) were used for the ancestor linkage groups and inter‐species synteny analysis compared with MLGs [37]. The ancient linkage groups of ancestors of Ampullariidae and Viviparidae, Ampullariidae (AmLGs) and *Pomacea* ancestors were identified according to methods of Schultz et al. [71]. In brief, Diamond BLASTp v2.1.0 was conducted against corresponding species under each ancestor node, obtaining reciprocal best hits among them. Then orthologs were grouped together based on whether they exist on the same set of scaffolds in each species. After removing the groups of orthologues with a less‐than‐significant false discovery rate (0.05), the remaining groups, which presented on the same set of chromosomes, were considered as a common ancestor of these species. Syntenic analysis between Ampullariids and ancestor linkage groups were identified through reciprocal BLAST searches, and the linkage groups were further checked Fisher's exact test using R package macrosyntR with a significant threshold of *p*‐value < 0.01 [72]. The inter‐chromosomal rearrangements were visualized using Oxford‐plot (https://bitbucket.org/viemet/public/src/master/CLG/). The expression levels of genes rearranged in chromosomes in different *P. canaliculata* tissues were displayed through a heat map, and the data used was PRJNA473253 published in Sun et al. [14].

In addition, self‐BLASTp was conducted to identify the paralogous genes with a threshold of E‐value of 1e^−5^. MAFFT and KaKs_Calculator v3.0 were used to align sequences and calculate the number of synonymous substitutions per synonymous sites (Ks) values, respectively [73]. The Ks distribution was plotted using ggplot package from R v4.3.1 with “density” function.

### 4.3 Rearranged Genes Expression of *P. canaliculata*

To evaluate the transcriptional profiles of the identified rearranged genes in *P. canaliculata*, we leveraged publicly available transcriptomic data. Due to limitations in current transcriptomic sampling, RNA‐seq raw reads were retrieved from the study by Sun et al., [14]. These reads were then quantified against the updated reference genome of *P. canaliculata* provided by Xiong et al. using Salmon v1.10.3 to obtain transcript abundance estimates [25, 74]. The resulting expression levels were normalized as Transcripts Per Million (TPM). Finally, a heatmap illustrating the expression patterns of these rearranged genes across different samples was generated using the ggplot2 in R.

### 4.4 Hi‐C Data Processing and TAD Identifications

Quality control and data preprocessing. Hi‐C data from the Ampullariidae species were first trimmed adapter sequences and low‐quality reads with Trimmomatic (v.0.36) [75]. Hic‐Pro (v2.9.0) was then used to process the Hi‐C data from raw sequencing data via a pipeline including alignment, matrix construction, matrix balancing, and iterative correction and eigenvector decomposition normalization (ICE) with the default parameters [76]. Then we identified TADs as follows. We first implemented the conversion of Hi‐C matrices to Cooler format via HiCPeaks (v0.3.2) [77]. To detect the TADs, we then calculated the directionality index (DI) with a resolution of a Hi‐C matrix at 40 kb using the hitad function from TADLib (v0.4.2) [78]. TADbit v1.0 was employed to calculate TAD boundary strength, assigning values from 1 to 10 (referred to as ‘TADbit score’), as it has proven to be more accurate in defining TADs on invertebrate species [79]. Plots displaying contact heat maps, along with insulator score tracks and specific genes, were generated using the pyGenomeTracks tool in HiCExplorer v 3.7 [80]. In total, we obtained 2401 TADs of *Pila celebensis*, 1153 TADs of *P. canaliculata*, 1151 TADs of *P. maculata*, 1391 TADs of *P. diffusa* and 1484 TADs of *M. cornuarietis* for subsequent analysis.

### 4.5 Permutation Test

To statistically evaluate whether chromosomal rearrangement blocks were significantly enriched with genes under positive selection, we implemented a permutation test based on a genome‐wide sliding window approach. For each identified rearrangement block containing *n* genes, we first determined the observed number of positive selection genes (O) located within the block. To generate a null distribution, we employed a sliding window of size n with a step size of one gene across the entire chromosome‐level genome assembly, calculating the count of positive selection genes in every possible genomic window of equivalent size. The expected mean and standard deviation were derived from this empirical distribution to calculate a Z‐score (Z = (O−mean)/SD), while the empirical *p*‐value was defined as the proportion of windows with a gene count greater than or equal to the observed value. To account for multiple testing, all *P*‐values across the analyzed blocks were corrected using the Benjamini‐Hochberg (BH) procedure, where an adjusted *p*‐value of less than 0.05 was considered statistically significant. These computational analyses and the resulting visualizations were performed in the R environment using the ggplot2 package. The R script of Permutation test is placed on figshare via 10.6084/m9.figshare.30479291.

### 4.6 Hox Gene Analysis

Hox gene clusters were identified using BLASTp searches against previously published Ampullariid genomes and other molluscan genomes, with manual verification of the Hox domain against Pfam 33.1 [14, 81].

### 4.7 Insertion Times of Transposable Elements

To reveal the dynamics of transposable elements (TEs) in Ampullariids, repetitive elements of six Ampullariids were annotated with RepeatModeler2 and RepeatMasker as described in the genome annotation section (details in Table S4). TEs and repeat landscape of Kimura substitution rate (K) were extracted from RepeatMasker results using “calcDivergenceFromAlign.pl” and “createRepeateLandscape.pl” scripts, respectively [82]. Insertion time of TEs was estimated using the equation “T = K/2r,” where T is the insertion time, and r is the nucleotide substitution rate for Ampullariids [83]. The substitution rate of Ampullariids was estimated according to Ip et al. using a free‐ratio model in the codmel script implemented in PAML v4.9 [84, 85].

### 4.8 Gene Family Analysis

The orthofinder results were used for gene family analysis using CAFE5 v5.0.0 [46]. Significantly expanded or contracted gene families were identified with a threshold of “family‐level *p*‐value” < 0.05. For the fast‐expanding gene family analysis, we reconstructed fast‐expanding gene family using BadiRate v1.3.5 as in Pau Balart‐García et al. [86]. GO and KEGG enrichment analyses were conducted for expanded gene families in *Pomacea* and *Pila* using clusterProfiler R package [87]. To further reveal the evolution of selected gene families, maximum likelihood trees were reconstructed using IQ‐TREE with settings detailed in the phylogenomic part in supplementary information.

Positive selection analysis was performed on the 7788 orthologous genes identified using PhyloPyPruner, and only OGs that included at least one species in *Pomacea* species or *Pila celebensis* were used in the downstream analysis [88]. Alignment of the amino acid sequences of 7788 OGs was conducted with MAFFT, and the nucleotide sequences of these genes were aligned according to the amino acid alignment using Pal2nal [89, 90]. HYPHY v2.5.61 was used to predict positively selected sites using Mixed Effects Model of Evolution model (MEME) and the adaptive Branch‐Site Random Effects Likelihood model (aBSREL) [47]. The foreground branches (*Pomacea* species and *Pila celebensis*) were assigned in aBSREL model, respectively. The nonsynonymous/synonymous substitution ratio (*ω* = dN/dS) and *p*‐value were used to detect whether a gene was subject to adaptive evolution, with *ω* > 1 and *p*‐value < 0.05 indicating that a gene was under positive selection. For genes in rearranged regions, follow the above method to detect positive selection.

### 4.9 Convergent Evolution Analysis of Amino Acid Sites

To detect convergent evolution analysis in amino acid sites, mainly referring to the method of Chen et al., amino acids are divided into four categories according to their physical and chemical properties to test whether there is convergent evolution of amino acid sites in gene families [91]. To ensure that identified convergent orthologous groups (OGs) were not inflated by neutral or conservative changes, we implemented a rigorous filtering and statistical framework. Amino acid substitutions were categorized as either conservative (occurring within the same physicochemical group) or radical (crossing group boundaries), with the former excluded as neutral noise due to their functional interchangeability. The statistical significance of the remaining radical changes was then assessed using the CAAP method, which utilized a null model based on the LG amino acid substitution matrix and site‐specific evolutionary rates to estimate the expected frequency of random radical convergence (Epc). Assuming that neutral convergent events follow a Poisson distribution with a mean of Epc, we calculated a *p*‐value for each OG to determine the probability of observing the actual count of radical events (Opc) by chance. Only OGs demonstrating a significant excess of convergence—defined by a convergence ratio R = O pc/Epc>1 and *p* < 0.05—were retained, resulting in a high‐confidence set of 116 OGs for subsequent analysis. GO and KEGG enrichment analyses of positively selected genes in the *Pomacea* branch and *Pila* branch were conducted using clusterProfilter R package.

### 4.10 Analysis of PV1 and PV2

The detailed methods of PVF extraction and LC‐MS/MS analysis are included in the SI. To further study the evolution of PV1 and PV2 genes, we conducted the BLASTp search among the nine Ampullariids (*Pila celebensis, Pila pesmei, Lanistes nyassanus, M. cornuarietis, P. canaliculata, P. maculata, P. diffusa*, and *P. scalaris* (transcriptome only)) and 16 other molluscan species genomes used in the phylogenetic tree. We also sequenced and *de novo* assembled two architaenioglossans *Cyclophorus subcarinatus* (23 873 unigenes; Cyclophoridae) and *Sinotaia aeruginosa* (23 620 unigenes; Viviparidae) using Trinity for PVF analysis. The threshold for BLASTp was set with an *E*‐value of 1e^−5^, pairs aligned length ≥ 30%, and aligned sequence identity ≥ 30%.

Phylogenetic trees of PV1‐like, MACPF‐like and tachylectin‐like genes were constructed using amino acid sequences, which were aligned using L‐INS‐I methods with MAFFT v7.525 and trimmed with BMGE v1.12 [89, 92]. IQ‐TREE was used to reconstruct a phylogenetic tree using the maximum‐likelihood method and the model of MFP with a bootstrap value of 1000. The RNAseq data (SRR6395538, SRR6395540, SRR6394739, SRR6394740, SRR6395701, SRR6395700, SRR6395705, SRR6395706, SRR6395702, SRR6394741, SRR6394742, SRR6394704, SRR6394703, SRR6395720 and SRR6395719) were mapped to the 6 genomes and 1 transcriptome for determination of gene expression level expressed as Transcripts Per Kilobase Million (TPM) using Salmon v1.8.0 [74].

Selective pressure was determined by the ratio (*ω* = dN/dS) with purifying, neutral, or positive selection indicated by *ω* < 1, = 1, or > 1, respectively. Substitution rate of each branch was estimated using the HYPHY to perform an exploratory analysis with the aBSREL and MEME model, respectively (similar to the above section). PV1‐like, MACPF‐like and tachylectin‐like genes were used to infer the divergence time, respectively. The divergence times were estimated with BEAST v2.6.5 [93]. An XML (Extensible Markup Language) file was generated with BEAUti (version 2, included in the BEAST package). The three phylogenetic trees obtained from the previous step were fixed by removing Wide‐exchange, Narrow‐exchange, Wilson‐Balding and Subtree‐slide. The rates of evolutionary changes at nuclear sites were estimated using ModelTest v3.7 with the GTR substitution model [94]. Divergence time and corresponding CIs were conducted with a log‐normal relaxed molecular clock and the Yule speciation prior. Three fossil time points, that is, a hard upper bound of 150 MYA for the split of *L. nyassanus* for PV1‐like genes, a hard lower bound of 470.2 MYA and a soft upper bound of 531.5 MYA for MACPF‐like genes and a hard minimum 390 MYA bound for tachylectin‐like genes, were selected for calibration. After 10 000 000 generations, the first 10% were removed as burn‐in. The log file was checked for convergence with Tracer v1.52 [95]. Consequently, a maximum clade credibility (MCC) tree was summarized with TreeAnnotator (version 2.6.5, included in the BEAST package), annotating clades with > 0.8 posterior probability (PP).

In addition, dN approach was used to further estimate the gene duplication time in each species. ParaAT was used to align protein sequences with gene‐coding sequences. KaKs_Calculator 3.0 was used to calculate the dN/dS ratio in all aligned pairs, and the dN value between paralogous gene pairs was determined using the MYN (Modified YN) model [96]. Divergence time was inferred using the formula “T = dN/2R,” where R is 2.67E‐8 synonymous substitutions per site per year.

We searched the PV1 for possible HGT using the AI matching score and 3D structure prediction as previously described [97, 98]. After obtaining 41 PV1‐associated sequences predicted by Protrek and FoldSeek, we aligned these sequences using MAFFT and IQ‐TREE was used to reconstruct a phylogenetic tree using the maximum‐likelihood method and the model of MFP with a bootstrap value of 1000.

### 4.11 Statistical Analysis

Significant chromosomal linkages, the linkage groups between every two genomes, and the spatial association between rearrangement breakpoints and TAD boundaries were all assessed using Fisher's exact tests (two‐sided). Specifically, a threshold of *p* < 0.01 was applied for synteny analysis, while *p* < 0.05 (or FDR < 0.05) was used to define significant ancestral clusters and breakpoint‐TAD proximity. To evaluate the enrichment of positively selected genes within rearrangement blocks, an empirical permutation test based on a genome‐wide sliding window approach (step size = 1 gene) was implemented. Evolutionary selection was identified using the MEME and aBSREL models in HYPHY, where genes with a likelihood ratio test *p*‐value < 0.05 and an ratio (dN/dS) > 1 were considered under positive selection. To control for multiple comparisons, *P*‐values derived from permutation tests, gene family expansions (CAFE5), and GO/KEGG enrichment analyses were adjusted using the Benjamini‐Hochberg (BH) procedure, where an adjusted *p*‐value < 0.05 was considered statistically significant. All computational analyses and visualizations were performed in the R environment (v4.3.1) using packages including macrosyntR, clusterProfiler, and ggplot2.

## Conflicts of Interest

The authors declare no competing interests.

## Supporting information




**Supporting File 1**: advs75951‐sup‐0001‐Figure S1‐S30.doc.

**Supporting File 2**: advs75951‐sup‐0001‐Table S1‐S17.xlsx.

## Data Availability

The raw sequencing datasets for *Pila celebensis* and *Pila pesmei* have been deposited in the NCBI SRA database under BioProject accessions PRJNA1355022 and the mass spectrometry proteomics data have been deposited to the ProteomeXchange Consortium via the PRIDE partner repository with the dataset identifier PXD070092. Genome assemblies, annotations of two Ampullariid species can be accessed via 10.6084/m9.figshare.30479291.

## References

[1] C. Román‐Palacios , D. Moraga‐López , and J. J. Wiens , “The Origins of Global Biodiversity on Land, Sea and Freshwater,” Ecology Letters 25 (2022): 1376–1386, 10.1111/ele.13999. 35334149

[2] G. I. Martínez‐Redondo , C. Simón Guerrero , L. Aristide , P. Balart‐García , V. Tonzo , and R. Fernández , “Parallel Duplication and Loss of Aquaporin‐Coding Genes during the “out of the Sea” Transition as Potential Key Drivers of Animal Terrestrialization,” Molecular Ecology 32 (2023): 2022–2040. 36652554 10.1111/mec.16854

[3] F. Falcon , E. M. Tanaka , and D. Rodriguez‐Terrones , “Transposon Waves at the Water‐to‐Land Transition,” Current Opinion in Genetics & Development 81 (2023): 102059, 10.1016/j.gde.2023.102059. 37343338

[4] A. Meyer , S. Schloissnig , P. Franchini , et al., “Giant Lungfish Genome Elucidates the Conquest of Land by Vertebrates,” Nature 590 (2021): 284–289, 10.1038/s41586-021-03198-8. 33461212 PMC7875771

[5] K. Wang , J. Wang , C. Zhu , et al., “African Lungfish Genome Sheds Light on the Vertebrate Water‐to‐Land Transition,” Cell 184 (2021): 1362–1376.e18, 10.1016/j.cell.2021.01.047. 33545087

[6] C. Vargas‐Chávez , L. Benítez‐Álvarez , G. I. Martínez‐Redondo , et al., “An Episodic Burst of Massive Genomic Rearrangements and the Origin of Non‐Marine Annelids,” Nature Ecology & Evolution 9 (2025): 1263–1279, 10.1038/s41559-025-02728-1. 40533512

[7] L. Aristide and R. Fernández , “Genomic Insights into Mollusk Terrestrialization: Parallel and Convergent Gene family Expansions as Key Facilitators in Out‐of‐the‐Sea Transitions,” Genome Biology and Evolution 15 (2023): evad176, 10.1093/gbe/evad176. 37793176 PMC10581543

[8] P. J. Krug , S. A. Caplins , K. Algoso , et al., “Phylogenomic Resolution of the Root of Panpulmonata, a Hyperdiverse Radiation of Gastropods: New Insight Into the Evolution of Air Breathing,” Proceedings of the Royal Society B: Biological Sciences 289 (2022): 20211855, 10.1098/rspb.2021.1855. PMC8984808 35382597

[9] G. J. Vermeij and V. M. Watson‐Zink , “Terrestrialization in Gastropods: Lineages, Ecological Constraints and Comparisons With Other Animals,” Biological Journal of the Linnean Society 136 (2022): 393–404, 10.1093/biolinnean/blac053.

[10] A. E. Staikou , “Shell Temperature, Activity and Resistance to Desiccation in the Polymorphic Land Snail *Cepaea Vindobonensis* ,” Journal of Molluscan Studies 65 (1999): 171–184, 10.1093/mollus/65.2.171.

[11] Y. Yom‐Tov , “Body Temperature and Light Reflectance in Two Desert Snails,” Journal of Molluscan Studies 39 (1971): 319–326, 10.1093/oxfordjournals.mollus.a065111.

[12] T. Knigge , M. A. Di Lellis , T. Monsinjon , and H.‐R. Köhler , “Relevance of Body Size and Shell Colouration for Thermal Absorption and Heat Loss in White Garden Snails, *Theba Pisana* (Helicidae), from Northern France,” Journal of Thermal Biology 69 (2017): 54–63, 10.1016/j.jtherbio.2017.06.001. 29037405

[13] M. Schweizer , R. Triebskorn , and H. R. Köhler , “Snails in the Sun: Strategies of Terrestrial Gastropods to Cope with Hot and Dry Conditions,” Ecology and Evolution 9 (2019): 12940–12960, 10.1002/ece3.5607. 31788227 PMC6875674

[14] J. Sun , H. Mu , J. C. H. Ip , et al., “Signatures of Divergence, Invasiveness, and Terrestrialization Revealed by Four Apple Snail Genomes,” Molecular Biology and Evolution 36 (2019): 1507–1520, 10.1093/molbev/msz084. 30980073 PMC6573481

[15] K. A. Hayes , R. H. Cowie , A. Jørgensen , R. Schultheiß , C. Albrecht , and S. C. Thiengo , “Molluscan Models in Evolutionary Biology: Apple Snails (Gastropoda: Ampullariidae) as a System for Addressing Fundamental Questions*,” American Malacological Bulletin 27 (2009): 47–58, 10.4003/006.027.0204.

[16] T. R. Brola , M. Y. Pasquevich , M. L. Giglio , et al., “Evolution of Aquatic Snails′ Defences Resulted in Clade‐Specific Differences in Egg Toxicity, Pigments and Warning Coloration,” Proceedings of the Royal Society B: Biological Sciences 293 (2026): 20251792, 10.1098/rspb.2025.1792. 41667099

[17] G. Barker , “Gastropods on Land: Phylogeny, Diversity and Adaptive Morphology,” in The Biology of Terrestrial Molluscs, Edited by G. Barker , 2001, Wallingford: CAB International, ISBN 9780851993188.0001.

[18] H. C. Vargas , K. A. Panfilio , D. Roelofs , and G. L. Rezende , “Increase in Egg Resistance to Desiccation in Springtails Correlates with Blastodermal Cuticle Formation: Eco‐Evolutionary Implications for Insect Terrestrialization,” Journal of Experimental Zoology Part B: Molecular and Developmental Evolution 336 (2021): 606–619, 10.1002/jez.b.22979. 32649025

[19] R. H. Cowie , “The Recent Apple Snails of Africa and Asia (Mollusca: Gastropoda: Ampullariidae: *Afropomus, Forbesopomus, Lanistes, Pila, Saulea*): a Nomenclatural and Type Catalogue. The Apple Snails of the Americas: Addenda and Corrigenda,” Zootaxa 3940 (2015): 1–92, 10.11646/zootaxa.3940.1.1. 25947491

[20] K. A. Hayes , R. L. Burks , A. Castro‐Vazquez , et al., “Insights From an Integrated View of the Biology of Apple Snails (Caenogastropoda: Ampullariidae),” Malacologia 58 (2015): 245–302, 10.4002/040.058.0209.

[21] S. Annate , Comparative Study on Reproductive Behavior of Three Thai Indigenous Apple Snails *Pila Celebensis* (Quoy & Gaimard, 1834), *P. pesmei* (Morlet, 1889) and *P. virescens* (Deshayes, 1824) (Chula Digital Collections, 2024), 176, 10.58837/CHULA.THE.2024.325.

[22] K. A. Hayes , R. H. Cowie , and S. C. Thiengo , “A Global Phylogeny of Apple Snails: Gondwanan Origin, Generic Relationships, and the Influence of Outgroup Choice (Caenogastropoda: Ampullariidae),” Biological Journal of the Linnean Society 98 (2009): 61–76, 10.1111/j.1095-8312.2009.01246.x.

[23] P. A. Selden , “Terrestrialization (Precambrian–Devonian),” in Encyclopedia of Life Sciences, 2001, Edited by John Wiley & Sons, Ltd., Hoboken, NJ.

[24] H. Heras , M. V. Frassa , P. E. Fernandez , C. M. Galosi , E. J. Gimeno , and M. S. Dreon , “First Egg Protein With a Neurotoxic Effect on Mice,” Toxicon 52 (2008): 481–488, 10.1016/j.toxicon.2008.06.022. 18640143

[25] J. Xiong , Y. Gao , Y. Zhou , et al., “Four Chromosome‐Scale Ampullariid Genomes: High‐quality Resources for Ecological, Evolutionary, and Invasion Biology Studies,” DNA Research 32 (2025): dsaf010, 10.1093/dnares/dsaf010. 40344091 PMC12131234

[26] J. C. H. Ip , H. Mu , Y. Zhang , et al., “Understanding the Transition From Water to Land: Insights From Multi‐Omic Analyses of the Perivitelline Fluid of Apple Snail Eggs,” Journal of Proteomics 194 (2019): 79–88, 10.1016/j.jprot.2018.12.014. 30557667

[27] D. Roelofs , A. Zwaenepoel , T. Sistermans , et al., “Multi‐faceted Analysis Provides Little Evidence for Recurrent Whole‐genome Duplications during Hexapod Evolution,” BMC Biology 18 (2020): 1–13, 10.1186/s12915-020-00789-1. 32460826 PMC7251882

[28] T. M. Linscott , A. González‐González , T. Hirano , and C. E. Parent , “De Novo Genome Assembly and Genome Skims Reveal LTRs Dominate the Genome of a Limestone Endemic Mountainsnail (*Oreohelix idahoensis*),” BMC Genomics 23 (2022): 796, 10.1186/s12864-022-09000-x. 36460988 PMC9719178

[29] Z. Chen , Ö. Doğan , N. Guiglielmoni , A. Guichard , and M. Schrödl , “Pulmonate Slug Evolution Is Reflected in the De Novo Genome of *Arion vulgaris* Moquin‐Tandon, 1855,” Scientific Reports 12 (2022): 14226, 10.1038/s41598-022-18099-7. 35987814 PMC9392753

[30] M. Wang , J. Li , P. Wang , et al., “Comparative Genome Analyses Highlight Transposon‐mediated Genome Expansion and the Evolutionary Architecture of 3D Genomic Folding in Cotton,” Molecular Biology and Evolution 38 (2021): 3621–3636, 10.1093/molbev/msab128. 33973633 PMC8382922

[31] W. Jokat , T. Boebel , M. König , and U. Meyer , “Timing and Geometry of Early Gondwana Breakup,” Journal of Geophysical Research: Solid Earth 108 (2003): 001802, 10.1029/2002JB001802.

[32] M. Sil , N. Aravind , and K. P. Karanth , “Into‐India or Out‐of‐India? Historical Biogeography of the Freshwater Gastropod Genus *Pila* (Caenogastropoda: Ampullariidae),” Biological Journal of the Linnean Society 129 (2020): 752–764, 10.1093/biolinnean/blz171.

[33] C. Hoorn , F. P. Wesselingh , H. ter Steege , et al., “Amazonia Through Time: Andean Uplift, Climate Change, Landscape Evolution, and Biodiversity,” Science 330 (2010): 927–931, 10.1126/science.1194585. 21071659

[34] F. Wesselingh , M. Räsänen , G. Irion , et al., “Lake Pebas: a Palaeoecological Reconstruction of a Miocene, Long‐lived Lake Complex in Western Amazonia,” Cainozoic Research 1 (2001): 35–68.

[35] K. Matsukura , M. Okuda , N. J. Cazzaniga , and T. Wada , “Genetic Exchange Between Two Freshwater Apple Snails, *Pomacea Canaliculata* and *Pomacea Maculata* Invading East and Southeast Asia,” Biological Invasions 15 (2013): 2039–2048, 10.1007/s10530-013-0431-1.

[36] T. D. Lewin , I. J.‐Y. Liao , and Y.‐J. Luo , “Annelid Comparative Genomics and the Evolution of Massive Lineage‐Specific Genome Rearrangement in Bilaterians,” Molecular Biology and Evolution 41 (2024): msae172, 10.1093/molbev/msae172. 39141777 PMC11371463

[37] J. D. Sigwart , Y. Li , Z. Chen , K. Vončina , and J. Sun , “Still Waters Run Deep in Large‐scale Genome Rearrangements of Morphologically Conservative Polyplacophora,” Elife 13 (2025): RP102542, 10.7554/eLife.102542.2. 40244654 PMC12005716

[38] J. Orvis , C. B. Albertin , P. Shrestha , et al., “The Evolution of Synaptic and Cognitive Capacity: Insights from the Nervous System Transcriptome of *Aplysia* ,” Proceedings of the National Academy of Sciences 119 (2022): 2122301119, 10.1073/pnas.2122301119. PMC9282427 35867761

[39] Y. Wang , Y. Bai , G. Ni , S. Liu , L. Kong , and Q. Li , “Chromosomal Fusions Reshape Gene Expression and Environmental Responses in *Corbicula* Clams,” Journal of Heredity 116 (2025): 788–802, 10.1093/jhered/esaf030. 40377655

[40] J. Sun , H. Zhang , H. Wang , et al., “First Proteome of the Egg Perivitelline Fluid of a Freshwater Gastropod with Aerial Oviposition,” Journal of Proteome Research 11 (2012): 4240–4248, 10.1021/pr3003613. 22738194

[41] Y.‐T. Lin , F. Hui , W. Han , et al., “Chromosome‐level Genome Assembly of Eden's Whale Clarifies the Taxonomy and Speciation of Bryde's Whale Complex,” Molecular Biology and Evolution 42 (2025): msaf234, 10.1093/molbev/msaf234. 40973465 PMC12492004

[42] P. Zhang , Y. Zhao , C. Li , et al., “An Indo‐Pacific Humpback Dolphin Genome Reveals Insights into Chromosome Evolution and the Demography of a Vulnerable Species,” Iscience 23 (2020): 101640, 10.1016/j.isci.2020.101640. 33103078 PMC7569330

[43] S. N. Hoff , M. Maurstad , O. K. Tørresen , et al., “Rapid Genome Modifications Including Chromosomal Fusions And Large‐scale Inversions Are Key Features in Arctic Codfish Species,” Genome Biology 27 (2026): 100, 10.1101/2024.06.28.599280. 41699629 PMC13011446

[44] J. P. Demuth and M. W. Hahn , “The Life and Death of Gene Families,” BioEssays 31 (2009): 29–39, 10.1002/bies.080085. 19153999

[45] P. Balart‐García , L. Aristide , T. M. Bradford , et al., “Parallel and Convergent Genomic Changes Underlie Independent Subterranean Colonization across Beetles,” Nature Communications 14 (2023): 3842, 10.1038/s41467-023-39603-1. PMC10310748 37386018

[46] F. K. Mendes , D. Vanderpool , B. Fulton , and M. W. Hahn , “CAFE 5 Models Variation in Evolutionary Rates among Gene Families,” Bioinformatics 36 (2020): 5516–5518, 10.1093/bioinformatics/btaa1022. 33325502

[47] S. L. Kosakovsky Pond , A. F. Y. Poon , R. Velazquez , et al., “HyPhy 2.5—A Customizable Platform for Evolutionary Hypothesis Testing Using Phylogenies,” Molecular Biology and Evolution 37 (2020): 295–299, 10.1093/molbev/msz197. 31504749 PMC8204705

[48] Y. B. Sade , C. S. Gonçalves , S. M. N. Scapin , et al., “Identification and Characterization of a Glycoside Hydrolase family 9 Member from the Digestive Gland of the Snail *Achatina fulica* ,” Bioenergy Research 15 (2021): 1–13, 10.1007/s12155-021-10303-2.

[49] A. D. Calcino , N. J. Kenny , and M. Gerdol , “Single Individual Structural Variant Detection Uncovers Widespread Hemizygosity in Molluscs,” Philosophical Transactions of the Royal Society B 376 (2021): 0153, 10.1098/rstb.2020.0153. PMC8059565 33813894

[50] L. Wang , L. Wang , H. Zhang , Z. Zhou , V. S. Siva , and L. Song , “A C1q Domain Containing Protein From Scallop Chlamys Farreri Serving as Pattern Recognition Receptor With Heat‐Aggregated IgG Binding Activity,” PLoS ONE 7 (2012): 43289, 10.1371/journal.pone.0043289. PMC3419688 22905248

[51] C. M. Adema , L. W. Hillier , C. S. Jones , et al., “Whole Genome Analysis of a Schistosomiasis‐Transmitting Freshwater Snail,” Nature Communications 8 (2017): 15451, 10.1038/ncomms15451. PMC5440852 28508897

[52] N. Nirmal , D. Demir , S. Ceylan , et al., “Polysaccharides from Shell Waste of Shellfish and Their Applications in the Cosmeceutical Industry: A Review,” International Journal of Biological Macromolecules 265 (2024): 131119, 10.1016/j.ijbiomac.2024.131119. 38522682

[53] G. Imokawa , H. Nakajima , and K. Ishida , “Biological Mechanisms Underlying the Ultraviolet Radiation‐induced Formation of Skin Wrinkling and Sagging II: Over‐expression of Neprilysin Plays an Essential Role,” International Journal of Molecular Sciences 16 (2015): 7776–7795, 10.3390/ijms16047753. 25856676 PMC4425049

[54] C. A. Dinarello , “Interleukin‐1, Interleukin‐1 Receptors and Interleukin‐1 Receptor Antagonist,” International Reviews of Immunology 16 (1998): 457–499, 10.1079/PNS19940044. 9646173

[55] T. Mitros , J. B. Lyons , A. M. Session , et al., “A Chromosome‐Scale Genome Assembly and Dense Genetic Map for *Xenopus tropicalis* ,” Developmental Biology 452 (2019): 8–20, 10.1016/j.ydbio.2019.03.015. 30980799

[56] I. G. Denisov , T. M. Makris , S. G. Sligar , and I. Schlichting , “Structure and Chemistry of Cytochrome P450,” Chemical Reviews 105 (2005): 2253–2278, 10.1021/cr0307143. 15941214

[57] C. Liu , Y. Zhang , Y. Ren , et al., “The Genome of the Golden Apple Snail *Pomacea Canaliculata* Provides Insight Into Stress Tolerance and Invasive Adaptation,” GigaScience 7 (2018): giy101, 10.1093/gigascience/giy101. 30107526 PMC6129957

[58] E. Kruse , N. Uehlein , and R. Kaldenhoff , “The Aquaporins,” Genome Biology 7 (2006): 1–6, 10.1186/gb-2006-7-2-206. PMC1431727 16522221

[59] J. C. Ip , H. Mu , Y. Zhang , H. Heras , and J. W. Qiu , “Egg Perivitelline Fluid Proteome of a Freshwater Snail: Insight Into the Transition from Aquatic to Terrestrial Egg Deposition,” Rapid Communications in Mass Spectrometry 34 (2020): 8605, 10.1002/rcm.8605. 31657488

[60] M. Giglio , C. Garro , E. Caviedes‐Vidal , and H. Heras , “Egg Perivitelline Fluid of the Invasive Snail *Pomacea Canaliculata* Affects Mice Gastrointestinal Function and Morphology,” PeerJ 6 (2018): 5314, 10.7717/peerj.5314. PMC6211264 30397537

[61] M. Y. Pasquevich , M. S. Dreon , and H. Heras , “The Major Egg Reserve Protein from the Invasive Apple Snail *Pomacea Maculata* Is a Complex Carotenoprotein Related to Those of *Pomacea Canaliculata* and *Pomacea Scalaris* ,” Comparative Biochemistry and Physiology Part B: Biochemistry and Molecular Biology 169 (2014): 63–71, 10.1016/j.cbpb.2013.11.008. 24291422

[62] K. Benkendorff , A. R. Davis , and J. B. Bremner , “Chemical Defense in the Egg Masses of Benthic Invertebrates: an Assessment of Antibacterial Activity in 39 Mollusks and 4 Polychaetes,” Journal of Invertebrate Pathology 78 (2001): 109–118, 10.1006/jipa.2001.5047. 11812113

[63] M. L. Giglio , S. Ituarte , A. E. Ibañez , et al., “Novel Role for Animal Innate Immune Molecules: Enterotoxic Activity of a Snail Egg MACPF‐toxin,” Frontiers in Immunology 11 (2020): 428, 10.3389/fimmu.2020.00428. 32231667 PMC7082926

[64] M. L. Giglio , S. Ituarte , V. Milesi , et al., “Exaptation of Two Ancient Immune Proteins into a New Dimeric Pore‐forming Toxin in Snails,” Journal of Structural Biology 211 (2020): 107531, 10.1016/j.jsb.2020.107531. 32446810

[65] T. R. Brola , M. S. Dreon , P. E. Fernández , E. L. Portiansky , and H. Heras , “Ingestion of Poisonous Eggs of the Invasive Apple Snail *Pomacea Canaliculata* Adversely Affects Bullfrog *Lithobathes Catesbeianus* Intestine Morphophysiology,” Malacologia 63 (2021): 171–182, 10.4002/040.063.0202.

[66] J. Zhang , “Evolution by Gene Duplication: an Update,” Trends in Ecology & Evolution 18 (2003): 292–298, 10.1016/S0169-5347(03)00033-8.

[67] F. Shahidi and C. S. Dissanayaka , “Binding of Carotenoids to Proteins: A Review,” Journal of Food Bioactives 24 (2023): 13–28, 10.31665/JFB.2023.18360.

[68] M. Y. Pasquevich , M. S. Dreon , M. E. Diupotex‐Chong , and H. Heras , “Phylogenetic Variations in a Novel family of Hyperstable Apple Snail Egg Proteins: Insights into Structural Stability and Functional Trends,” Journal of Experimental Biology 227 (2024): 247277, 10.1242/jeb.247277. 39022896

[69] K. E. Eastman , A. L. Pendleton , M. A. Shaikh , et al., “A Reference Genome for the Long‐Term Kleptoplast‐Retaining Sea Slug *Elysia Crispata* Morphotype Clarki,” G3: Genes, Genomes, Genetics 13 (2023): jkad234, 10.1093/g3journal/jkad234. 37816307 PMC10700116

[70] J. Li , K. Zheng , W. Ding , et al., “Healthy and Moribund Zhikong Scallops (*Chlamys farreri*) Developed Different Viral Communities during a Mass Mortality Event,” mSystems 10 (2025), 10.1128/msystems.00342-25. PMC12172486 40366141

[71] D. T. Schultz , S. H. Haddock , J. V. Bredeson , R. E. Green , O. Simakov , and D. S. Rokhsar , “Ancient Gene Linkages Support Ctenophores as Sister to Other Animals,” Nature 618 (2023): 110–117, 10.1038/s41586-023-05936-6. 37198475 PMC10232365

[72] S. El Hilali and R. R. Copley , “macrosyntR: Drawing Automatically Ordered Oxford Grids from Standard Genomic Files in R,” BioRxiv (2023): 525673, 10.1101/2023.01.26.525673.

[73] Z. Zhang , “KaKs_Calculator 3.0: Calculating Selective Pressure on Coding and Non‐Coding Sequences,” Genomics, Proteomics & Bioinformatics 20 (2022): 536–540, 10.1016/j.gpb.2021.12.002. PMC9801026 34990803

[74] R. Patro , G. Duggal , M. I. Love , R. A. Irizarry , and C. Kingsford , “Salmon Provides Fast and Bias‐aware Quantification of Transcript Expression,” Nature Methods 14 (2017): 417–419, 10.1038/nmeth.4197. 28263959 PMC5600148

[75] A. M. Bolger , M. Lohse , and B. Usadel , “Trimmomatic: a Flexible Trimmer for Illumina Sequence Data,” Bioinformatics 30 (2014): 2114–2120, 10.1093/bioinformatics/btu170. 24695404 PMC4103590

[76] N. Servant , N. Varoquaux , B. R. Lajoie , et al., “HiC‐Pro: an Optimized and Flexible Pipeline for Hi‐C Data Processing,” Genome Biology 16 (2015): 1–11, 10.1186/s13059-015-0831-x. 26619908 PMC4665391

[77] T. J. Salameh , X. Wang , F. Song , et al., “A Supervised Learning Framework for Chromatin Loop Detection in Genome‐Wide Contact Maps,” Nature Communications 11 (2020): 3428, 10.1038/s41467-020-17239-9. PMC7347923 32647330

[78] X.‐T. Wang , W. Cui , and C. Peng , “HiTAD: Detecting the Structural and Functional Hierarchies of Topologically Associating Domains From Chromatin Interactions,” Nucleic Acids Research 45 (2017): e163–e163, 10.1093/nar/gkx735. 28977529 PMC5737579

[79] F. Serra , D. Baù , M. Goodstadt , D. Castillo , G. J. Filion , and M. A. Marti‐Renom , “Automatic Analysis and 3D‐Modelling of Hi‐C Data Using TADbit Reveals Structural Features of the Fly Chromatin Colors,” PLoS Computational Biology 13 (2017): 1005665, 10.1371/journal.pcbi.1005665. PMC5540598 28723903

[80] J. Wolff , L. Rabbani , R. Gilsbach , et al., “Galaxy HiCExplorer 3: A Web Server for Reproducible Hi‐C, Capture Hi‐C and Single‐cell Hi‐C Data Analysis, Quality Control and Visualization,” Nucleic Acids Research 48 (2020): W177–W184, 10.1093/nar/gkaa220. 32301980 PMC7319437

[81] J. Mistry , S. Chuguransky , L. Williams , et al., “Pfam: the Protein Families Database in 2021,” Nucleic Acids Research 49 (2021): D412–D419, 10.1093/nar/gkaa913. 33125078 PMC7779014

[82] A. Smit , R. Hubley , and P. Green , RepeatMasker, version Open‐4.04.0. Institute for Systems Biology, https://www.repeatmasker.org/.

[83] M. Kimura , “A Simple Method for Estimating Evolutionary Rates of Base Substitutions through Comparative Studies of Nucleotide Sequences,” Journal of Molecular Evolution 16 (1980): 111–120, 10.1007/BF01731581. 7463489

[84] J. C.‐H. Ip , T. Xu , J. Sun , et al., “Host–Endosymbiont Genome Integration in a Deep‐Sea Chemosymbiotic Clam,” Molecular Biology and Evolution 38 (2021): 502–518, 10.1093/molbev/msaa241. 32956455 PMC7826175

[85] Z. Yang , “PAML 4: Phylogenetic Analysis by Maximum Likelihood,” Molecular Biology and Evolution 24 (2007): 1586–1591, 10.1093/molbev/msm088. 17483113

[86] P. Librado , F. G. Vieira , and J. Rozas , “BadiRate: Estimating Family Turnover Rates by Likelihood‐Based Methods,” Bioinformatics 28 (2012): 279–281, 10.1093/bioinformatics/btr623. 22080468

[87] G. Yu , L.‐G. Wang , Y. Han , and Q.‐Y. He , “ClusterProfiler: An R Package for Comparing Biological Themes among Gene Clusters,” OMICS: A Journal of Integrative Biology 16 (2012): 284–287, 10.1089/omi.2011.0118. 22455463 PMC3339379

[88] F. Thalén . “PhyloPyPruner: Tree‐based Orthology Inference for Phylogenomics with New Methods for Identifying and Excluding Contamination,” LUND UNIVERSITY LIBRARIES (2018).

[89] K. Katoh and D. M. Standley , “MAFFT Multiple Sequence Alignment Software Version 7: Improvements in Performance and Usability,” Molecular Biology and Evolution 30 (2013): 772–780, 10.1093/molbev/mst010. 23329690 PMC3603318

[90] M. Suyama , D. Torrents , and P. Bork , “PAL2NAL: Robust Conversion of Protein Sequence Alignments into the Corresponding Codon Alignments,” Nucleic Acids Research 34 (2006): W609–W612, 10.1093/nar/gkl315. 16845082 PMC1538804

[91] S. Chen and Z. Zou , “Detecting Convergence of Amino Acid Physicochemical Properties Underlying the Organismal Adaptive Convergent Evolution,” Molecular Ecology Resources 25 (2025): 70052, 10.1111/1755-0998.70052. PMC12550464 41001784

[92] A. Criscuolo and S. Gribaldo , “BMGE (Block Mapping and Gathering With Entropy): A New Software for Selection of Phylogenetic Informative Regions From Multiple Sequence Alignments,” BMC Evolutionary Biology (2010): 1–21, 10.1186/1471-2148-10-210. 20626897 PMC3017758

[93] R. Bouckaert , J. Heled , D. Kühnert , et al., “BEAST 2: A Software Platform for Bayesian Evolutionary Analysis,” PLoS Computational Biology 10 (2014): 1003537, 10.1371/journal.pcbi.1003537. PMC3985171 24722319

[94] D. Posada and K. A. Crandall , “MODELTEST: Testing the Model of DNA Substitution,” Bioinformatics 14 (1998): 817–818, 10.1093/bioinformatics/14.9.817. 9918953

[95] A. Rambaut , A. J. Drummond , D. Xie , G. Baele , and M. A. Suchard , “Posterior Summarization in Bayesian Phylogenetics Using Tracer 1.7,” Systematic Biology 67 (2018): 901–904, 10.1093/sysbio/syy032. 29718447 PMC6101584

[96] Z. Zhang , J. Xiao , J. Wu , et al., “ParaAT: A Parallel Tool for Constructing Multiple Protein‐Coding DNA Alignments,” Biochemical and Biophysical Research Communications 419 (2012): 779–781, 10.1016/j.bbrc.2012.02.101. 22390928

[97] J. Su , Y. He , S. You , et al., “A Trimodal Protein Language Model Enables Advanced Protein Searches,” Nature Biotechnology (2025): 1–7, 10.1038/s41587-025-02836-0. 41039041

[98] M. Van Kempen , S. S. Kim , C. Tumescheit , et al., “Fast and Accurate Protein Structure Search With Foldseek,” Nature Biotechnology (2024): 243–246, 10.1038/s41587-023-01773-0. PMC10869269 37156916

