# Supplementary material for: Two Routes to Land: Genomic Underpinnings of Parallel Aerial Egg Deposition in Aquatic Old‐World Pila and New‐World Pomacea (Ampullariidae)
Source: Adv Sci (Weinh). 2026 Jun 2:e22371. Online ahead of print. doi: 10.1002/advs.202522371 (PMC13336756; doi:10.1002/advs.202522371)
Supplement: Supplementary file 1 — Supporting File 1: advs75951‐sup‐0001‐Figure S1‐S30.doc. [file ADVS-9999-e22371-s001.doc]

**Supplementary Material**

**Two routes to land: Genomic underpinnings of parallel aerial egg deposition in aquatic Old-World *Pila* and New-World *Pomacea* (Ampullariidae)**

Yufei Zhou^#^, Huawei Mu^#^, Xueying Nie, Yue Gao, Hui Wang, Ling Fang, Tiangang Luan, Monthon Ganmanee, Jian-Wen Qiu*, Jin Sun*, Jack Chi-Ho Ip*

# These authors contributed equally.

*Author for correspondence

**This Supplementary Material includes:**

1. Materials and Methods

2. Supplementary Figs. S1 to S27

3. References

**1.** **Materials and Methods**

*PVF extraction and LC-MSMS analysis*

PVF analysis of two Old-World *Pila* and *Lanstis* was performed following our previous proteomic studies [1, 2]. PVF was extracted from approximately 30 eggs using a fine needle, mixed with 8 M urea, and centrifuged at 12,000×g for 10 min at 4 °C to obtain the protein-containing supernatant. Protein samples were combined with a loading buffer (20% glycerol, 0.2M Tris-HCl pH 6.8, 0.05% bromophenol blue, 10mM dithiothreitol, and 10% SDS) at a 1:3 (v/v) ratio, separated by SDS-PAGE, stained with Coomassie Brilliant Blue, and destained with 1% acetic acid. Each gel was cut into eight slices based on intensity and molecular weight, further destained with 50mM NH4HCO3 in 50% methanol, washed with MilliQ water, dried, and rehydrated twice with 100% acetonitrile and 100mM NH4HCO3. In-gel digestion was performed using sequencing grade trypsin (Promega, Madison, USA) in 50mM NH4HCO3, and peptides were recovered, desalted using Sep-Pak C18 cartridges (Waters, Milford, USA), and dried in a vacuum concentrator (Eppendorf, Hamburg, Germany).

The dried fraction from each biological sample was reconstituted with 0.1% formic acid and analyzed twice using an LTQ-Orbitrap Elite coupled with an Easy-nLC (Thermo Fisher, Bremen, Germany). Peptides were separated on a C18 capillary column (Michrom BioResources, CA) over a 90-min gradient. Mass spectrometry scans were performed in the range of 350 to 1600 m/z at a resolution of 60,000 in positive mode. The five most abundant multiple-charged ions with a minimum signal of 500.0 were selected for high-energy collision-induced dissociation (HCD) and fragmentation via collision-induced dissociation (CID), both using an isolation width of 2.0 m/z. HCD had an activation time of 10 ms and a normalised collision energy of 45%, while CID had the same activation time but a normalised collision energy of 35%.

MaxQuant v2.6.5 was used to search the raw MS data against a protein database obtained by translating genomes (*Lanistes nyassanus*, *P. canaliculata*, *P. maculata*, *P. diffusa*, *M. cornuarietis*, *Pila pesmei*) and transcriptomes (*P. scalaris*) that contained protein sequences (target) and their reversed sequences (decoy) [3]. Search parameters were 20 ppm for the first search mass tolerance; 0.5 Da for fragments; two maximum missed cleavages for trypsin. The matched peptides were further filtered to delete reverse and potential contamination using Perseus v2.1.3 [4]. A false discovery rate threshold of < 0.01 was also applied in each replicate for final protein identification. Only proteins that were identified in at least two biological replicates, contained at least 2 unique peptides were retained.

To further confirm the number of PV1 subunit, MSGFPlus searches (https://github.com/MSGFPlus/msgfplus) were performed for the ovorubin MS data of *P. canaliculata* purified by Horacio Heras team, with following parameters: ±20 ppm parent mass tolerance; isotope error range (-ti ‘-1,2’); fully tryptic enzyme settings (-e 1 -ntt 2); conducting a parallel search against a decoy protein database (-tda 1) for calculating the false discovery rate (FDR). Only proteins that were identified in three biological replicates and contained at least 3 unique peptides were retained.

*Genome sequencing*

Genomic DNA was extracted using the CTAB method [5]. DNA quality was evaluated, and quantity measured using agarose gel electrophoresis and a Qubit fluorometer (Thermo Fisher Scientific, MA, USA), respectively. High-quality DNA was then used for library preparation and whole-genome sequencing (details in **Table S1**).

For *Pila celebensis*, 1 µg of DNA was used to construct a library with a 350-bp insert size using the NEBNext DNA Library Prep Kit (New England Biolabs, MA, USA), and sequenced on an Illumina NovaSeq with paired-end 150 bp mode (PE150). Additionally, 10 µg of DNA was used to construct a HiFi SMRTbell library using the SMRTbell Express Template Prep Kit 2.0, which was sequenced on a PacBio Sequel II sequencer. High-quality HiFi reads were generated using the circular consensus sequencing (CCS) mode on the PacBio long-read platform. For Hi-C sequencing, the foot tissue was re-frozen on ice and mixed with 37% formaldehyde in serum-free Dulbecco's modified Eagle's medium DMEM. The tissue was then homogenised, treated with a restriction enzyme (MBOI), labelled with biotin, and repaired. DNA was extracted and purified for library preparation, aiming for a 350-bp insert size, using the NEBNext DNA Library Prep Kit. Finally, it was sequenced on an Illumina NovaSeq in PE150 mode.

For *Pila pesmei*, 5 µg of DNA was used to construct libraries with 250-bp and 500-bp insert sizes using the NEBNext DNA Library Prep Kit (New England Biolabs, MA, USA), as well as a mate-pair library with a 15 kb insert size using the Nextera Mate Pair Library Preparation Kit (Illumina, USA). These were sequenced on an Illumina HiSeq 2500 in PE150 mode. For long-read sequencing, 5 μg of genomic DNA was used to construct a long-length DNA library using the Ligation Sequencing Kit 1D (SQK-LSK109, ONT, Oxford, UK) according to the product’s instructions, and sequenced using one FLO-MIN106 R9.4 flow cell coupled to a GridION X5 sequencer (ONT, Oxford, UK). The raw reads were real-time base-called using Guppy v1.6.0 (ONT) under default settings to generate a FASTQ file.

Total RNA was extracted from three individuals of *Pila celebensis* using Trizol reagent (Thermo Fisher Scientific, MA, USA), following the manufacturer’s protocol. The quality of the RNA samples was assessed by agarose gel electrophoresis, and the quantity was measured using a Qubit fluorometer (Thermo Fisher Scientific, MA, USA). RNA samples were submitted to Novogene (Beijing, China) for cDNA library preparation using the Illumina NEBNext Ultra RNA Library Prep Kit (New England Biolabs, MA, USA) and sequenced on an Illumina NovaSeq in PE150 mode. RNA-seq data for *Pila pesmei* (named as *Pila ampullacea* in AmpuBase) were obtained from Ip et al., 2018 [1].

*Genome assembly*

Illumina short reads were trimmed to remove adaptors and low-quality reads (quality score <30, length <40 bp) using Trimmomatic v0.38 [6]. The clean Illumina reads were used to estimate the genome sizes of the two *Pila* species using GenomeScope v2.0 [7]. For *Pila celebensis*, *de novo* assembly of HiFi reads (N50 of 9.853 Mb and mean length of 2.578 Mb; **Table S1**) were performed using nextDenovo v2.5.2 under default settings [8]. Possible alternative heterozygous contigs were further eliminated using purge_dups v1.2.6 [9]. Possible contamination sequences (e.g., microbes, fungi, algae and viruses) were removed by search using BLASTn against the NCBI database with an E-value threshold of 1e-20 and manual correction. The completeness of the final genome assembly was assessed by analyzing the Benchmarking Universal Single-Copy Orthologs (BUSCO) v5.4.5 scores against the database metazoan_odb10 under the genome mode [10]. QUAST v5.2 was used to assess assembly statistics [11]. To further scaffold the assembly at a chromosomal level, Hi-C raw reads were trimmed using Trimmomatic v0.38 to remove low-quality reads (quality score < 30, length < 40 bp). High-quality reads were then identified with HiC-Pro v2.10 and duplicates were removed using the Juicer pipeline v1.5 [12, 13]. Next, genomic scaffolding was performed with the 3D *de novo* assembly pipeline for diploid genomes [14]. Pseudo-chromosomal linkage groups were reviewed, and corrections were made in Juicebox v1.11.08 to ensure that scaffolds within the same groups matched the Hi-C linkage characteristics [13]. RaGOO v1.1 was used to scaffold the final assembly into pseudo-chromosomal linkage groups based on *Pila celebensis* genome [15].

For *Pila pesmei*, the FM-index Long Read Corrector (FMLRC) was used to correct the ONT raw reads using Illumina reads [16]. After trimming and error correction, a total of 50 Gb Illumina and 10 Gb ONT reads (N50 of 12.3 kb and mean length of 5.7 kb; **Table S1**) were retained. The hybrid assembly was conducted with MaSuRCA v3.4.1 using both Illumina and ONT data [17]. Redundances were applied to eliminate alternative heterozygous contigs [18]. The resulting contigs were further scaffolded with ONT data using SSPACE-LongRead v1.1 [19]. Potential contamination sequences were removed by searching with BLASTn against the NCBI database. Genome completeness and statistics were evaluated using BUSCO and QUAST, respectively.

*Genome annotation*

The *Pila* genomes were annotated according to Sun et al., and Xiong et al., In brief, the genome was “soft-masked” using RepeatMasker v4.1.0 [20]. With the repeat libraries of all model organisms in the Dfam version 201810, and species-specific repeat libraries in RepeatModeler2 and EDTA v2.1.0 [21, 22]. Mollusca protein sequences from Swiss-Prot and Uniprot databases and five related-species protein sequences (*P. canaliculata*, *P. maculata*, *P. diffusa*, *M. cornuarietis* and *Lanistes nyassanus*) were used in miniprot v0.14 as protein evidence [23]. To provide transcriptomic evidence, *de novo* and genome-guided transcriptomes of two *Pila* were assembled using Trinity v2.15.1 under default settings, respectively [24]. These two transcriptomes were merged using the PASA pipeline v2.5.3 with the “--ALIGNERS blat,gmap,minimap2” setting [25]. BRAKER3 was used to *ab initio* predict genes in the repeat-masked genome sequences [26]. Results from different gene predictors were integrated into a consensus weighted annotation by EVidenceModeler (EVM) v2.1.0 with “PROTEIN = 10, TRANSCRIPT = 8, ABINITIO_PREDICTION = 3” and default settings for other parameters in the software [27]. GeMoMa v1.9 was used for protein homology annotation, afterwards, the annotation results from EVM and GeMoMa were combined using miniprot [28]. Then PASA was used to improve the annotated gene models by modifying gene structures and adding UTR annotations using the de novo and genome-guided transcriptome. The predicted genes were functionally annotated using Diamond BLASTp v2.1.0 under an E-value threshold of 1e-10 against the NCBI non-redundant (nr) database [29]. Gene functional annotation was conducted using Blast2GO Basic 6.0 and the KAAS website (<https://www.genome.jp/kaas-bin/kaas_main>) for Gene Ontology (GO) and Kyoto Encyclopedia of Genes and Genomes (KEGG) pathways [30].

*Phylogenomics and divergence times*

Phylogenetic analysis was conducted according to Sun et al [31]. Briefly, orthologous groups (OGs) among seven ampullariids and 16 molluscan genomes were inferred using OrthoFinder v.2.5.4 with Diamond BLASTp v2.1.0 under “--ultra-sensitive” mode [32]. One genome each from Bivalvia (*Mizuhopecten yessoensis*), Polyplacophora (*Acanthopleura granulata*), and Cephalopoda (*Nautilus pompilus*) was used as outgroups of Gastropoda. Only OGs with at least 80% taxon representation (i.e., at least 18 species) were used to reconstruct their phylogenetic relationships. The protein sequences were aligned using MAFFT under default settings and trimmed using BMGE v1.12, and the alignments with fewer than 20 amino acids were removed [33]. Each alignment was used to construct “approximately maximum likelihood” tree using FastTree version 2.1.11 [34]. And then PhyloPyPruner v1.2.4 (https://pypi.org/project/phylopypruner/) was used to remove paralogues. Phylogenetic analysis was conducted using a maximum-likelihood method implemented in IQ-TREE v2.2.6 (Minh et al., 2020) with the “-MFP” model and partitioned alignment to compute the best-fit model of each partition and 1000 ultrabootstraps to test the topological support [35]. In addition, divergence times were estimated using MCMCTree v4.10.3 with various time constraints: the ‘root-age’ was set as 532 MYA; a soft constraint of 520.5-530 MYA for the origin of Bivalvia; a hard lower bound of 470.2 MYA and a soft upper bound of 531.5 MYA for the first appearance of Gastropoda; a hard minimum 390 MYA bound for the split of Caenogastropoda and Heterobranchia; a hard upper bound of 150 MYA for the split of *Lanistes nyassanus*; and a hard lower bound of 130 MYA for the first appearance of both the Stylommatophora and Hygrophila [36-39].

*New genome assemblies reveal the evolution trajectory of PV2 subunits*

Genomic analyses reveal an expanded tachylectin gene family (5–8 copies) in *Pomacea* species—functionally linked to the MACPF chain for embryonic protection [40]. Our previous study hypothesized the evolutionary history of two PV2 subunits, with the updated chromosomal-level genomes of *Pomacea* and *Marisa*, we have reconstructed the evolutionary trajectory of PV2 [41, 42]. Briefly, we identified 41 MACPF homologs across eight ampullariids and sixteen molluscs. Maximum-likelihood phylogenetics resolved two clades (Fig. S21A): Clade I (12 ampullariid sequences) nested among non-ampullariid sequences, while Clade II (29 ampullariid-specific homologs) underwent extensive duplication in New-World snails. PV2-associated MACPF genes (Pcan_009G001433; Pmac_011G001277) evolved within Clade II, exhibiting albumen gland-specific high expression (TPM > 500) and PVF proteomic detection (Table S17). Divergence dating places their origin at ~6 million years ago, postdating the *P. diffusa* split with *P. canaliculata* and *P. maculata* (Fig. S26). Thus, neofunctionalization after duplication—specifically, MACPF secretion by the albumen gland and partnership with tachylectin—drove PV2 toxin emergence in terrestrial-egg-laying *Pomacea*.

Tachylectin-like genes appear specific to Caenogastropoda, with 66 homologs identified in ampullariids and 11 in *Littorina sinensis* and *Rapana venosa*, but undetected in other molluscan species. Maximum-likelihood phylogenetics divided these into two clades (Fig. S21B): Clade II contains six ampullariid sequences that high expression in albumen gland (one each in *P. scalaris*, *P. maculata*, and *P. canaliculata*, plus three in *M. cornuarietis*; TPM >500), while Clade I comprises 25 ampullariid sequences featuring a terminal *Pomacea*-specific group of 20 genes. Divergence dating revealed that Clade I underwent extensive duplication ~30 Ma after *Pomacea* diverged from other ampullariids (Fig. S28), with three *Pomacea* genes (Pcan_009G001432, Pmac_011G001273, Pmac_011G001275) exhibiting both high albumen gland expression and PVF encoding. These originated 6-8 Ma—contemporaneous with PVF-associated MACPF evolution—supporting neofunctionalization after duplication as the mechanism for the novel tachylectin subunit in Canaliculata eggs.

Genome screening reveals that at least one scaffold/chromosome contains both MACPF-like and tachylectin-like genes in all six ampullariids (Fig. S22). This two-gene configuration, present in *Pila celebensis* and *L. nyassanus*, likely existed as a single copy in the ampullariid ancestor. Following the divergence of *M. cornuarietis* and *Pomacea* lineages (~34 Ma; Figs. S26-S29), *M. cornuarietis* acquired species-specific MACPF duplications. Extensive duplications then occurred in *Pomacea* post-divergence, exemplified by at least three tandemly duplicated MACPF-tachylectin pairs on *P. canaliculata* chromosome 9 (Fig. S30), suggesting coordinated duplication events. Within the Canaliculata clade, specific genes (Pcan_009G001433, Pmac_011G001277, Pcan_009G001432, Pmac_011G001273, Pmac_011G001275) underwent positive selection post-*P. diffusa* divergence. These exhibit both high albumen gland expression and MACPF-tachylectin complex binding sites, explaining restriction of PV2 within Canaliculata clade [40]. Overall, the occurrence of PV2 in Canaliculata eggs—and its toxicity—indicates a defensive role against terrestrial predators. The co-selection of PV2 and terrestrial egg deposition represents a key innovation enabling their aquatic-to-terrestrial transition and contributes significantly to the global invasiveness of *Pomacea*.

**2. Supplementary Figs. S1 to S18**


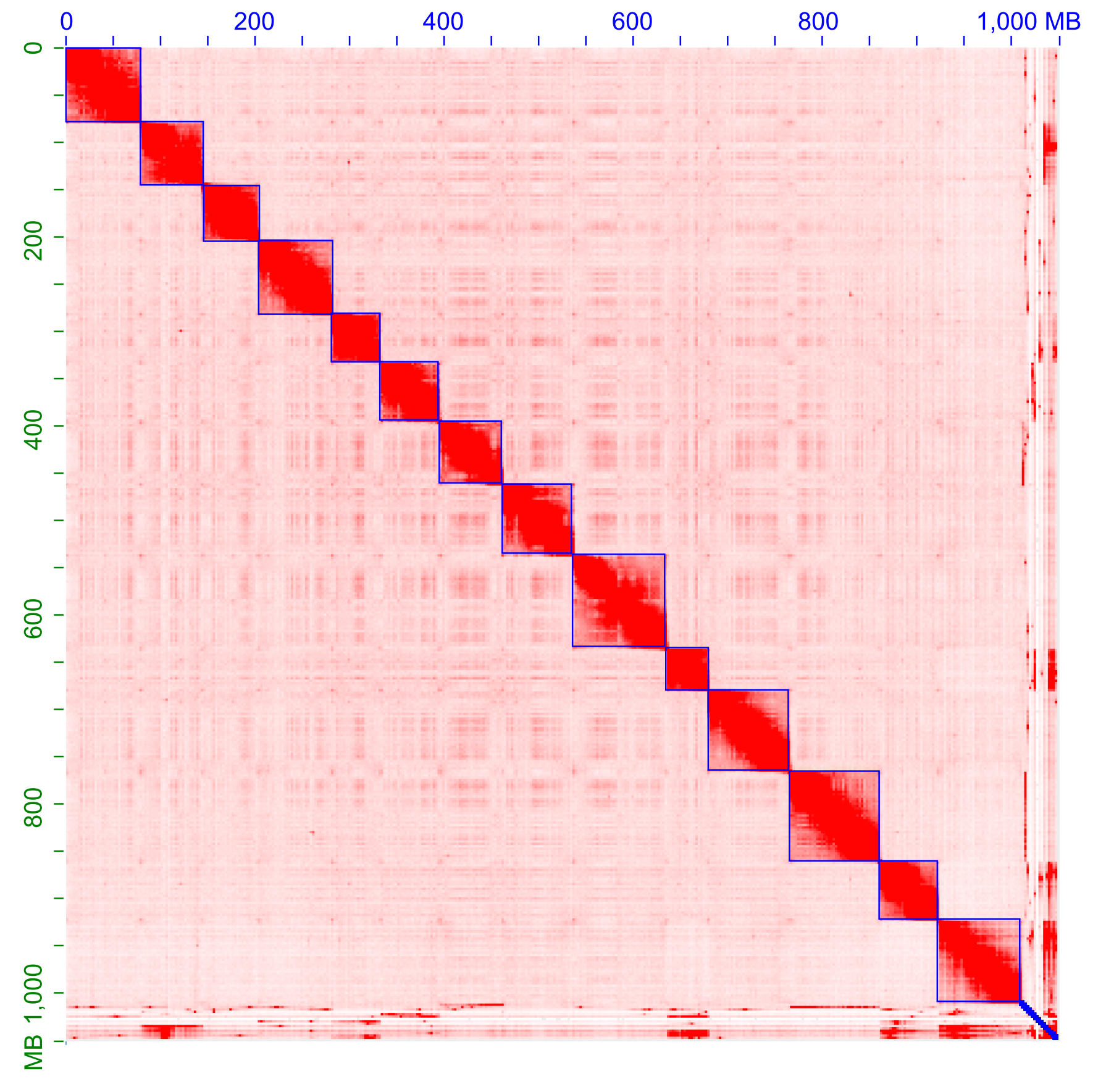


**Fig. S1.** **Chromosome-Scale genome assemblies of *Pila celebensis*.** Hi-C interaction heatmaps depicting the chromosomal organization of the final genome assemblies of *Pila celebensis*, showing contact frequency across scaffolds.

**Fig. S2.** The distribution of the synonymous substitution rate (Ks) of homologous gene groups for intraspecies and interspecies comparisons.

| 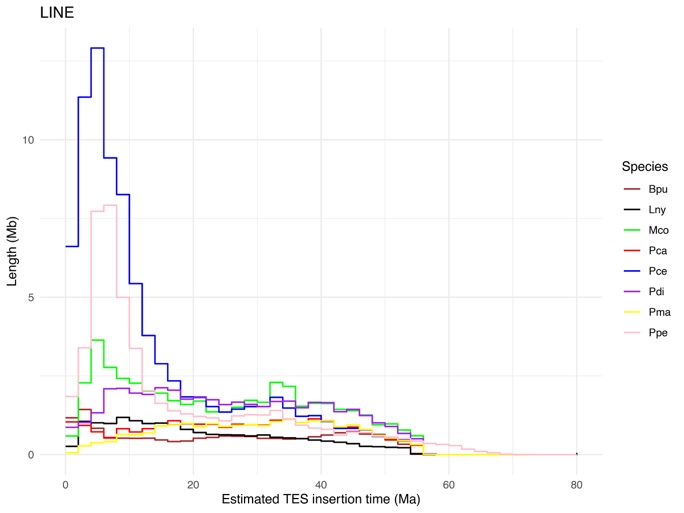 | 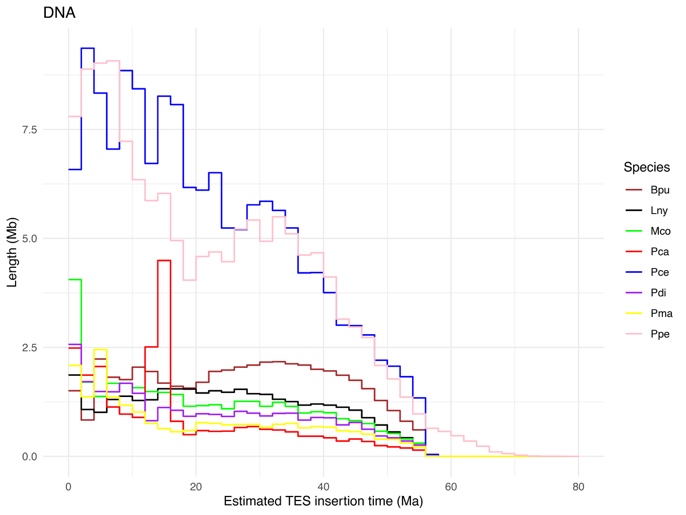 |
| --- | --- |
| 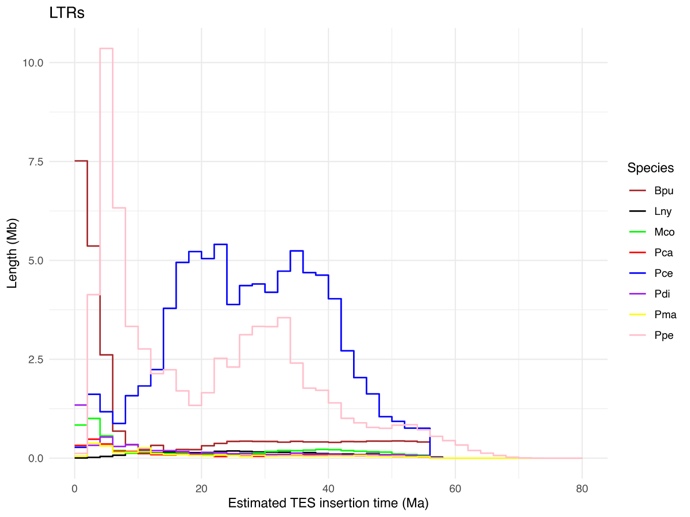 | 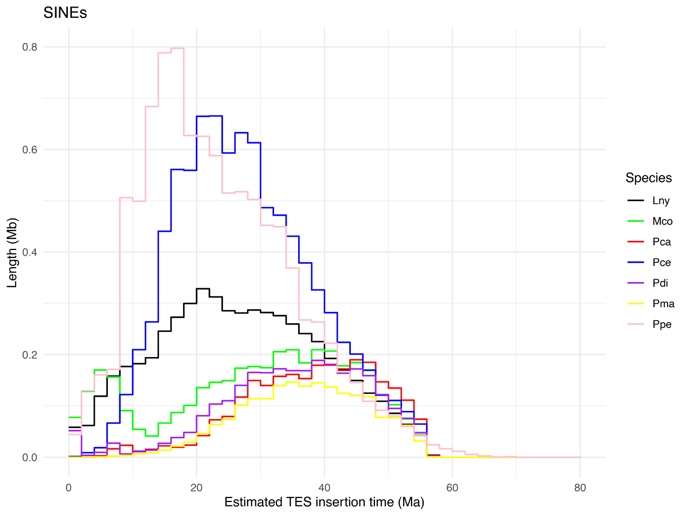 |

**Fig. S3.** Comparison of estimated insertion times of four transposable element (TE) classes among Ampullariidae and *Bellamya purificata*, showing that the genome of *Pila celebensis* is distinct in that the major bursts of SINEs and LTRs corresponded to the time of the split between *Pila* and *Lanistes* (20-40 Ma).

**
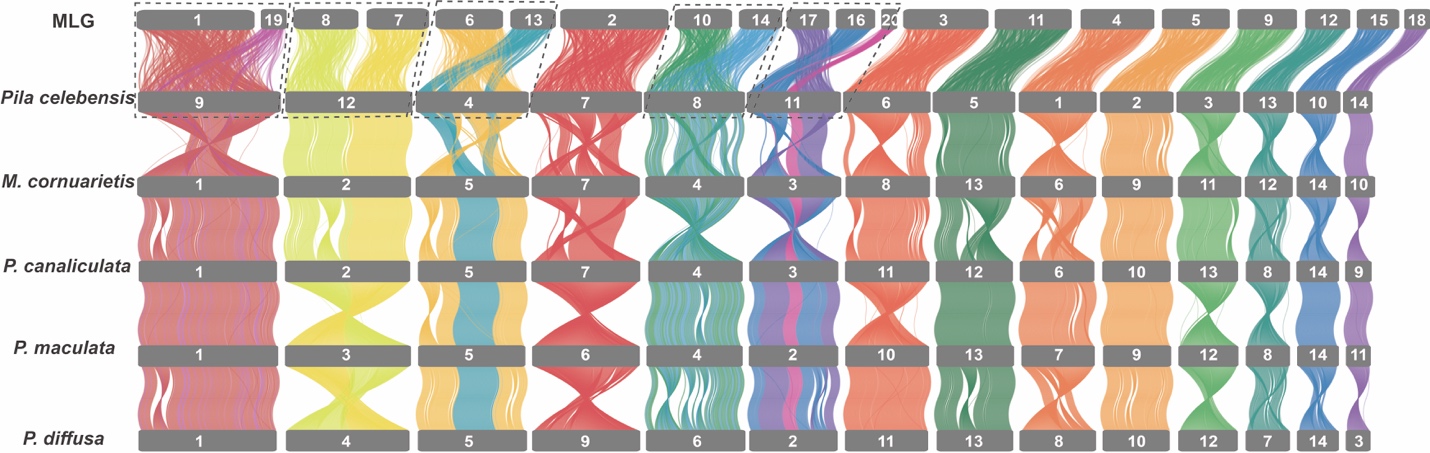
**

**Fig. S4.** Syntenic rearrangements of molluscan linkage groups (MLGs) within the evolution of ampullariid genomes, with conserved syntenic regions, genome rearrangements, and structural variations among these species in relation to the ancestral genome organization. A total of five fusion events were identified from the MLGs to the ancestor of Ampullariidae species, the five fusion events framed by the black dotted line. These fusions include two fusion-with-mixing events (MLG01 + MLG19 and MLG10 + MLG14), two fusion-without-mixing events (MLG07 + MLG08 and MLG06 + MLG13), and one fusion-without-mixing event involving MLG17 + MLG16, with MLG20 inserted in the middle. These patterns are consistent with chromosomal evolution observed in New-World species.


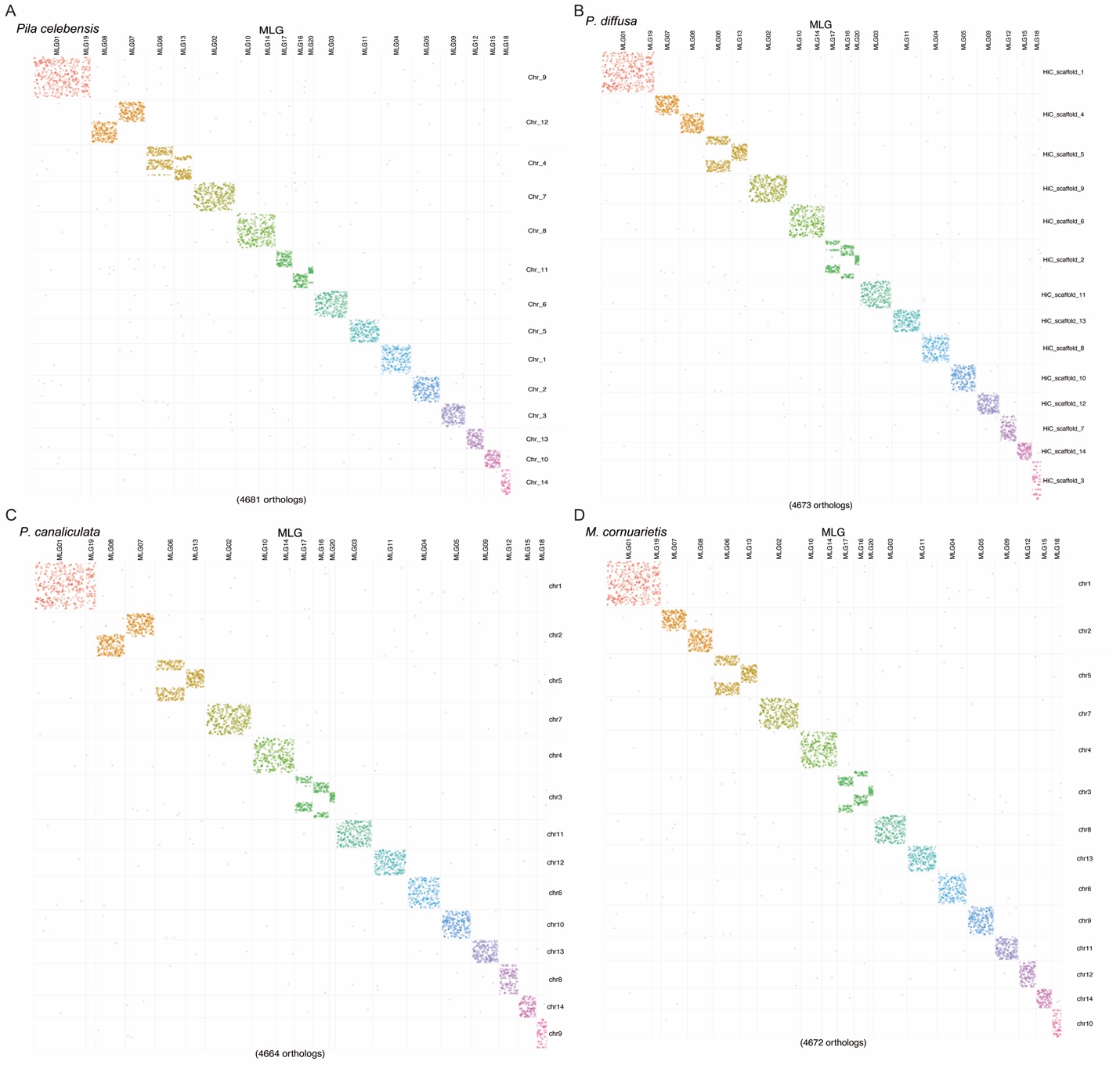


**Fig. S5.** Oxford dotplots of molluscan linkage groups (MLGs) with four Ampullariidae species, *Pila celebensis*, *Pomacea diffusa*, *Pomacea canaliculata*, *Marisa cornuarietis*.

| 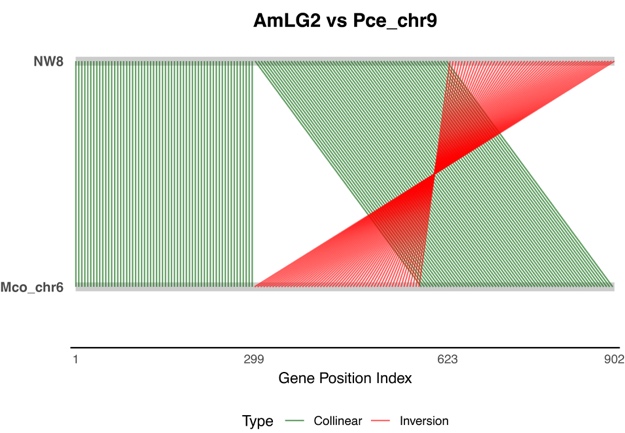 | 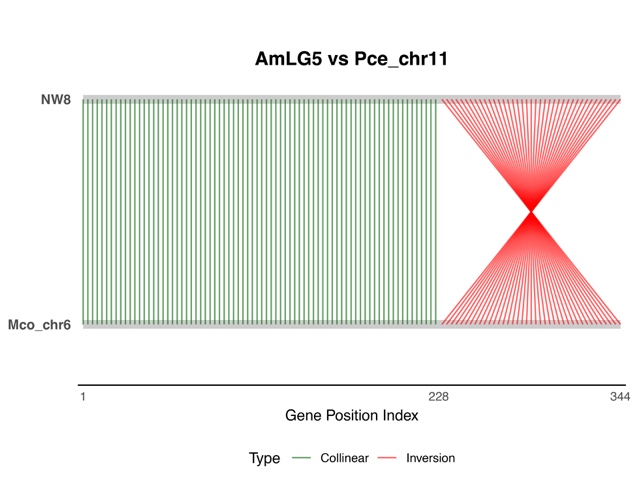 |
| --- | --- |

**Fig. S6.** Schematic representation of chromosomal rearrangement in *Pila celebensis*. The synteny plot illustrates the gene-to-gene mapping between the ancestral linkage group (AmLG) and *Pila celebensis* chromosome 1. Green lines indicate collinear regions where the gene order is conserved. The red lines highlight a specific chromosomal rearrangement event, where the gene order is reversed relative to the ancestral linkage group. The X-axis represents the gene position index.

| 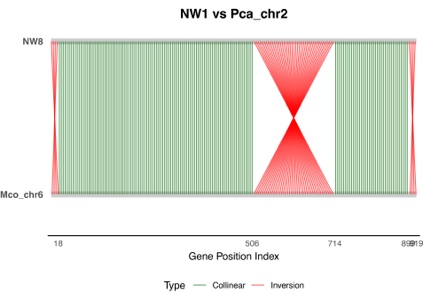 | 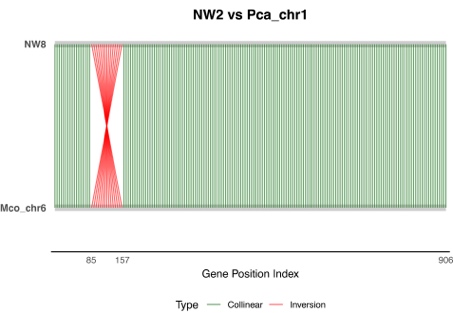 | 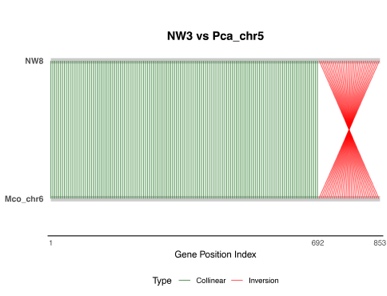 |
| --- | --- | --- |
| 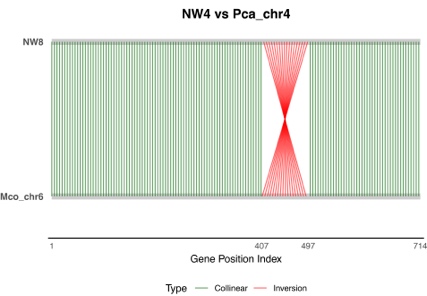 | 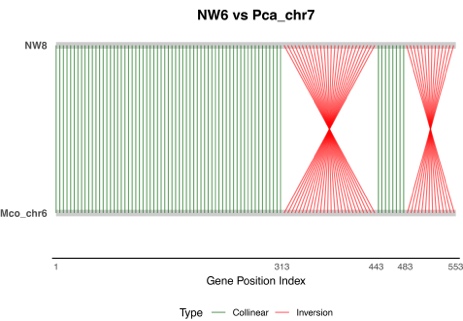 | 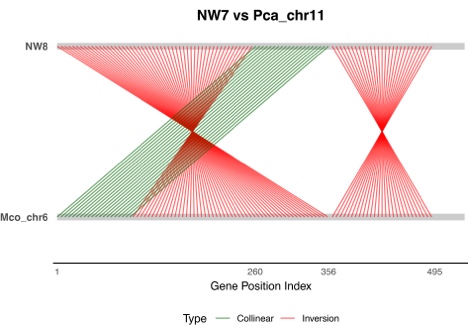 |
| 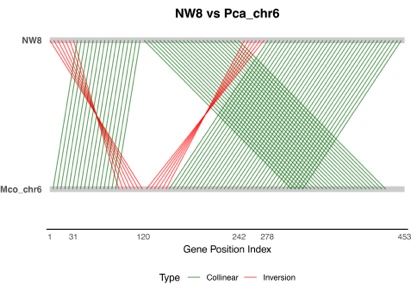 | 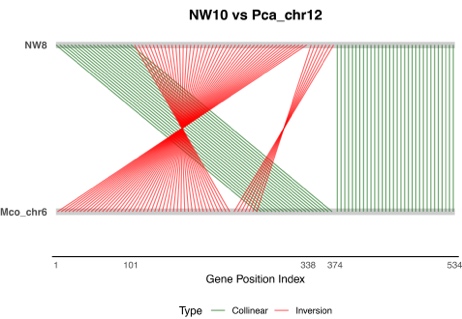 | 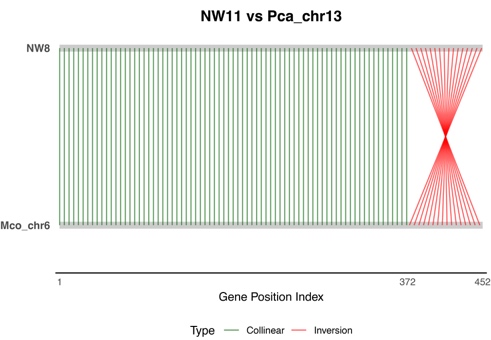 |
| 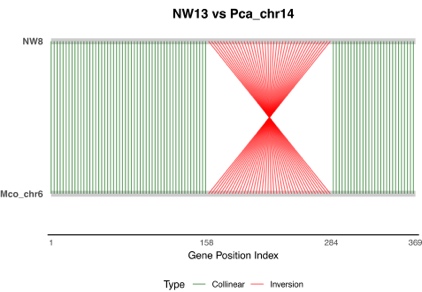 |  |  |

**Fig. S7.** Schematic representation of chromosomal rearrangement in *Pomacea canaliculata*. The synteny plot illustrates the gene-to-gene mapping between New-World lineage MLGs and *Pomacea canaliculata* chromosome. Green lines indicate collinear regions where the gene order is conserved. The red lines highlight a specific chromosomal rearrangement event, where the gene order is reversed relative to the ancestral linkage group. The X-axis represents the gene position index.

| 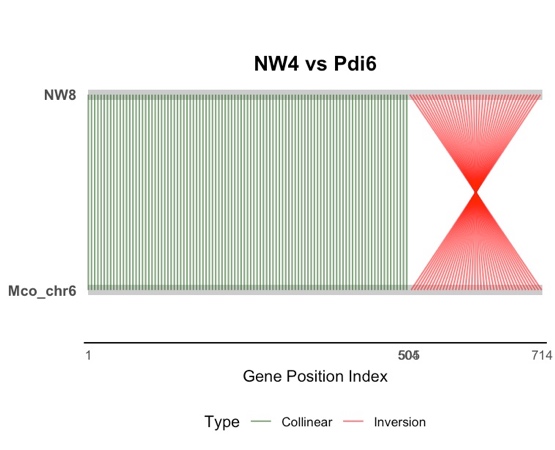 | 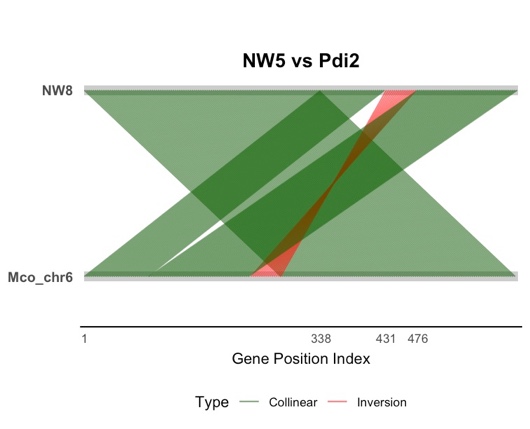 |
| --- | --- |
| 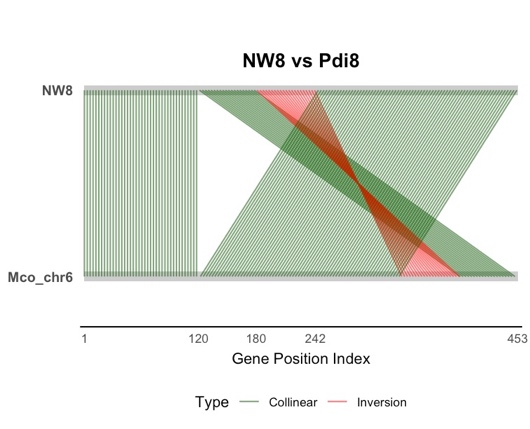 | 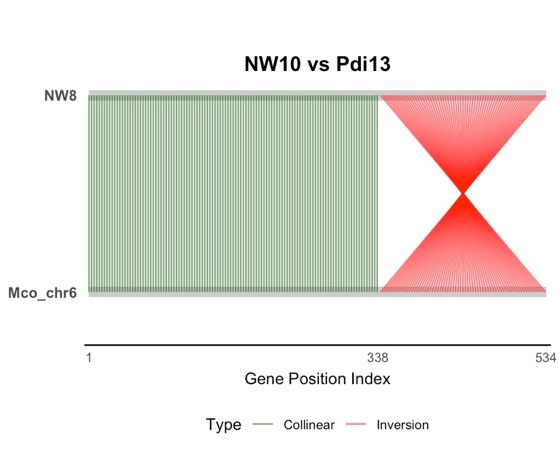 |

**Fig. S8.** Schematic representation of chromosomal rearrangement in *Pomacea diffusa*. The synteny plot illustrates the gene-to-gene mapping between New-World lineage MLGs and *Pomacea diffusa* chromosome. Green lines indicate collinear regions where the gene order is conserved. The red lines highlight a specific chromosomal rearrangement event, where the gene order is reversed relative to the ancestral linkage group. The X-axis represents the gene position index.

| 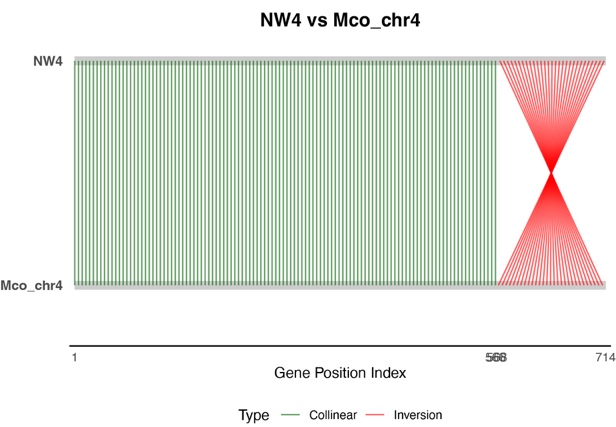 | 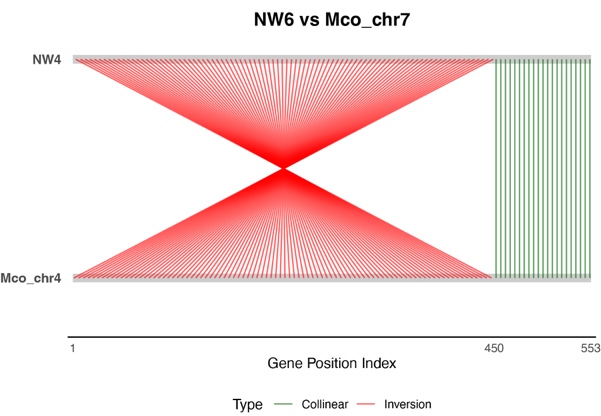 |
| --- | --- |
| 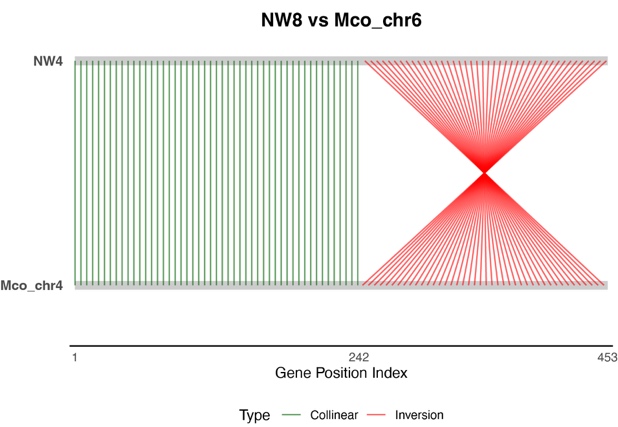 | 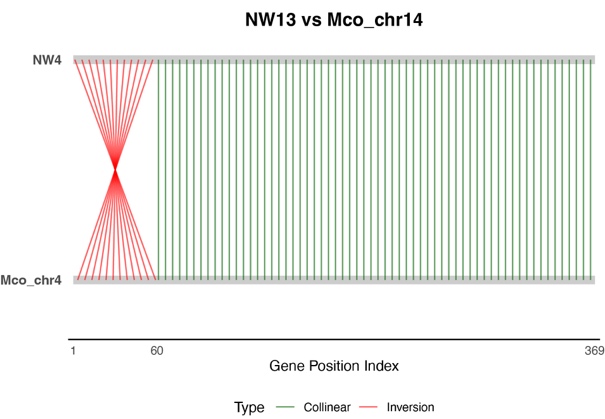 |

**Fig. S9.** Schematic representation of chromosomal rearrangement in *Marisa cornuarietis*. The synteny plot illustrates the gene-to-gene mapping between New-World lineage MLGs and *Marisa cornuarietis* chromosome. Green lines indicate collinear regions where the gene order is conserved. The red lines highlight a specific chromosomal rearrangement event, where the gene order is reversed relative to the ancestral linkage group. The X-axis represents the gene position index.


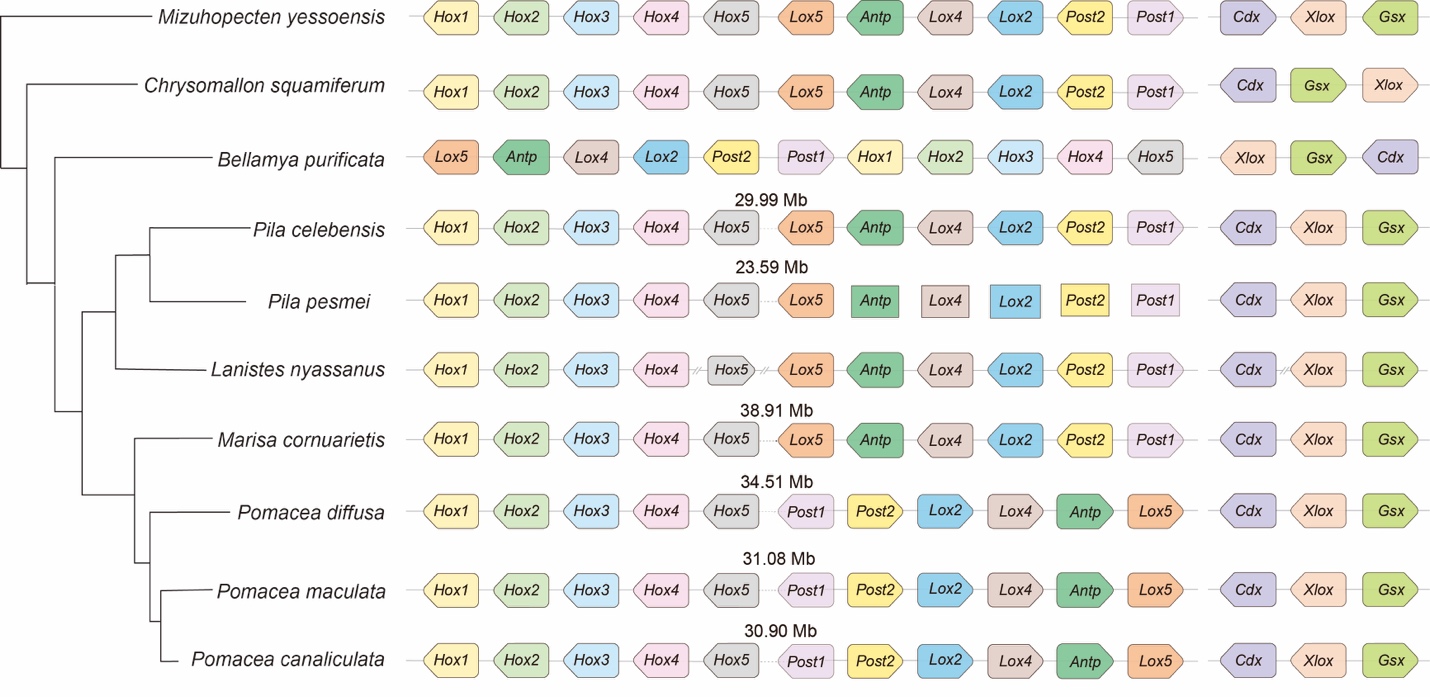


**Fig. S10.** Schematic illustration of Hox and ParaHox gene clusters in seven ampullariid species and three selected mollusks. Colored boxes represent individual genes with names labeled in the center. Solid lines connect genes located on the same scaffold or chromosome (note: line length is not proportional to physical sequence length). Dotted lines indicate that genes reside on the same chromosome but are separated by a substantial genomic interval (approximately 30 Mb). Gaps and "//" symbol between genes indicate that they are located on different scaffolds or chromosomes. Where available, the direction of transcription is indicated by arrowheads (or "pointed ends").

| *Pila celebensis* | | |
| --- | --- | --- |
| 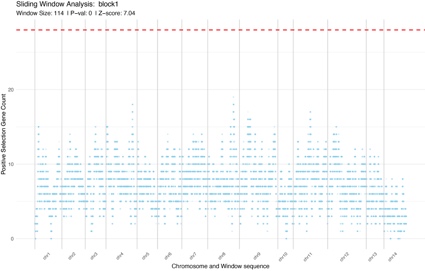 | 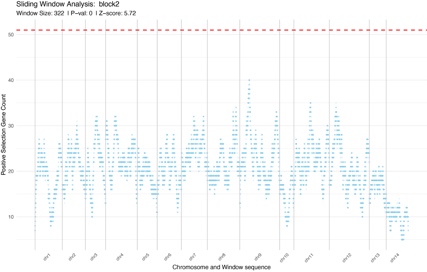 | 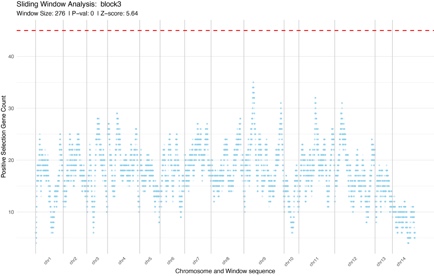 |
| *Pomacea canaliculata* |  |  |
| 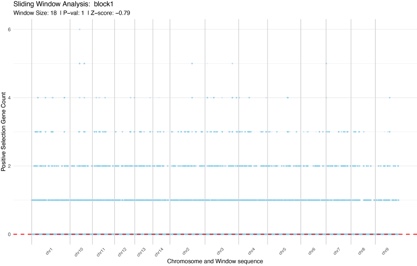 | 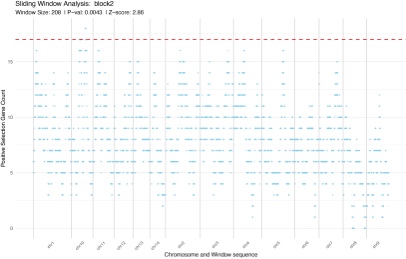 | 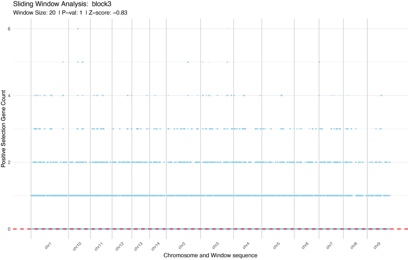 |
| 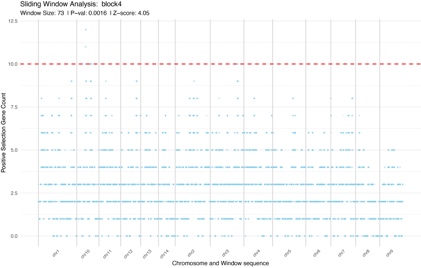 | 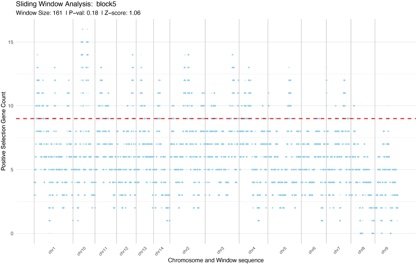 | 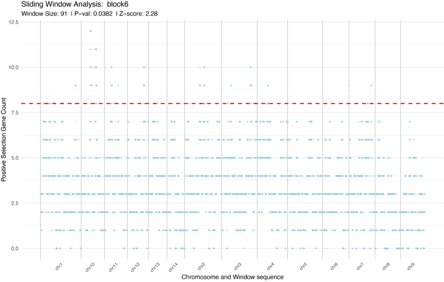 |
| 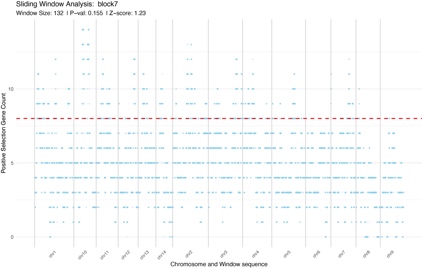 | 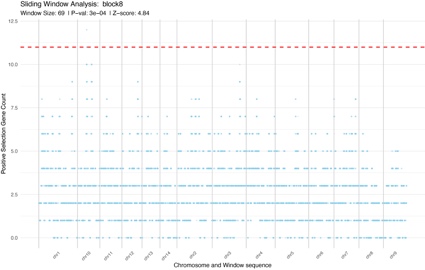 | 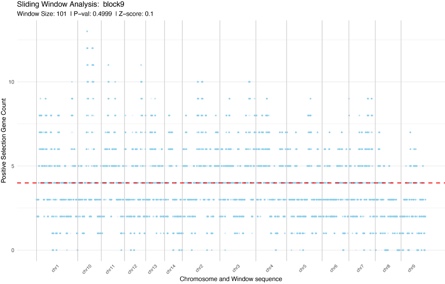 |
| 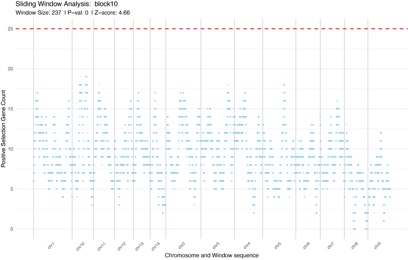 | 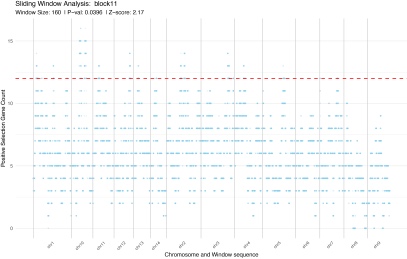 | 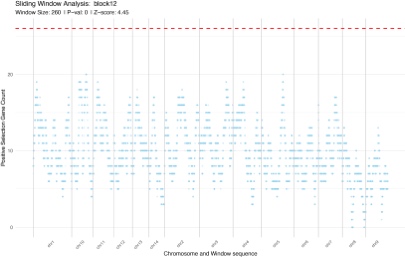 |
| 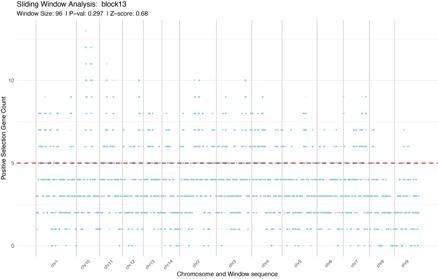 | 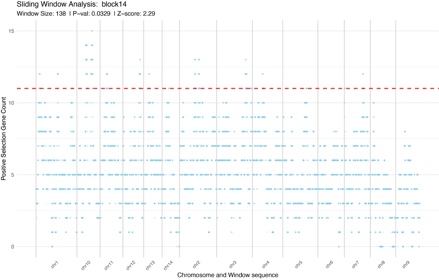 | 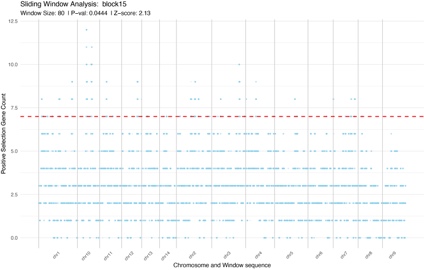 |
| 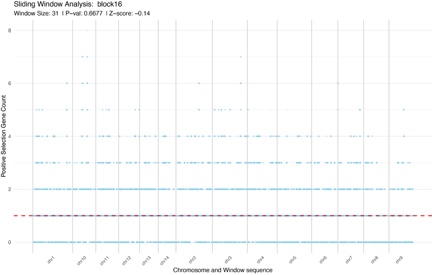 | 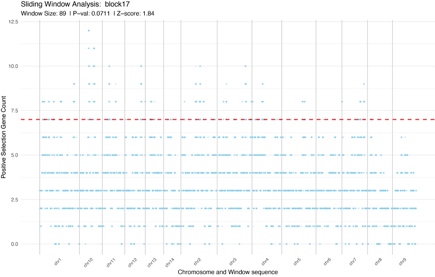 | 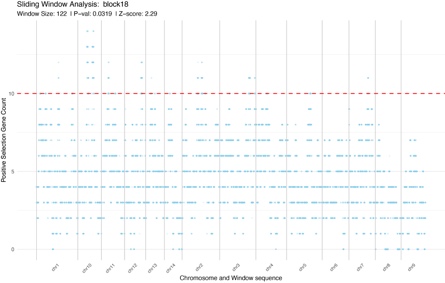 |
| 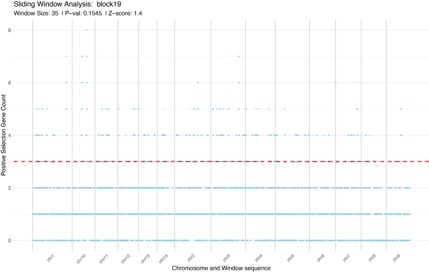 | 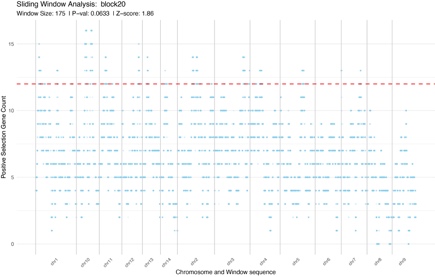 | 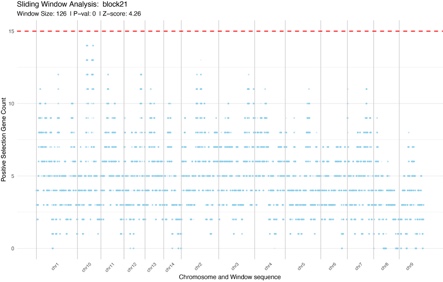 |
| *Pomacea diffusa* |  |  |
| 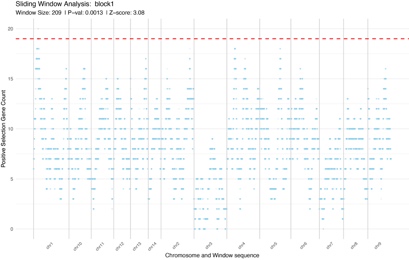 | 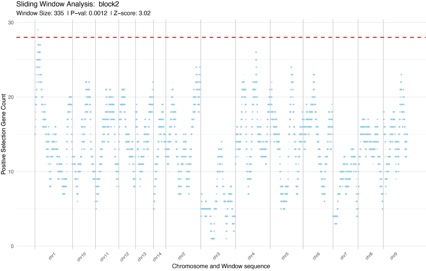 | 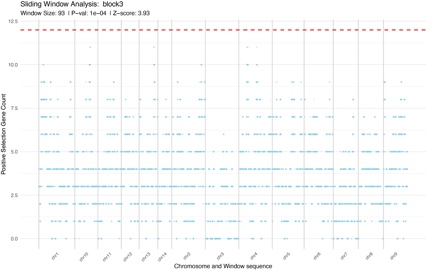 |
| 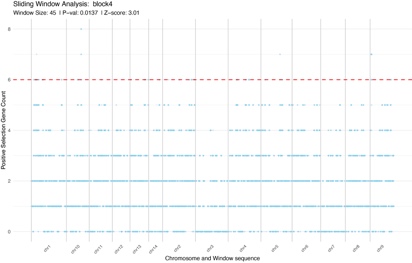 | 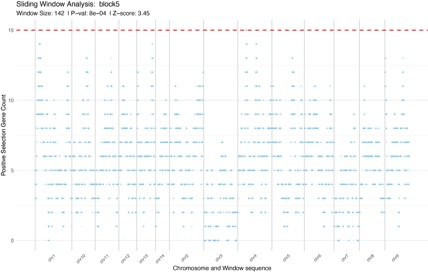 | 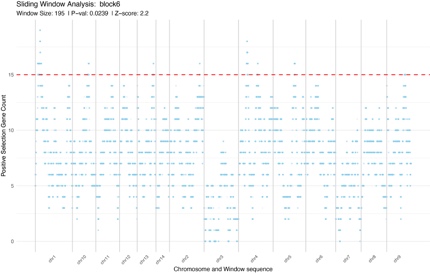 |
| 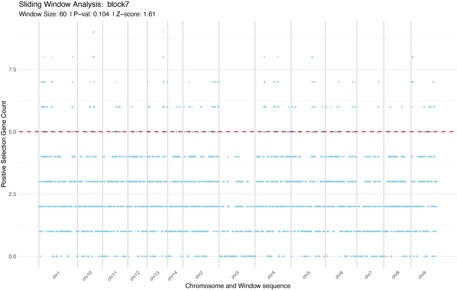 | 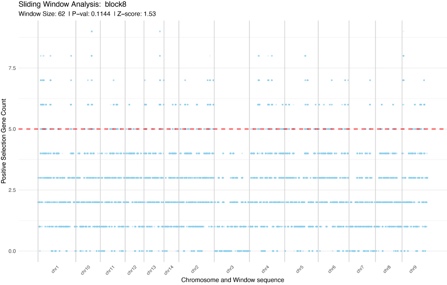 | 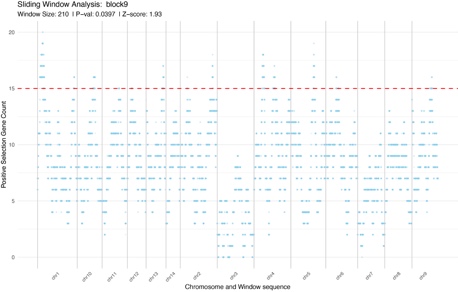 |
| *Marisa cornuarietis* |  |  |
| 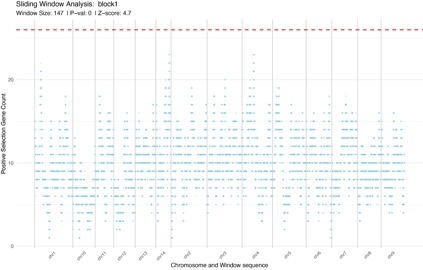 | 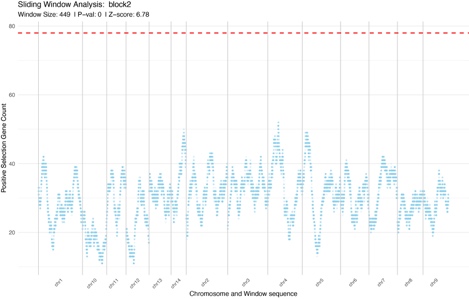 | 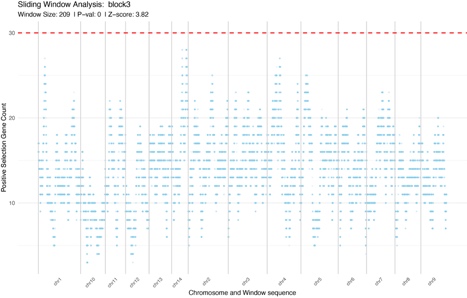 |
| 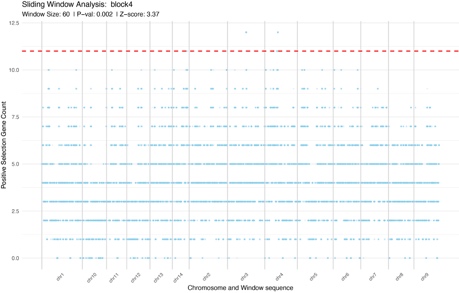 |  |  |

**Fig. S11.** Schematic diagram of the Permutation test in four Ampullariidae species. The abscissa represents the number of chromosomes. The red dotted line represents the number of positively selected genes actually observed in the rearrangement region. The blue dot plot represents the number of positively selected genes in the sliding window simulation of each chromosome.

**
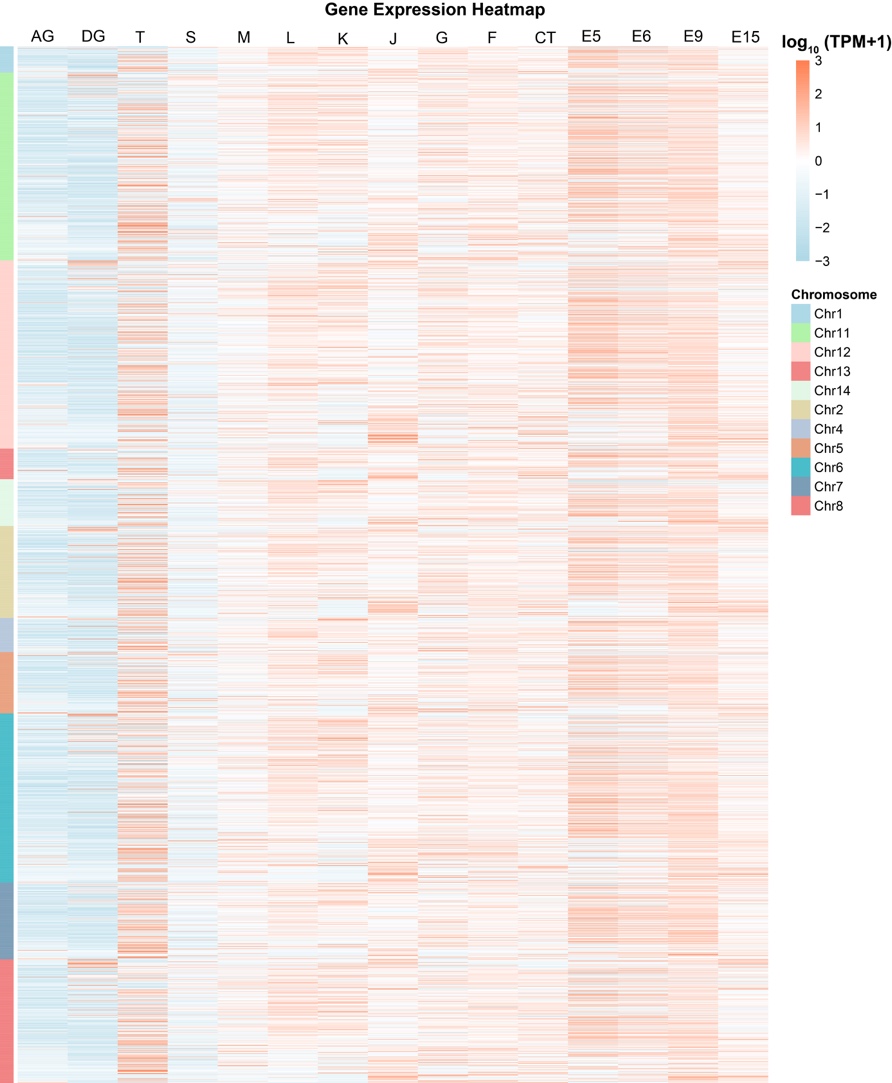
**

**Fig. S12.** Expression level of rearranged genes in different tissues in comparison of *P. canaliculata* and New-world lineage ancestor. Abbreviations of developmental stages/tissues: E5-12, day 5–15 embryo; J, juvenile; AG,albumen gland; DG, digestive gland; K, kidney; S, stomach; T, testis; L, lung; G, gill, M, mantle; F, Foot.

**Fig. S13.** Oxford dotplots of molluscan linkage groups (MLGs) with Viviparidae species, *Bellamya purificata*.


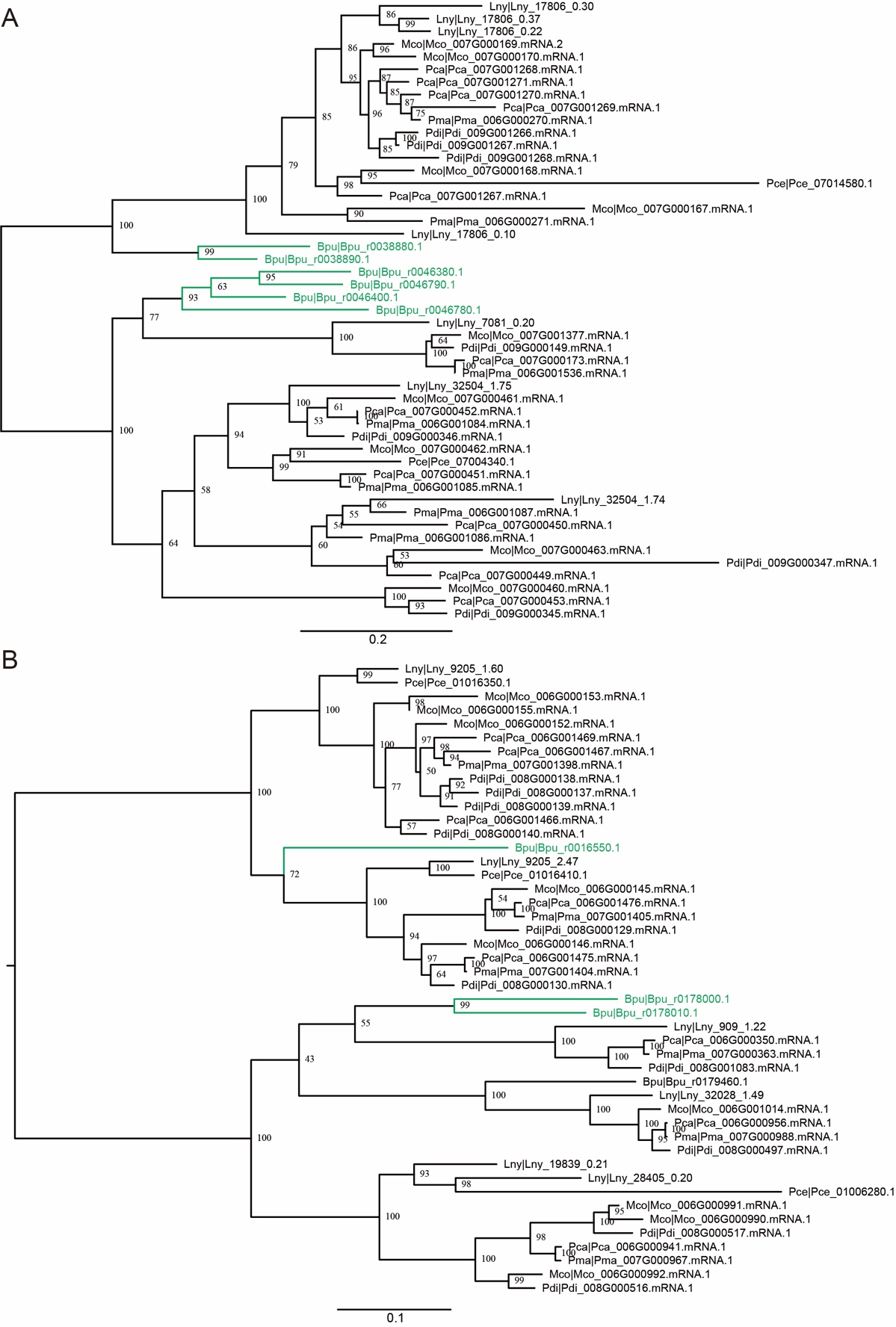


**Fig. S14.** Phylogencitc trees of expanded gene families in Ampullariidae. **(A)** Cellulase gene family and **(B)** β-D-xylosidase gene family. The green branches indicate the outgroup of Ampullariidae – *B. purificata*.


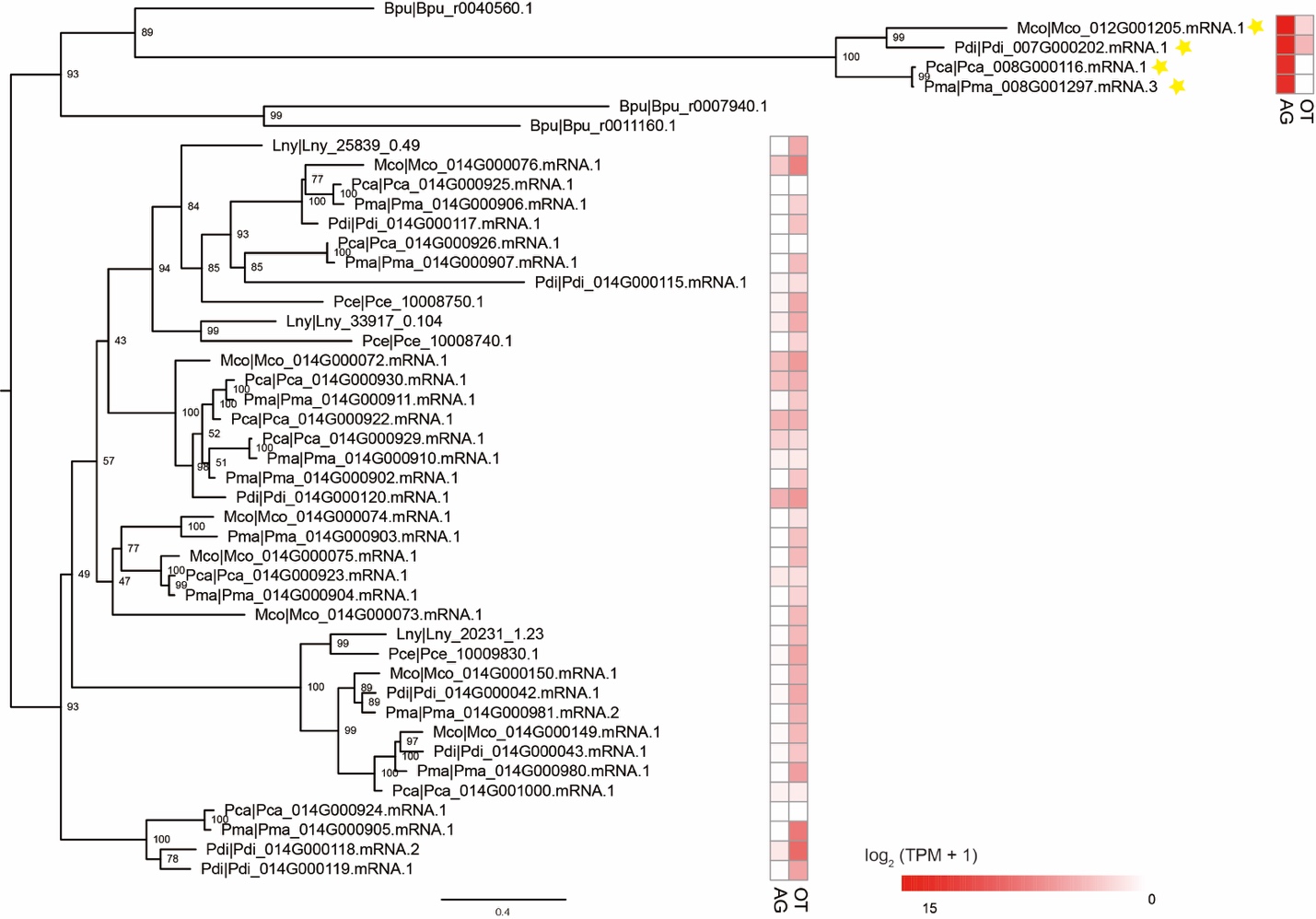


**Fig. S15.** Phylogencitc trees of expanded C1q-related gene family in Ampullariidae. Expression of these genes in the albumen gland (AG) and other tissues (OT) are showed on the right hand side, yellow star indicate PVF proteins identified in PVF MS data (Table. S17). The gene naming rule for New-world species is chr + gene order, so in the gene name the species is written before G and is followed by the chromosome. For example, in Pca|Pca_008G000116.mRNA.1, the corresponding chromosome is chromosome 8.


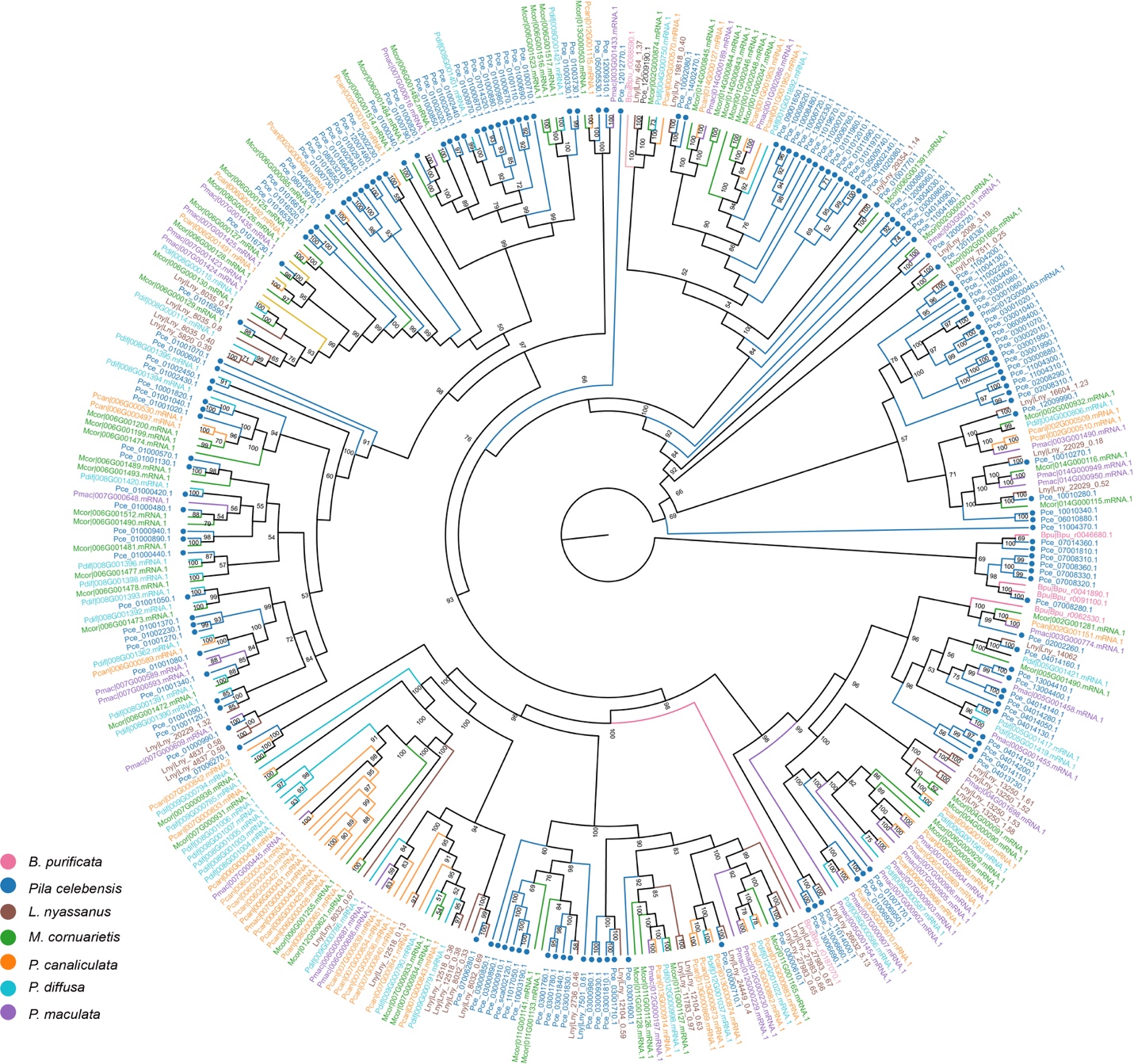


**Fig. S16.** Unrooted maximum-likelihood tree showing the massive expansion of the G-protein coupled receptor (GPCR) family in Ampullariidae and *Bellamya purificata*.


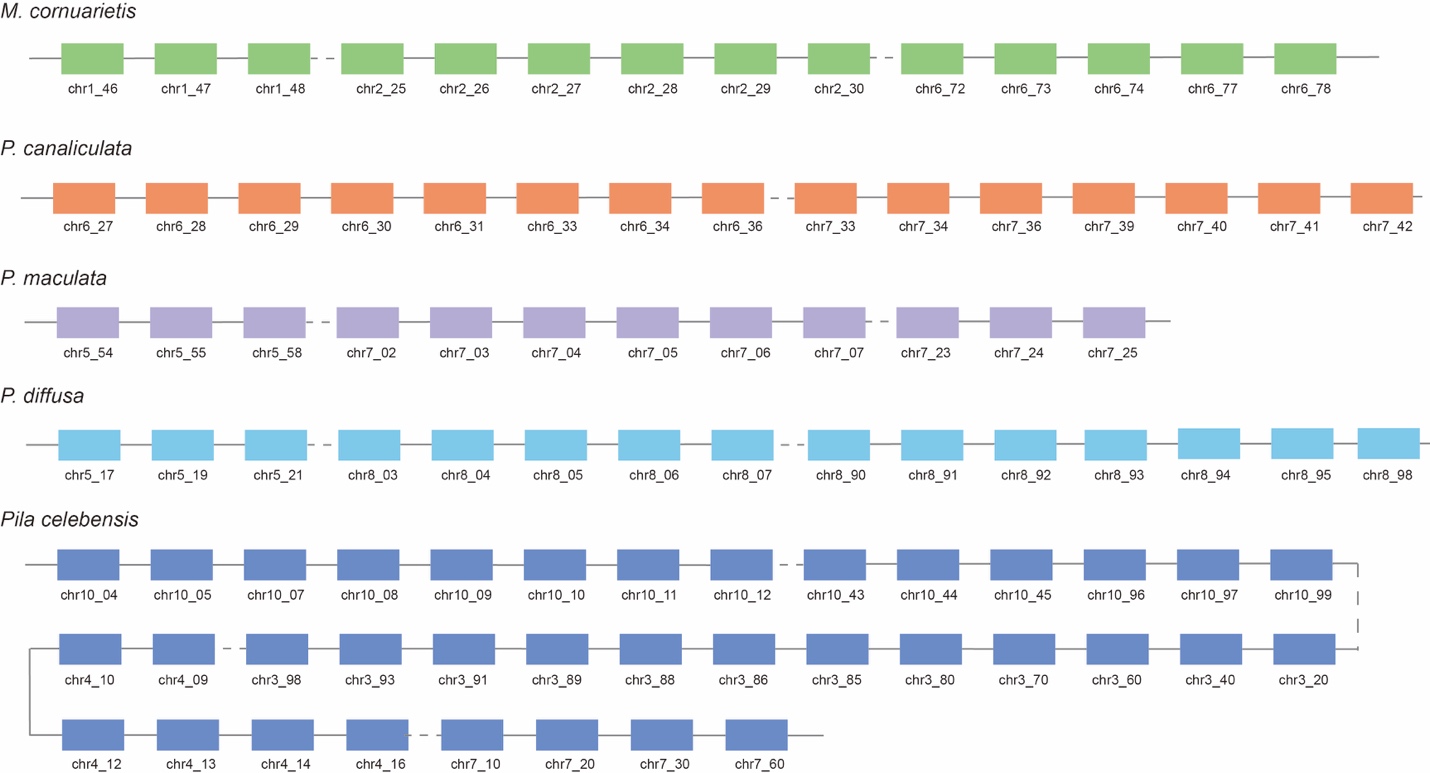


**Fig. S17.** Schematic illustration of GPCR genes in Ampullariidae. Gene names are labeled under the colored graphs and the line length does not represent sequence length.


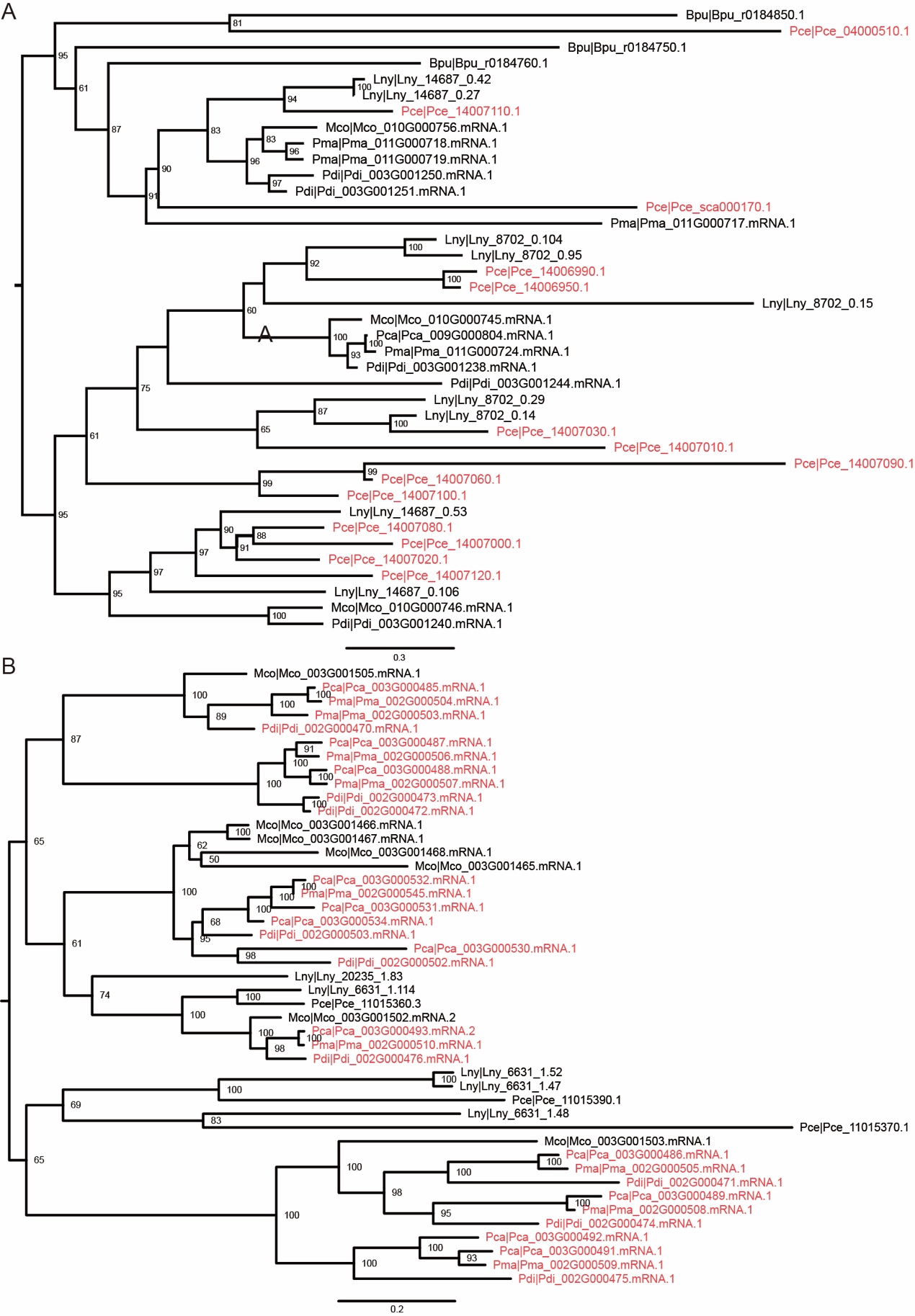


**Fig. S18.** Phylogencitc trees of lineage-specific expanded gene families in *Pila* and *Pomacea* respectively. **(A)** keratin gene family and **(B)** neprilysin gene family. The red branches indicate the expanded genes in *Pila* and *Pomacea*.


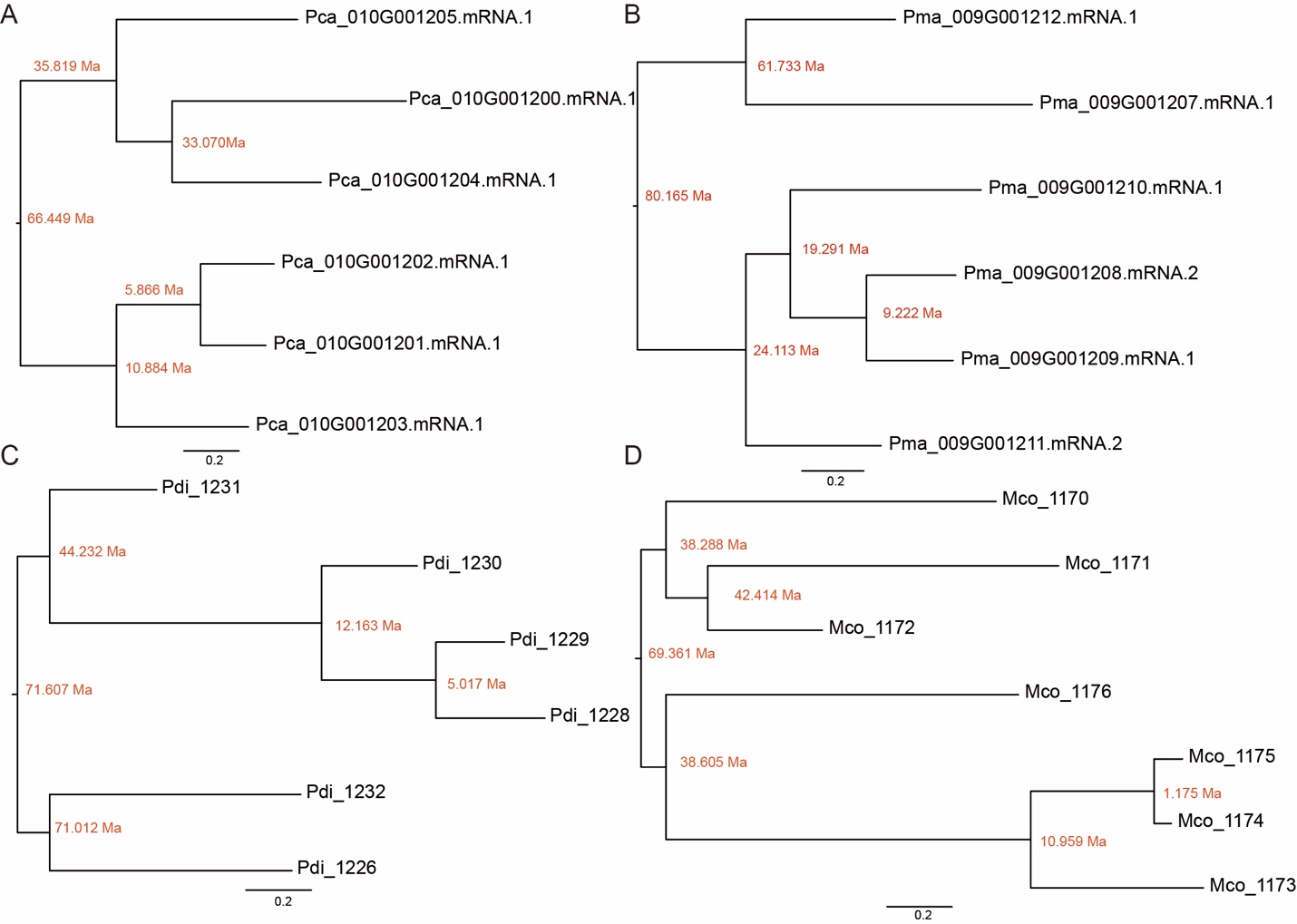


**Fig. S19.** Divergence time of PV1 sequences in four New world species in Ampullariidae. **(A)** *Pomacea canaliculata*, **(B)** *P. maculata*, **(C)** *P. diffusa* and **(D)** *Marisa cornuarietis*. Sequences within the red rectangle belong to clade V of PV1, and the other sequences belong to other four clades. The red number next to nodes are divergent time calculated by KaKs_calculator.


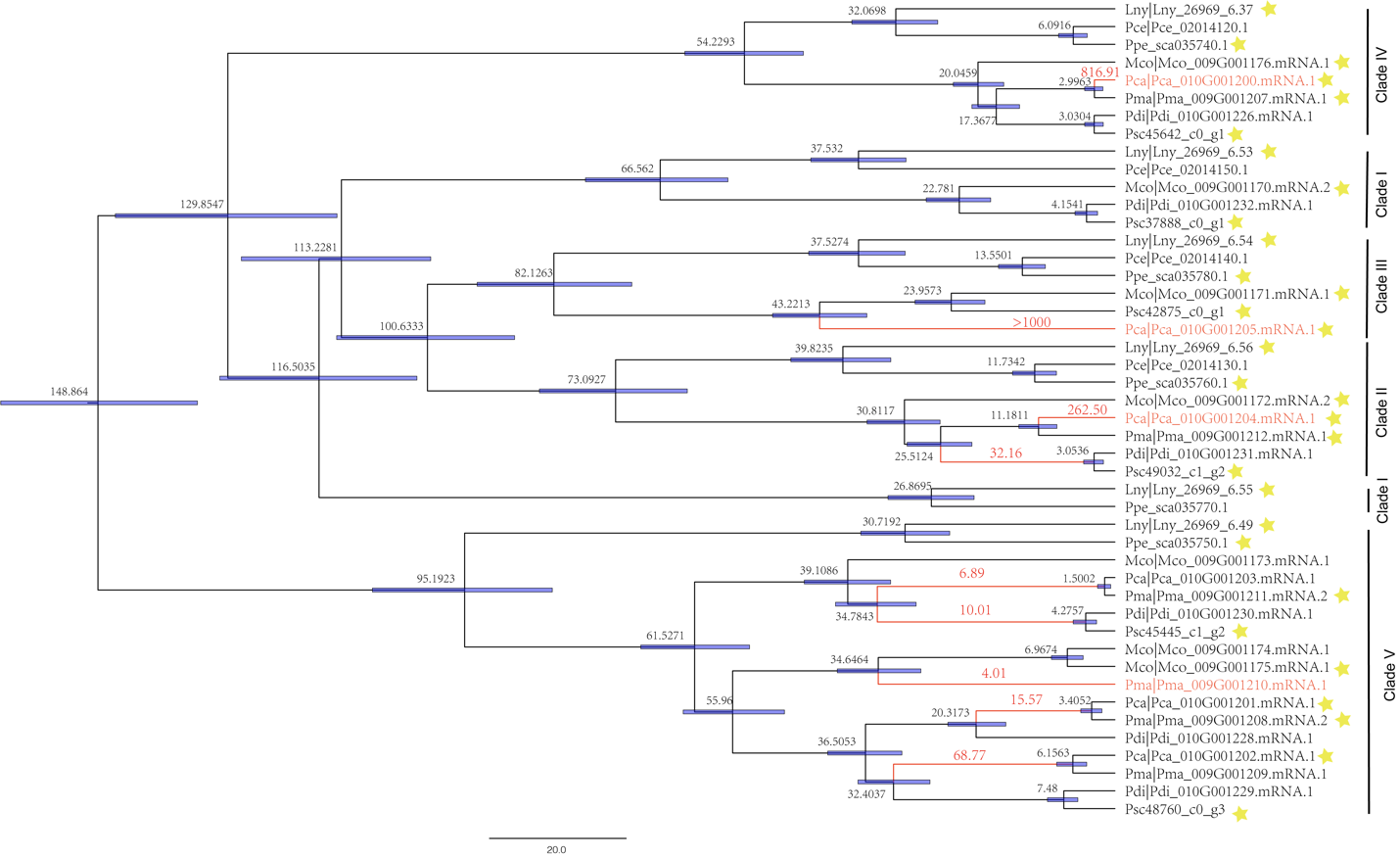


**Fig. S20.** Maximum-likelihood phylogenetic relationships among PV1 sequences in ampullariidae. The tree was calibrated at root node using fossils events to reveal divergence times. Blue lines indicate 95% confidence interval for divergence times. Red branch means under positive selection and the red number on branches are dN/dS values. The yellow star indicate the protein detected in PVF MS data.


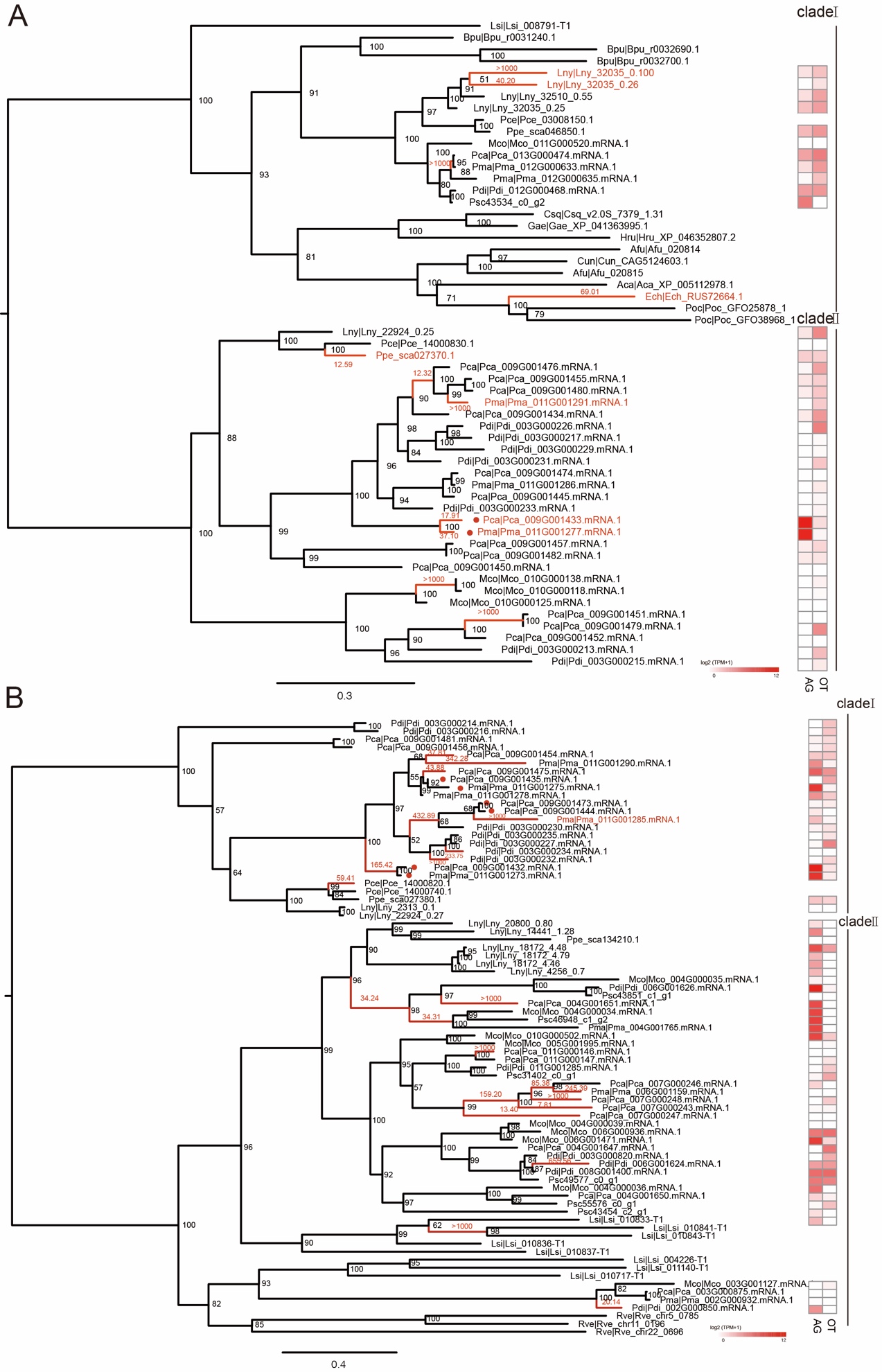


**Fig. S21.** Phylogeny and evolution of PV2 genes in ampullariidae. Phylogeny and expression of homologues of (A) MACPF-like genes and (B) tachylectin-like genes. Numbers on nodes are bootstrap values (>50%) and the red dot preceding each sequence name indicates detection of these proteins in the mass spectrometry (MS) dataset. Red branch means under positive selection and the red number on branches are dN/dS values. Gene expression levels are in logarithmic scale. AG, albumen gland; OT, other tissues.


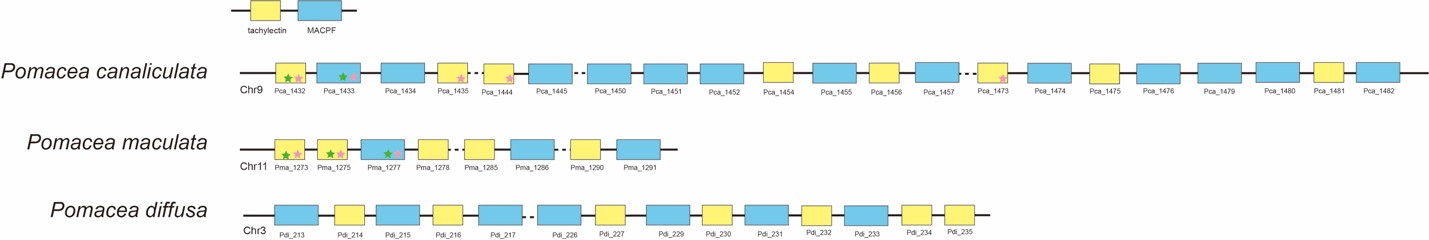


**Fig. S22.** Location of PV2 subunits in three Ampullariidae species.


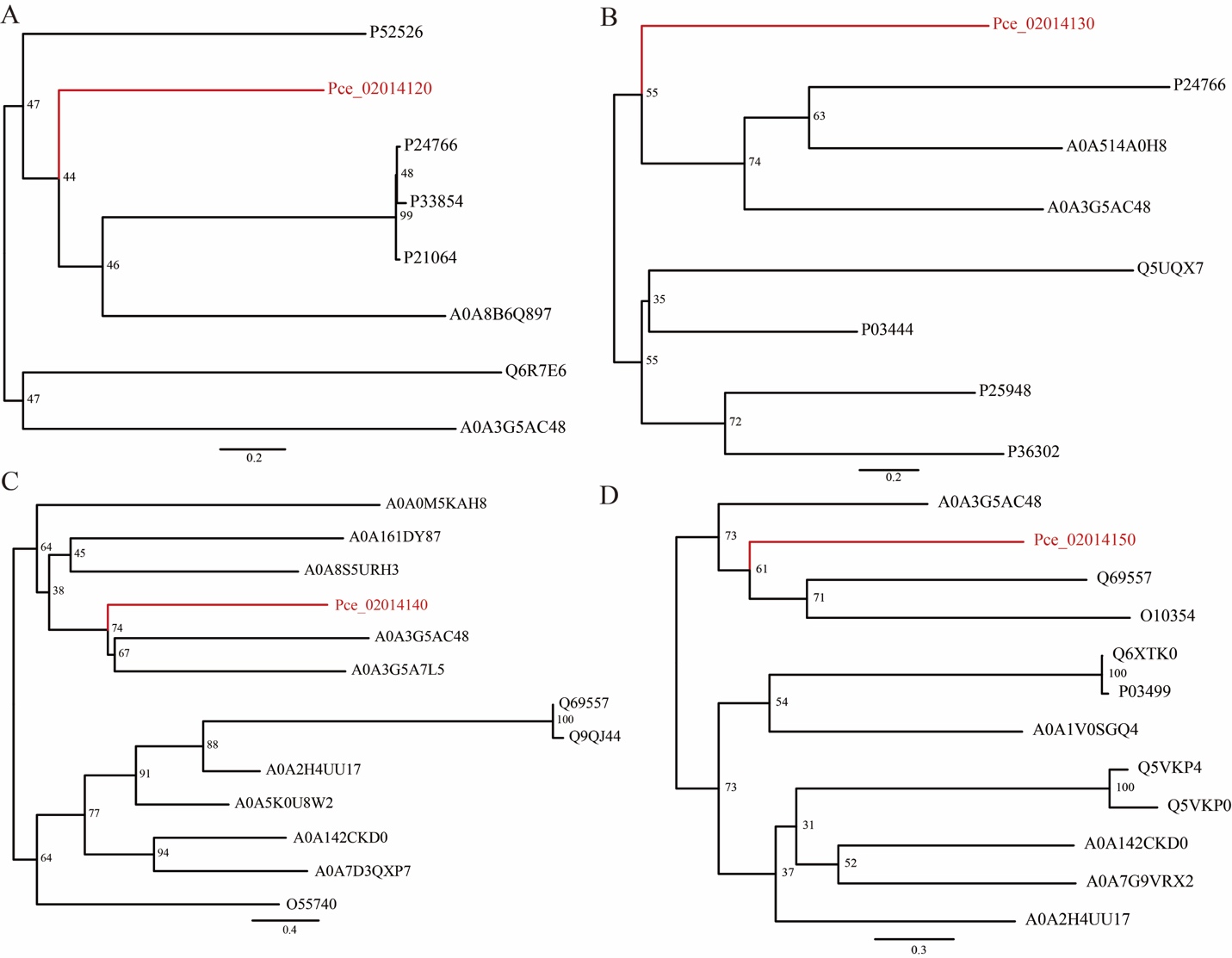


**Fig. S23.** Phylogenetic analysis supporting the horizontal acquisition of PV1 from viruses. Sequences in red represent PV1 sequences from *Pila celebensis*, others represent viral sequences obtained via Foldseek and Protrek.


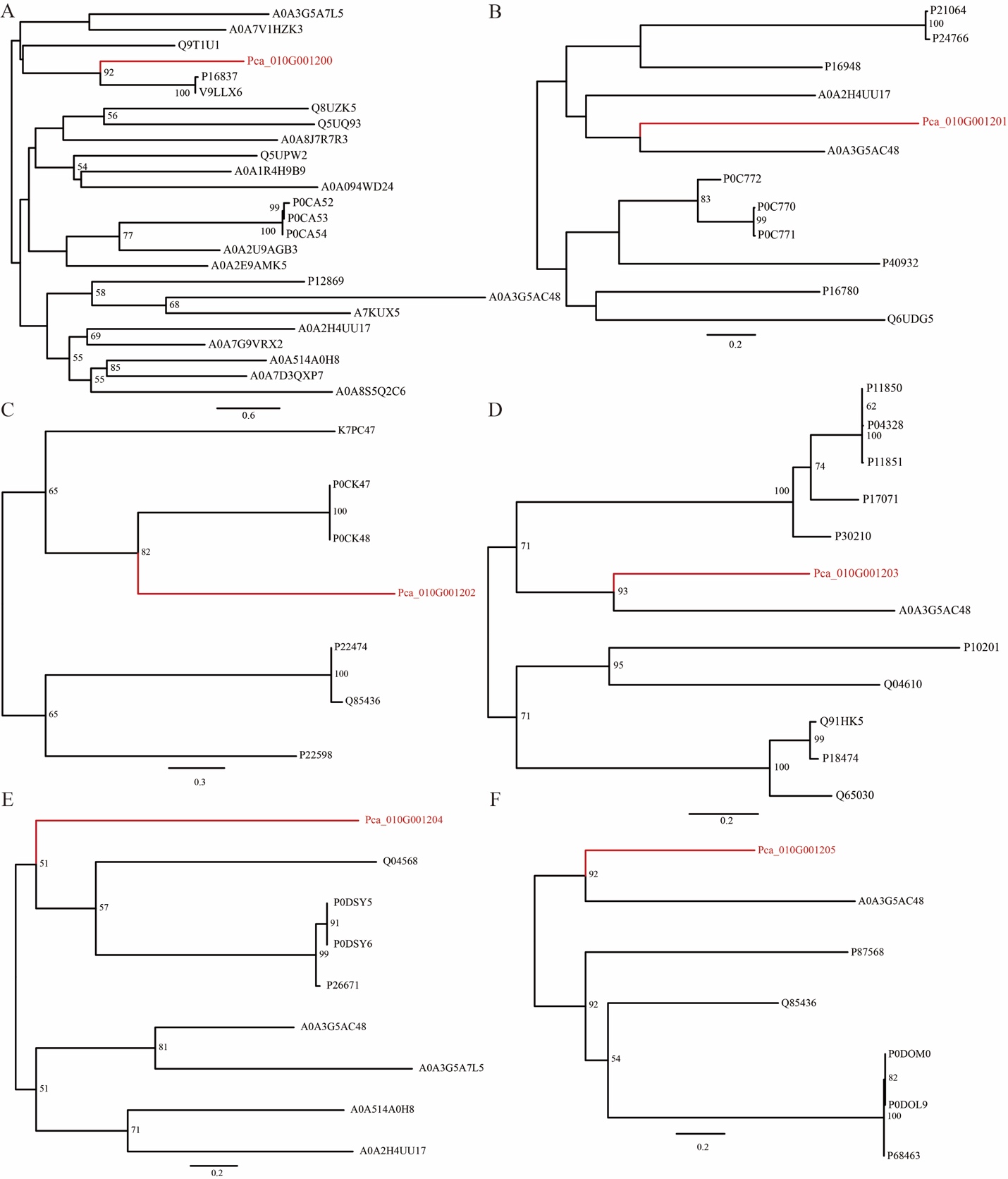


**Fig. S24.** Phylogenetic analysis supporting the horizontal acquisition of PV1 from viruses. Sequences in red represent PV1 sequences from *Pomacea canaliculata*, others represent viral sequences obtained via Foldseek and Protrek.


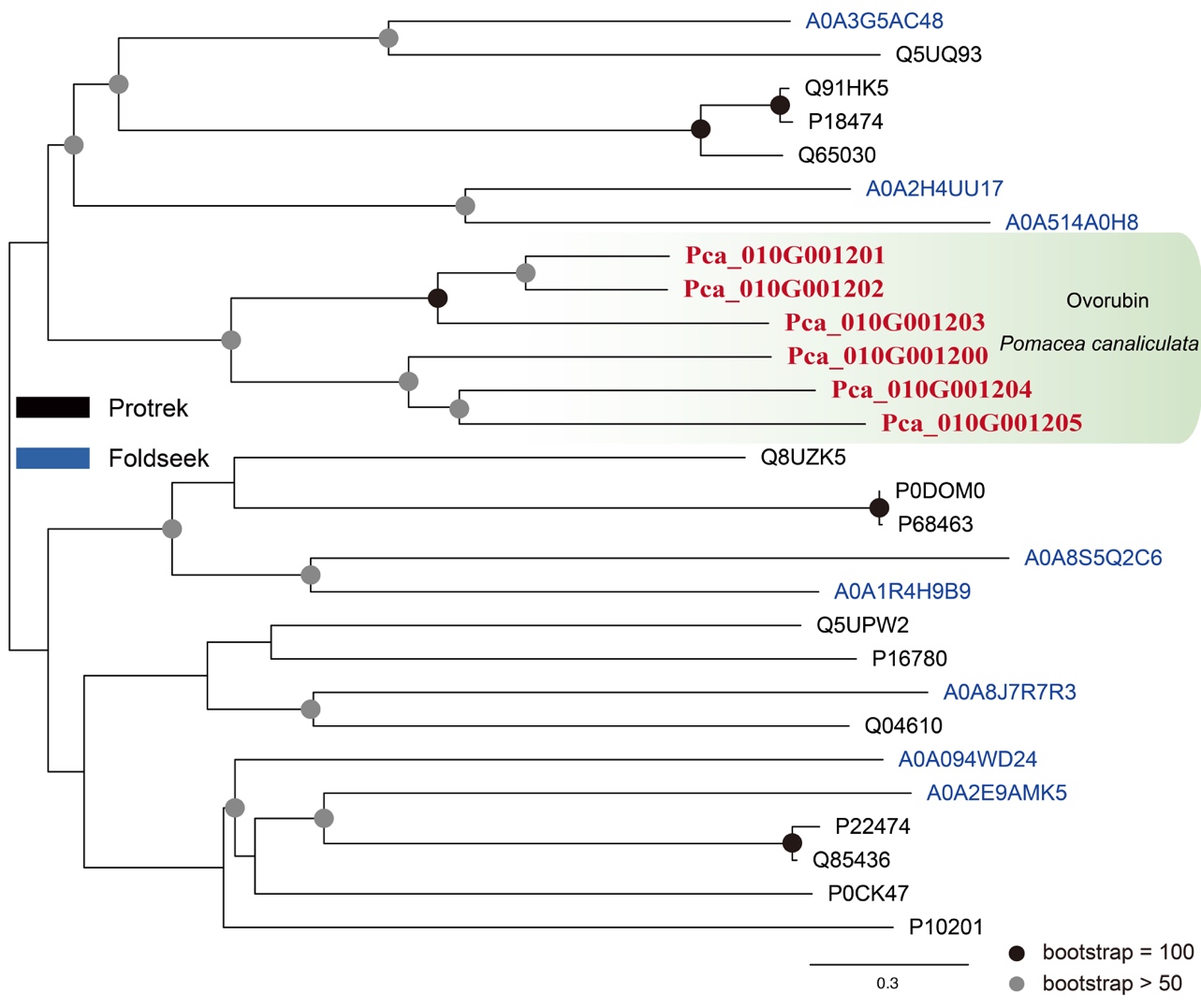


**Fig. S25.** Phylogenetic analysis supporting the horizontal acquisition of PV1 from viruses. Sequences in red represent PV1 sequences from *Pomacea canaliculata*, blue sequences are the viral sequences obtained via Foldseek through 3D structure alignment, and the others are predicted using Protrek.


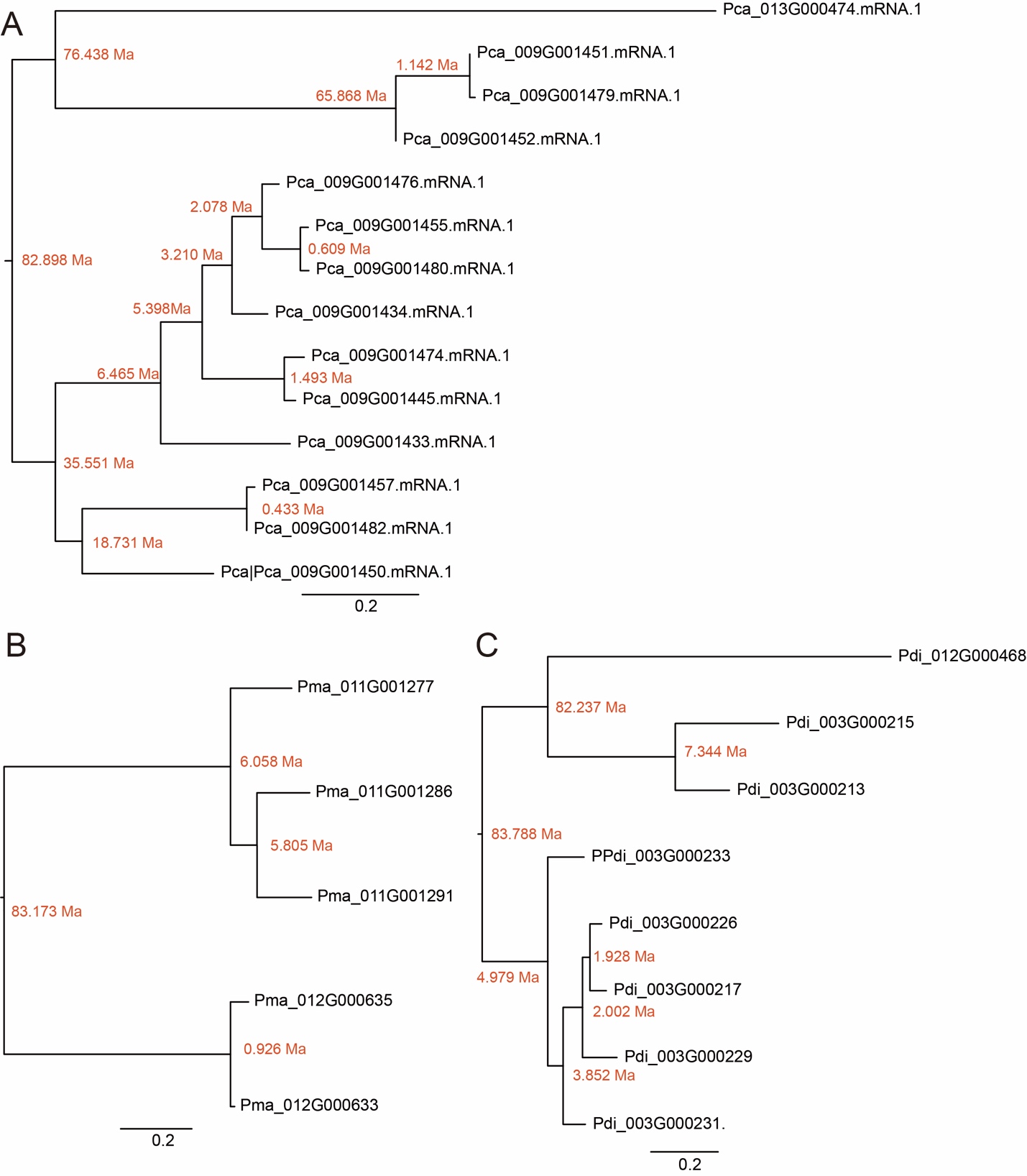


**Fig. S26.** Divergence time of MACPF-like sequences in three New-world species in Ampullariidae. **(A)** *Pomacea canaliculata*, **(B)** *P. maculata*, and **(C)** *P. diffusa*. Sequences within the red rectangle belong to Clade I of MACPF, and other sequences belong to Clade II of MACPF. The red number next to nodes are divergent time calculated by KaKs_calculator.


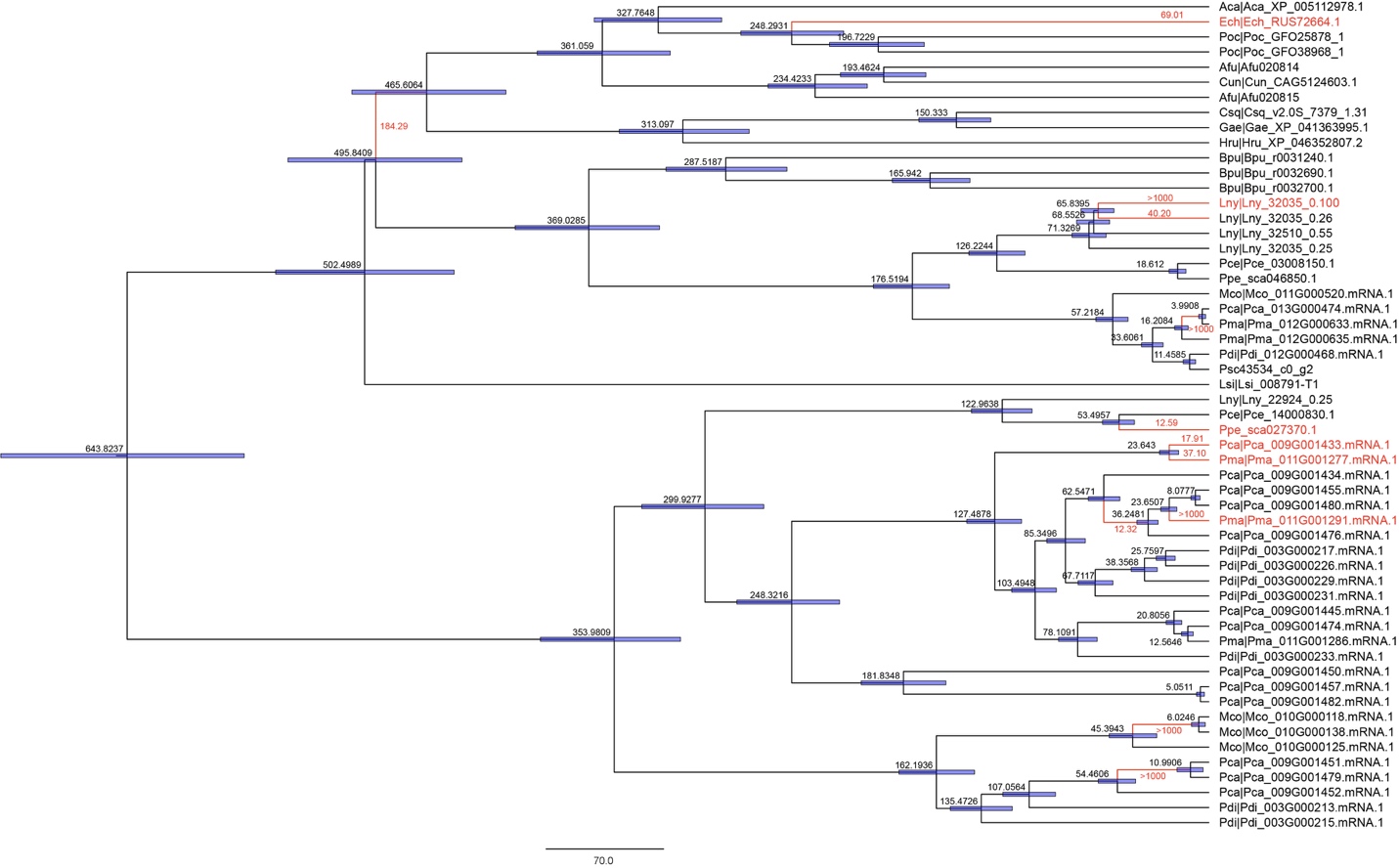


**Fig. S27.** Maximum-likelihood phylogenetic relationships among MACPF sequences in 23 molluscs. The tree was calibrated using fossils events to reveal divergence times. Blue lines indicate 95% confidence interval for divergence times. Red branch means under positive selection and the red number on branches are dN/dS values.


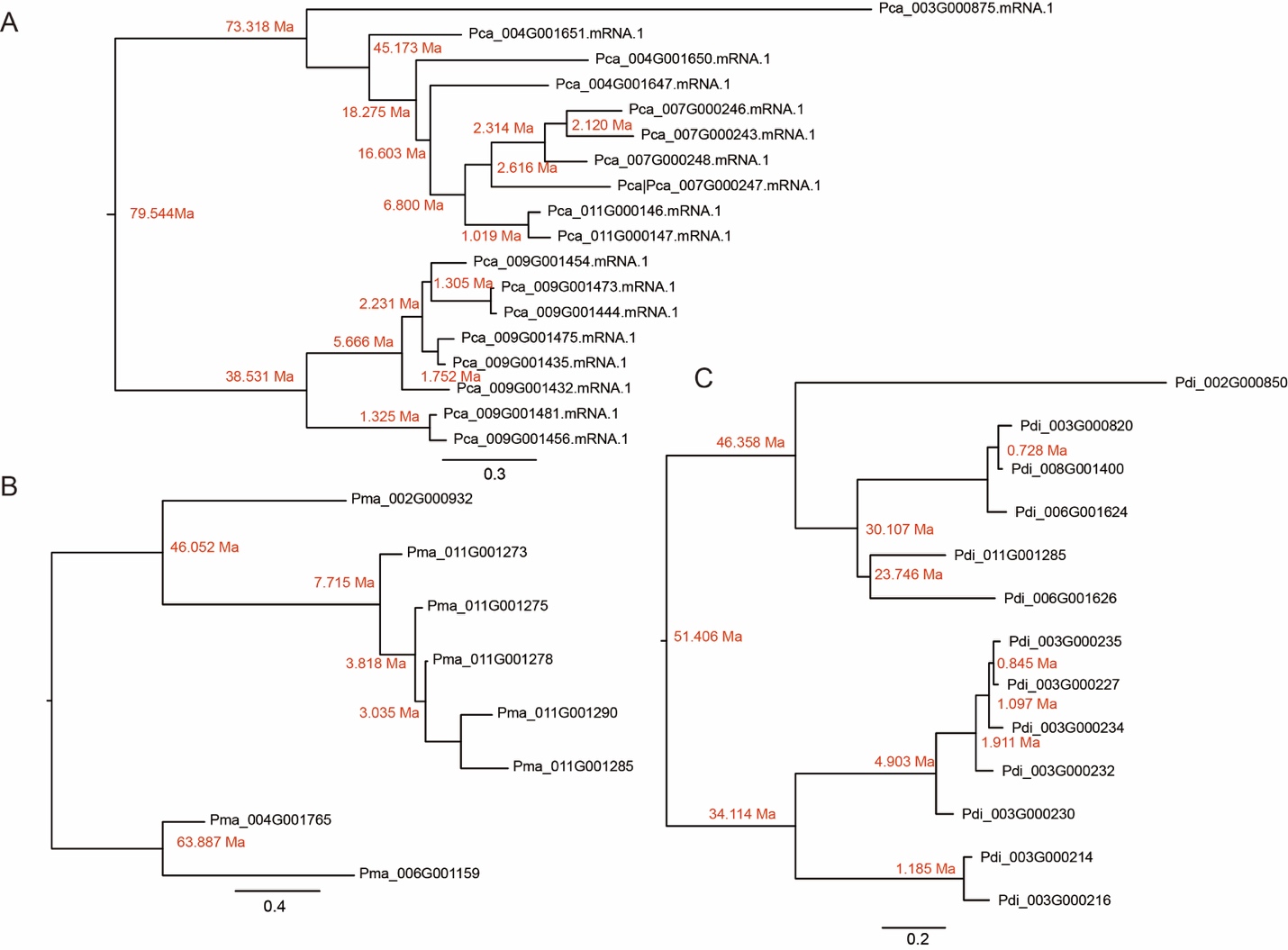


**Fig. S28**. Divergence time of tachylectin-like sequences in three New-world species in Ampullariidae. **(A)** *Pomacea canaliculata*, **(B)** *P. maculata*, and **(C)** *P. diffusa*. The red number next to nodes are divergent time calculated by KaKs_calculator.


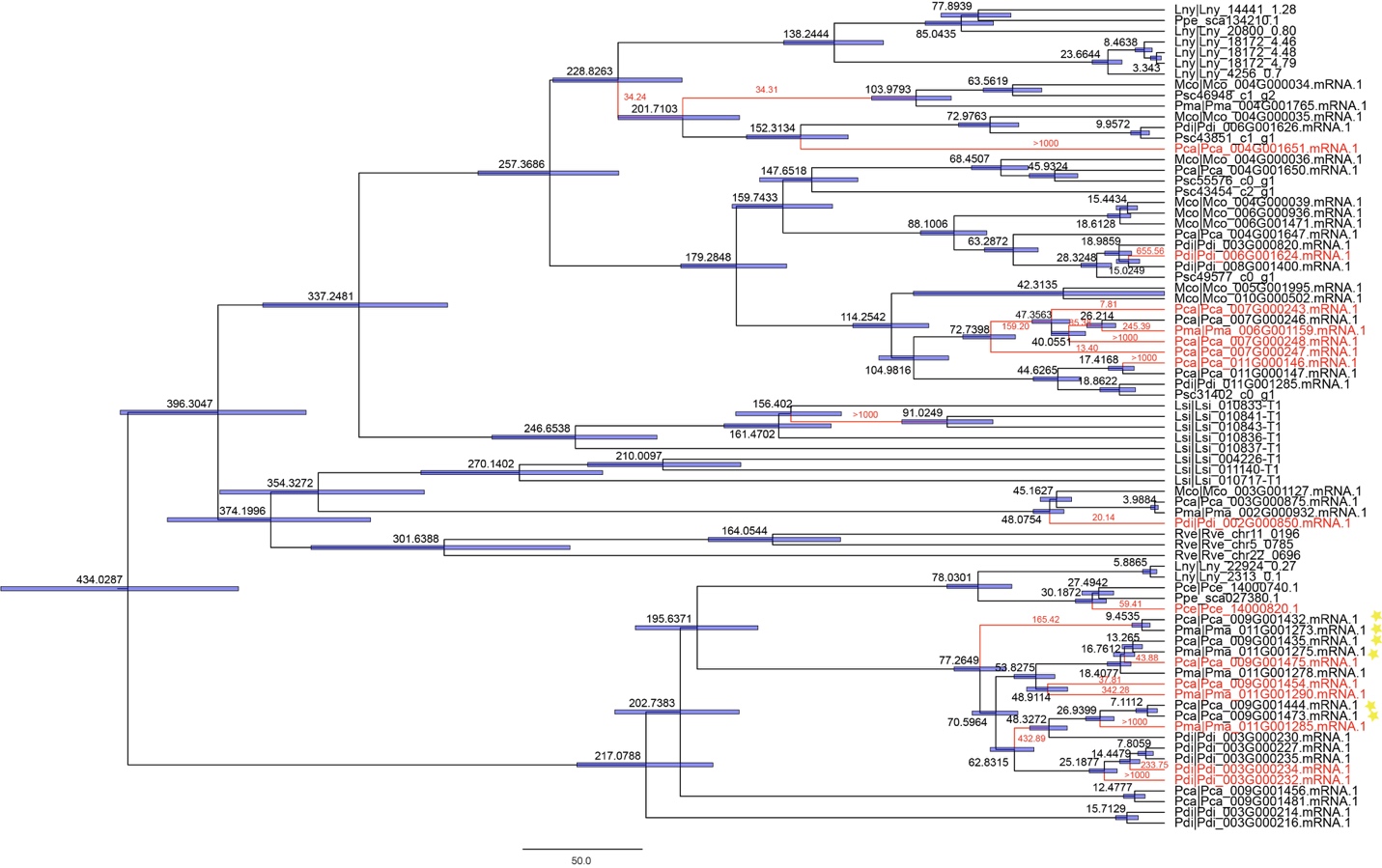


**Fig. S29.** Maximum-likelihood phylogenetic relationships among tachylectin sequences in Caenogastropoda. The tree was calibrated using fossils events to reveal divergence times. Blue lines indicate 95% confidence interval for divergence times. Red branch means under positive selection and the red number on branches are dN/dS values. Yellow star indicates the sequence was detected in protein MS dataset.


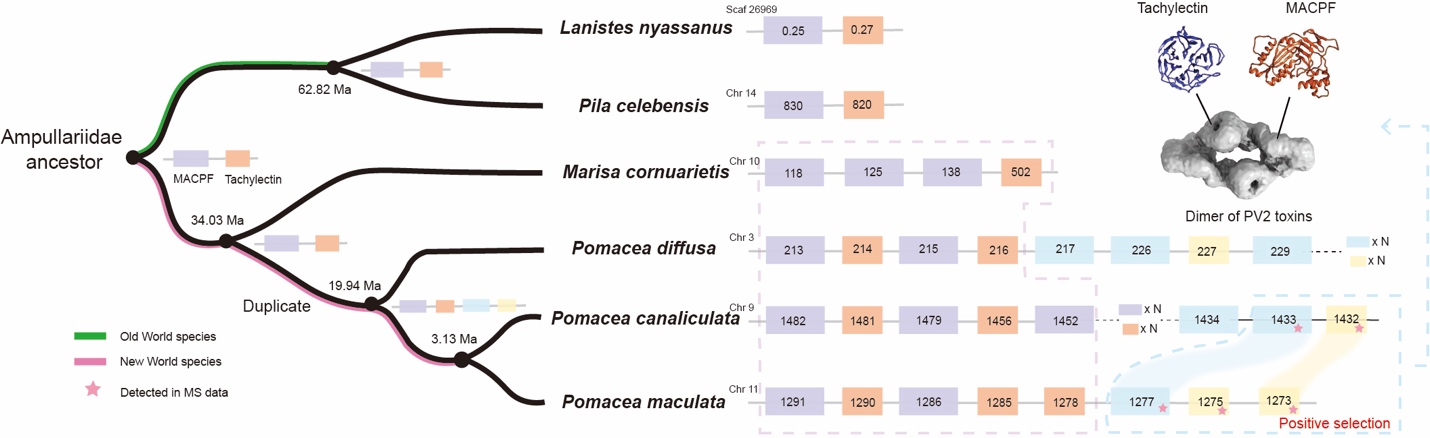


**Fig. S30.** Schematic of PV2 evolution. A two-gene configuration (purple and orange section) indicates the common ancestor of PV2 in Ampullariidae. The MACPF and tachylectin genes framed by a blue dotted line underwent positive selection and contain protein-binding sites for MACPF-tachylectin complexes (Giglio et al., 2020). Pink star indicates highly expressed in the albumen gland.

**3. References**
